# Supplementary material for: Consistent Induction of Broadly Neutralizing HIV Antibodies by a Novel Two-Step Mechanism Informs Immunogen Design
Source: bioRxiv. 2025 Oct 6:2025.10.06.680687. Preprint. [Version 1] doi: 10.1101/2025.10.06.680687 (PMC12632488; doi:10.1101/2025.10.06.680687)
Supplement: Supplement 1 [file media-1.pdf]

Materials and Methods

Non-human primates

Indian rhesus macaques were housed at Bioqual, Inc., Rockville, MD, in accordance with guidelines of the Association for Assessment and Accreditation of Laboratory Animal Care standards. All experiments were approved by the University of Pennsylvania and Bioqual Institutional Animal Care and Use Committees. All incoming animals were healthy and naïve to HIV and SHIV exposure.

Animals in Group 1 were immunized with protein-nanoparticle versions of RC1-3fill (wk -16) and 11MUTB-3fill (wk -8). At each time point, a total dose of 100 ug of immunogen and 750 ug of SMNP adjuvant was given subcutaneously, divided evenly between the four limbs. Four macaques received bolus immunizations (V630, V631, V632, and V633), and four received escalating-dose immunizations (V634, V635, V636, and V637). The escalating dose regimen was administered over the course of two weeks, as described in ref (*103*). Animals in Group 2 were immunized with mRNA-LNP versions of RC1-3fill (wk -16) and 11MUTB-3fill (wk -8). A total dose of 100 ug of immunogen was given at each time point, with 25 ug administered subcutaneously in each upper limb and 25 ug administered intramuscularly in each lower limb.

At wk 0, all animals were infected with either SHIV.5MUT (Group 1-3) or SHIV.BG505.N332 (Group 4). All but two SHIV.5MUT-infected animals (AH82, AG99) received an intravenous infusion of 25 mg/kg of anti-CD8α (Nonhuman Primate Reagent Resource MT807R1) or anti-CD8β (Nonhuman Primate Reagent Resource CD8b255R1) 2-3 days prior to SHIV inoculation to transiently deplete CD8 T cells, with the goal of facilitating viral replication *in vivo* **(Supplementary Table 1)**. All SHIV.5MUT infections were performed intravenously. The SHIV.5MUT inoculum was generated by transfecting 293T cells with the respective infectious molecular clone (50 ng of p27 antigen in RPMI1640 with 10% heat-inactivated fetal bovine serum (FBS)). SHIV.BG505.N332-infected animals were inoculated as described in ref (*48*).

Processing and storage of clinical samples

Blood samples were collected from immunized and infected rhesus macaques into sterile vacutainers containing Anticoagulant Citrate Dextrose Solution A (ACD-A). 40 mL of anticoagulated blood was combined into a sterile 50 mL polypropylene conical tube and centrifuged at 1000 x g for 10 min at 20°C. The plasma phase was collected, clarified by centrifugation, aliquoted into 1 mL cryovials, and stored at -80°C. The WBC and RBC fractions were combined, resuspended in an equal volume of Hanks' Balanced Salt Solution (HBSS) without Mg^{2+}/Ca^{2+} and containing 2 mM EDTA, and distributed evenly into four sterile 50 mL tubes. The volume of each tube was adjusted to 30mL with HBSS-EDTA (2mM), underlaid with 14 mL of 96% Ficoll-Paque (diluted with HBSS-EDTA), and centrifuged at 725 x g for 25 min at 20°C with slow acceleration and braking. Cells at the phase interface were collected, transferred to a sterile 50mL tube, diluted to a final volume of 50 mL with HBSS-EDTA (2mM), and centrifuged at 500 x g for 15 min at 20°C. The cell pellet was resuspended in 30 mL of HBSS with Mg^{2+}/Ca^{2+} and 1% FBS, then centrifuged at 200 x g for 15 min at 20°C. If heavy RBC contamination was observed, lysis was performed using Biolegend RBC Lysis Buffer (420301). The cell pellet was resuspended in 30 mL of HBSS with Mg^{2+}/Ca^{2+} and 1% FBS, and cell count and viability were determined with acridine orange / propidium iodide (AOPI) staining. Cells were centrifuged at 300 x g for 10 min at 20°C, resuspended at 5-10 × 10^6 cells/mL in CryoStor CS5 cell cryopreservation media (Sigma C2999), and aliquoted into 1 mL cryovials (Thermo 374503).

Cells were stored in a CoolCell container (Corning 432008) at -80°C overnight and then transferred to vapor-phase liquid nitrogen for long-term storage. Cells collected from lymph nodes and bone marrow were processed in a similar manner. Lymph nodes were excised and immediately placed in RPMI1640 with 10% FBS on wet ice, cleaned of fat and connective tissue via dissection, quartered, and homogenized through a sterile 100 μm strainer. The homogenized cell suspension was transferred to a 50 mL tube, diluted to a final volume of 30 mL with RPMI with 10% FBS, and subjected to Ficoll density gradient purification as described above. Bone marrow was vigorously resuspended in 20 mL of HBSS without Mg^{2+}/Ca^{2+}, fat and connective tissue was removed, and the resulting single-cell suspension was purified by Ficoll density gradient purification as described above.

Protein expression and purification for nanoparticle preparations and structure determinations

Soluble SOSIP.664 versions (*53*) of BG505, 5MUT, del4, and del8 native-like gp140 Env trimers were expressed with MD39 stabilizing mutations (*24*) and potential N-linked glycosylation sites (PNGSs) introduced at positions 230_{gp120}, 241_{gp120}, and 344_{gp120} ("3fill" substitutions) to shield an immunodominant off-target glycan hole (*26, 69, 70*) as described (*104*). SpyTagged SOSIP Env trimers were prepared by adding a 16-residue SpyTag003 sequence (*105*) to the C-terminus of each soluble Env trimer. Soluble SOSIP Envs were expressed by transient transfection in Expi293 cells (Life Technologies). Proteins were harvested from culture supernatants and purified using PGT145 immunoaffinity chromatography followed by size-exclusion chromatography (SEC) on Superose 6 (GE Healthcare) in 20 mM sodium phosphate pH 7.5, 150 mM NaCl (PBS) as described (*104*). Final protein preparations were stored at 4°C. Fab fragments corresponding to the AJ09-21, AJ09-83, AJ09-110, AM12-340, AM12-347, AM12-351, AM12-352, NN39-25, V634-136, V634-136 UCA, V645-158, NN39-25, and NN39-171 IgGs were generated as described (*106*). In brief, Expi293 cells were transiently transfected with plasmids encoding an IgG light chain and the Fab region of an IgG heavy chain including a C-terminal 6x-His tag. Fabs secreted into the culture medium were isolated by nickel-nitrilotriacetic acid (Ni^{2+}-NTA) affinity chromatography (GE Healthcare) followed by purification via SEC using a Superdex 200 16/60 column (GE Healthcare). Purified Fabs were concentrated and stored at 4°C in a buffer containing 20 mM Tris pH 8.0, 150 mM NaCl (TBS) and 0.02% sodium azide.

Preparation of SOSIP-mi3 nanoparticles

SpyCatcher003-mi3 particles were prepared by purification from BL21 (DE3)-RIPL *E. coli* (Agilent) transformed with a pET28a SpyCatcher003-mi3 gene (*107*) (including an N-terminal 6x-His tag) as described (*108*). Briefly, bacterial cell pellets were lysed with a cell disruptor in the presence of 2.0 mM PMSF (Sigma) and then spun at 21,000 x g for 30 min and filtered with a 0.2 μm filter. mi3 particles were isolated by ammonium sulfate precipitation followed by SEC using a HiLoad 16/600 Superdex 200 (GE Healthcare) column equilibrated with TBS pH 8.0. SpyCatcher003-mi3 particles were stored at 4 °C and used for conjugations after filtering with a 0.2 μm filter and spinning for 30 min at 4 °C and 14,000 x g. For preparation of SOSIP-mi3 nanoparticles, SpyCatcher003-mi3 was incubated with a 2-fold molar excess (SOSIP protomer to mi3 subunit) of purified SpyTagged SOSIP overnight at room temperature in PBS. Conjugated SOSIP-mi3 particles were separated from free SOSIPs by SEC on a Superose 6 10/300 column (GE Healthcare) in PBS. Fractions corresponding to conjugated mi3 particles were identified by SDS-PAGE. Concentrations of SOSIP-mi3 particles were determined using the absorbance at 280 nm as measured on a Nanodrop spectrophotometer (Thermo Scientific).

mRNA design and production

Plasmids encoding membrane-anchored prefusion-stabilized Env trimers matching RC1 and 11MUTB protein immunogens were designed, cloned (Genscript), and used as a template for *in vitro* transcription (**fig. S1A**). Nucleoside-modified mRNA immunogens were produced using T7 RNA polymerase (Megascript) with co-transcriptional RNA capping (CleanCap 3OMe, Trilink) and incorporation of N1-methylpseudouridine in place of uridine (Trilink) as previously reported (*109, 110*). A plasmid-encoded polyadenylation sequence (101 nt) was incorporated to improve mRNA stability within the cell. Contaminating double stranded byproducts were removed with cellulose purification as previously described (*111*) and innate immune sensing was assessed as interferon-alpha release following transfection of monocyte-derived dendritic cells (*112*). mRNA was evaluated for appropriate size using gel electrophoresis and stored at -20°C.

mRNA-LNP Synthesis

mRNA-LNPs were prepared in 4-component lipid nanoparticles using previously described methods (*113, 114*). LNP formulations contain ionizable lipid (proprietary to Acuitas) / phosphatidylcholine / cholesterol / PEG-lipid. Lipid and LNP compositions are described in US patent US10,221,127.

SHIV and pseudovirus production

The construction of SHIV.BG505.N332 was described previously and includes an S375Y mutation that is required for efficient entry and replication in rhesus CD4 T cells (*115*). SHIV.5MUT was generated by mutagenizing four residues (V134Y, N136P, I138L, and D140N) in the plasmid encoding this parental strain using the Q5 Site-Directed Mutagenesis Kit (NEB E0554S). Mutants in this and other backbones were generated using the same strategy. Mutagenized plasmids were sequenced to confirm their integrity.

Infectious SHIV stocks were produced as previously described (*115*). Briefly, 5×10^6 HEK 293T cells (ATCC CRL-3216) were plated in a 100 mm tissue culture dish in 10 mL of Dulbecco's Modified Eagle Medium (DMEM) supplemented with 10% FBS and 100 U/mL penicillin-streptomycin (Gibco 10378-016) and cultured at 37°C with 5% CO_2. After 24 hours, 6 ug of SHIV plasmid DNA (for SHIV generation) or 4.5 ug of HIV-1 backbone (SG3ΔEnv) plasmid plus 1.5 ug of Env plasmid (for pseudovirus generation) was diluted in 500 uL of DMEM, combined with 18 uL of FuGENE 6 transfection reagent (Promega E2692), and added dropwise to the cells. Cells were cultured for 48 hours at 37°C with 5% CO_2. Virus-containing media was then harvested and centrifuged at 2000 x g for 8 min at 20°C. Supernatant was divided into 500 uL aliquots and stored at -80°C. SHIV infection stocks were quantified by measuring p27 antigen using an ELISA (ZeptoMetrix 0801169). Pseudovirus stocks were titrated in TZM-bl cells (*116*). Briefly, 1.5×10^4 TZM-bl cells were seeded in flat-bottom 96-well tissue culture plates in DMEM with 10% FBS and 100 U/mL penicillin-streptomycin and cultured at 37°C with 5% CO_2. Virus stocks were harvested 24 hours later, serially five-fold diluted in DMEM with 6% FBS, 100 U/mL penicillin-streptomycin, and 40 ug/mL DEAE-dextran, and then added to the cells with four technical replicates. 48 hours later, cells were fixed in phosphate-buffered saline (PBS) containing 0.8% glutaraldehyde and 2.2% formaldehyde for 10 min, washed three times with PBS, and stained with PBS containing 4 uM magnesium chloride, 4 uM, potassium ferricyanide, 4 uM potassium ferrocyanide, and 400 ug/mL X-gal for 3 hours at 37°C. Cells were washed three times with PBS and imaged on an ELISPOT analyzer (CTL ImmunoSpot 7.0.34.0 Professional Analyzer DC).

Infectious virus titers were determined by averaging the number of spots per virus dilution and dividing by the viral input volume.

Plasma viral RNA quantification

SHIV plasma viral load was quantified using standardized, quality-controlled real-time PCR assays (Applied Biosystems) at the CLIA-certified, NIH/NIAID-sponsored Non-Human Primate Virology Core Laboratory at the Duke Human Vaccine Institute. High-throughput sample processing and PCR setup was facilitated by use of QIAsymphony SP and QIAgility automation platforms (QIAGEN). Viral RNA was isolated from plasma and reverse-transcribed into cDNA using a target-specific primer. cDNA was treated with RNase, added to a custom real-time PCR master mix containing target-specific primers and a fluorescently-labeled hydrolysis probe, and amplified on a QuantStudio3 (Thermo) real-time quantitative PCR machine. Raw data were quality-controlled (including confirmation of positive and negative controls) and mean viral RNA copies/mL was calculated. The lower limit of quantification of this assay was 62 RNA copies/mL.

Neutralization assay

Neutralization assays were performed using TZM-bl indicator cells as previously described (*115-117*). Briefly, 1.5×10^4 TZM-bl cells were plated in flat-bottom 96-well plates in 100 uL of Dulbecco's Modified Eagle Medium (DMEM) supplemented with 10% FBS and 100 U/mL penicillin-streptomycin and cultured at 37°C with 5% CO_2. 24 hours later, plasma samples were heat-inactivated at 56°C for 1 hour and serially five-fold diluted starting at 1:20. To maintain a constant plasma concentration of 5% v/v across all wells, media was supplemented with 10% human serum (Millipore Sigma H5667) at all but the highest dilution. Alternatively, monoclonal antibodies were serially five-fold diluted starting at 50 or 250 ug/mL in media that was not supplemented with human serum. Virus was diluted to achieve a final multiplicity of infection (MOI) of 0.3 when added to the TZM-bl cells. Virus was incubated with plasma or mAb dilutions at 37°C for 1 hour, and this mixture was then added to the adherent TZM-bl cells and cultured at 37°C with 5% CO_2. 48 hours later, cells were lysed with 0.5% Triton-X 100 in PBS and luciferase activity was quantified using a luciferase assay reagent (Promega E1501) and a BioTek Synergy Neo2 plate reader (Agilent).

Generation of soluble Env trimers for flow cytometry

Stabilized HIV Env trimers were expressed and purified as previously described. A V3 knockout construct was generated by introducing D325A, R327A, and S334A mutations into the Q23 backbone. Plasmids encoding Env trimers with C-terminal AviTags were transfected into HEK293F cells (Thermo R79007) cultured in Gibco FreeStyle 293 Expression Medium (Gibco 12338018) using PEI-MAX 4000 transfection reagent (Polysciences 23765). *Escherichia coli* biotin ligase (BirA) was co-transfected to site-specifically biotinylate the biotin-acceptor AviTag peptide on Env. Cells were maintained at 37°C with 8% CO_2 and shaking at 125 rpm. Four days post-transfection, cultures were harvested by centrifugation at 2700 x g for 15 min and clarified supernatants containing secreted Env trimers were collected. Env trimers were purified by affinity chromatography using either agarose-bound Galanthus nivalis lectin (GNL; Vector Laboratories AL-1243-5) or Toyopearl AF-Tresyl-650M resin (Tosoh Bioscience 0014472) conjugated to the broadly neutralizing antibody PGT145. Antibody conjugation to the resin was performed according to the manufacturer's protocol. Eluted proteins were further purified by size-exclusion chromatography (SEC) using a Superdex 200 Increase 10/300 GL column (Cytiva 28-9909-44)

equilibrated in PBS or Tris-buffered saline (TBS). Biolayer interferometry (BLI) was used to assess the efficiency of biotinylation with an Octet RH96 instrument (Sartorius). Biotinylated Env proteins were diluted in PBS supplemented with 0.1% Tween-20 and analyzed using streptavidin biosensors (Sartorius 18-5019) pre-equilibrated in the same buffer. Successful biotinylation was confirmed by a reproducible and saturable loading response within the expected range for mono-biotinylated proteins. Purified biotinylated Envs were conjugated to streptavidin-BV421 (Biolegend 405225), streptavidin-AF647 (Biolegend 405237), or streptavidin-PE (Invitrogen SA10044) by incubating together at a 4:1 molar ratio at 4°C.

B cell isolation

5-10 × 10^6 cryopreserved PBMCs were thawed and resuspended in 10mL of RPMI supplemented with 10% FBS, 100 U/mL penicillin-streptomycin, and *** Benzonase (Millipore Sigma 70664-3). The cell suspension was centrifuged at 300 x g for 5 min at 4°C, washed once with PBS, and stained with Zombie-NIR viability dye (Biolegend 77184) for 30 min at 4°C. Cells were washed with PBS supplemented with 5% FBS and stained for 1 hr at 4°C with a cocktail of antibodies against CD3 (clone SP34-2, BD 557757), CD8a (clone SK1, Invitrogen 47-0087-41), CD14 (clone 61D3, Invitrogen 47-0149-42), CD16 (clone eBioCB16, Invitrogen 47-0168-41), CD20 (clone L27, BD 335793), and IgG (clone G18-145, BD 550931). For isolation of bulk B cells for lineage tracing analysis, antibodies against IgM (clone G20-127, BD 562618) and IgD (polyclonal, Dako F0189) were also used. For isolation of single, Env-specific B cells, cells were washed once with PBS supplemented with 5% FBS and then were stained for 1 hr at 4°C with 1 ug of BV421-, AF647, and/or PE-conjugated soluble Env trimer in PBS supplemented with 5% FBS. All cells were then washed three times with PBS supplemented with 5% FBS, filtered through a sterile 100 µm strainer, and sorted on a BD FACSMelody Cell Sorter. Naïve ($CD3^-CD8a^-CD14^-CD16^-CD20^+IgM^+IgD^+$) or memory ($CD3^-CD8a^-CD14^-CD16^-CD20^+IgM^-IgD^-IgG^+$) B cells were sorted in bulk into filtered FBS, centrifuged at 300 x g for 5 min, resuspended in 600 uL of cold RNAzol RT (Molecular Research Center RN190), transferred into 2 mL vials (Sarstedt 72.694.396), and stored at -80°C. Single, Env-specific B cells ($CD3^-CD8a^-CD14^-CD16^-CD20^+IgG^+Env^{++}$) were sorted into individual wells of a 96-well PCR plate containing 20 uL of lysis buffer containing 0.5 uL RNAse OUT (40 U/uL, Invitrogen 10777019), 5 uL 5X First Strand Buffer (Invitrogen 18080044), 1.25 uL DTT (0.1 M, Invitrogen 18080044), 0.0625 uL IGEPAL CA-630 detergent (Sigma I8896), and 13.25 uL nuclease-free water. Cell lysate was centrifuged at 300 x g for 5 min at 4°C and stored at -20°C. Bone marrow plasma cells were sorted in a similar manner, with the following changes. After viability staining cells were washed, resuspended in PBS supplemented with 5% FBS and 1:20 anti-FcR (Invitrogen 14-9156-42), and incubated for 15 min at 4°C. Without washing, cells were then stained with a cocktail of antibodies against CD3, CD8a, CD14, CD16, CD20, CD102 (clone CBR-IC2/2, Biolegend 328506), and CD31 (clone C31.7, Novus NBP2-33136AF647). Plasma cells ($CD3^-CD8a^-CD14^-CD16^-CD20^-CD102^+CD31^+$) were sorted into 20 uL of RPMI supplemented with 10% FBS and 100 U/mL penicillin-streptomycin, counted, and immediately carried forward for single-cell analysis using the 10X Genomics platform.

Single-cell BCR sequencing from PBMCs

Immunoglobulin heavy (IgH) and light (IgK, IgL) chain genes were amplified as previously described (*39*). Briefly, single B cell RNA was reverse transcribed by adding 2.3 uL random hexamers (200 ng/uL, Thermo SO142), 2 uL dNTP mix (10 mM per nucleotide, Invitrogen 18427088), 1 uL Superscript III reverse transcriptase (200 U/uL, Invitrogen 18080044), and 0.7

uL nuclease-free water and incubating in a thermocycler at 42°C for 10 min, 25°C for 10 min, 50°C for 1 hr, and then 94°C for 5 min. IgH, IgK, and IgL genes were amplified by two rounds of nested PCR, each using 3 uL of template, 5 uL 10X buffer (Qiagen 203207), 1uL dNTP mix (Qiagen 201901), 0.5 uL $MgCl_2$ (Qiagen 203207), 0.5 uL carrier RNA (Qiagen 1017647), 0.75 uL forward primer mix (50 uM per primer), 0.5 uL of each reverse primer (25 uM), 0.4 uL HotStart Taq polymerase (100 mM per nucleotide, Qiagen 203207), and 39.7 uL nuclease-free water. PCR conditions were as follows: 95°C for 15 min, then 50 cycles of 95°C for 30 sec, 51°C (Round 1) or 58°C (Round 2) for 30 sec, 72°C for 30 sec, with a final extension of 72°C for 7 min. Amplicons were visualized by agarose gel electrophoresis, Sanger sequenced (Genewiz), and analyzed computationally using IMGT V-QUEST (*118*).

Single-cell BCR sequencing from bone marrow-derived plasma cells

$CD102^+CD31^+$ bone marrow cells suspension was loaded on a Chromium X instrument (10X Genomics) to generate a single-cell bead emulsion, at a loading concentration for a target recovery of 20,000 cells per reaction. Single-cell RNA-seq libraries were then prepared using Chromium Next GEM Single Cell 5' Kit v3 bead and library construction kit (10X Genomics). BCR libraries were constructed using Chromium Single Cell Human BCR Amplification Kit (10X Genomics) and macaque-specific primers targeting the constant regions of heavy chain IgM, IgG, and IgA gene isotypes and light chain IgK and IgL genes, as described (*119*). BCR libraries were indexed using Dual Index Kit TT Set A kit (10X Genomics) and sequenced on an Illumina NextSeq 2000 instrument at a minimum read depth of 5000 reads/cell. Sequencing reads were analyzed using Cell Ranger v7.2 (10X Genomics) and heavy chain BCR sequences were recovered from a total of 21,220 cells. AM12-352 lineage members were identified based on CDRH3 length and amino acid sequence homology, heavy chain V, D and J gene usage, and light chain V and J gene usage.

Antibody cloning and expression

Immunoglobulin heavy and light chain variable regions of interest were synthesized and cloned (Genscript) into rhesus IgG1, IgK, or IgL expression vectors immediately upstream of the constant region using AgeI/NheI, AgeI/BsiWI, or AgeI/ScaI restriction sites, respectively, as previously described (*39*). Recombinant mAbs were expressed by co-transfecting paired heavy and light chain plasmids into 293F cells at a 1:2 ratio using ExpiFectamine transfection reagent (Gibco A14525), purified using rProtein A/Protein G Sepharose Gravitrap kit (Cytiva 28985256), concentrated in 30 kDa centrifugal filters (Sigma UFC903024), and buffer exchanged into PBS. Antibody concentration was measured fluorometrically using a Qubit Protein Assay kit (Invitrogen Q33212).

Bulk BCR sequencing and lineage tracing

Immunoglobulin heavy and light chain sequences were amplified and analyzed from bulk IgM^+IgD^+ or IgG^+ B cells as previously described (*48, 87, 120*). Briefly, RNA was extracted using RNAzol RT (Molecular Research Center RN190) according to the manufacturer's instructions. cDNA was synthesized using 5' rapid amplification of cDNA ends (5' RACE) with SMARTer template switching and SuperScript II reverse transcriptase (Invitrogen 18064071) and purified using AMPure XP beads (Beckman Coulter A63881). BCR library construction was performed as previously described (*48, 120*) using KAPA HiFi HotStart ReadyMix PCR Kit (Roche KK2601) and primers annealing in the IgG, IgK, or IgL constant region. Additional PCR steps were used to append barcodes and Illumina P5 and P7 sequencing adapters. All libraries were sequenced on an Illumina MiSeq sequencer (with 2 × 300 bp runs using the 600-cycle MiSeq Reagent V3 kit) or

NextSeq 2000 sequencer (with P1 or P2 600-cycle kits). Sequences from the naïve IgM+IgD+ B cell population were used to construct an individualized germline immunoglobulin repertoire for each macaque of interest using IgDiscover (*57, 121*) and MINING-D (*122*) computational pipelines, as previously described (*48*). Sequences from the IgG+ B cell population were used to trace lineages of interest and infer unmutated common ancestors using the SONAR computational pipeline (*58*), as previously described (*48*).

Identification of homologous human V gene segment alleles

Homologous human V gene segment alleles were found using global alignments between the rhesus UCA V gene segment alleles and the orgdb database (https://ogrdb.airr-community.org/germline_sets/Human release data 2024-10-12) after removal of the novel alleles (designated with i) with BLOSUM62 scoring and gap opening and extension penalties of 11 and 1, respectively (*60*). The best human V gene segment allele was identified based on protein identity, and ties were broken using biochemical similarity.

Viral RNA sequencing

The SHIV *rev-vpu-env* cassette was sequenced using single-genome sequencing (SGS) approach as previously described (*61, 62*). Briefly, reverse transcription of RNA was performed by using reverse transcriptase (Superscript III) and reverse primer SIVmac251.R.R1 5′-CACTAGCTTACTTCTAAAATGGCAGC-3′ (nt 10,138–10163, SIVmac239) according to the manufacturer's instructions. The *rev-vpu-env* cassette was amplified by nested PCR with the following primers: first round forward primer SHIV.RevEnv.F1 5′-CGAAAGGCTTAGGCATCTCCTATG-3′ (nt 5949 - 5972, HXB2), second round forward primer SHIV.RevEnv.F2 5′-TTAGGCATCTCCTATGGCAGGAAGA-3′ (nt 5957-5981, HXB2), first round reverse primer SIVmac251.R.R1 5′-CACTAGCTTACTTCTAAAATGGCAGC-3′ (nt 10,138 - 10,163, SIVmac239), and second reverse primer SIVmac251.R.R2 5′-TACTTCTAAAATGGCAGCTTTATTGAAGAGG-3′ (nt 10,125 - 10,155, SIVmac239). A total of 8406 *env* gp140 sequences were aligned using the MUSCLE alignment method and manually inspected using Geneious Prime to improve the alignment results based on codon translation. The sequence alignment was then analyzed using Pixel plot (https://www.hiv.lanl.gov/content/sequence/pixel/pixel.html) and the LASSIE program (*80*).

Cryo-EM sample preparation

Purified Fabs and SOSIP Envs were incubated at a Fab:Env protomer molar ratio of ~4:1 at room temperature (for mature bNAb Fabs) or a ratio of ~1.1:1 at 37˚C (for V634-136 UCA Fab). Resulting mature Fab-Env complexes were purified by SEC using a Superdex 200 Increase 10/300 GL analytical column (GE Healthcare Life Sciences) in TBS pH 8.0, and fractions corresponding to a Fab-Env complex were concentrated to a final concentration of 2-3 mg/mL using a 50 kDa molecular weight cutoff spin concentrator (Millipore). The V634-136 UCA Fab-Env complex was not subjected to SEC purification.

Immediately prior to grid preparation, a fluorinated octyl maltoside solution (Anatrace) was added to each sample from a 0.5% (w/v) stock to a final concentration of 0.02% (v/v) as described (*123*), and 3 µL of each complex was applied to freshly glow-discharged Quantifoil R1.2/1.3 grids (300 mesh Cu; Electron Microscopy Sciences) that had been treated for 1 minute at 20 mA using a PELCO easiGlow device (Ted Pella). Grids were plunge-frozen using a Vitrobot Mark IV (Thermo Fisher) at 22°C and 100% humidity. Blotting was performed with Whatman

No. 1 filter paper for 3 s using a blot force of 0. Samples were vitrified by rapid plunging into liquid ethane cooled by liquid nitrogen.

Cryo-EM data collection

Single-particle cryo-EM data acquisition was performed on two transmission cryo-electron microscopes: A Titan Krios (Thermo Fisher) operating at 300 kV was used for imaging the AJ09-21, AJ09-83, and AJ09-110 Fabs in complex a SOSIP Env, and a Talos Arctica (Thermo Fisher) operating at 200 kV was used for the remaining Fab-Env complexes involving the AM12-340, AM12-347, AM12-351, AM12-352, NN39-25, V634-136, V634-136 UCA, V645-158, NN39-25, and NN39-171 Fabs. Automated data collection was carried out using SerialEM software (*124*), employing beam-image shift across a 3×3 grid of 1.2 µm holes with one exposure per hole. For the AJ09-21, AJ09-83 or AJ09-110–Env datasets recorded on the Krios, 40-frame movies were captured in super-resolution mode using a Gatan K3 camera positioned behind a BioQuantum energy filter (Gatan) with a 20 eV slit and a pixel size of 0.416 Å (105,000x magnification). For datasets collected on the Talos Arctica, 40-frame movies were captured in a super-resolution mode using Gatan K3 camera with a pixel size of 0.465Å (45,000x magnification). Data processing was carried out for all datasets using cryoSPARC v.4 (*125*). Data collection parameters are summarized in **Table S2**.

Cryo-EM data processing

Briefly, cryo-EM movies were patch motion corrected in cryoSPARC v4 (*125*) to account for beam-induced motion, including dose weighting, following the binning of super-resolution frames. For CTF parameter estimations, non-dose-weighted micrographs were processed using the Patch CTF job in cryoSPARC. Micrographs displaying poor CTF fits or evidence of crystalline ice in their power spectra were excluded from further analysis. Particle picking was performed in cryoSPARC v4 (*125*) using Blob picker (minimum particle diameter = 120 Å, maximum particle diameter = 180 Å) for reference-free selection. Particle extraction was carried out using a box size of 360 Å. Particles were subjected to several rounds of 2D classification. The best class averages representing different views were used to generate ab initio models, which were further refined using homogenous refinement (with minimize over per-particle scale) or non-uniform refinement by applying C1 or C3 symmetry. The particles and 3D volumes from homogenous refinement jobs combined with micrographs from the CTF estimation for reference-based motion correction before further refining using homogenous refinement or non-uniform refinement to generate the final refined 3D volume for all structures.

Cryo-EM structure modelling and refinement

To generate initial coordinates of the complex, individual chains from reference structures of the fully glycosylated BG505 SOSIP.664 HIV-1 Env trimer (PDB: 5T3X or 5T3Z) were independently docked into cryo-EM density maps using UCSF ChimeraX (*102*). Preliminary models were subjected to rigid-body fitting and subsequently refined in real space against the EM maps. Manual adjustments and sequence corrections were performed using Coot v.0.8.9 (*126*), followed by iterative cycles of model rebuilding in Coot and refinement using Phenix (*127*). N-linked glycans were incorporated at predicted glycosylation sites by interpreting low-resolution ("blurred") maps filtered with varying B-factors. Final models were assessed for stereochemical quality using MolProbity (*128*).

Structural analyses
Structure figures were created using ChimeraX (*102*) and PyMOL (*129*). To highlight Fab densities within EM reconstructions, maps were segmented, with each region visualized in a unique color. The gp41 region was rendered using a color scheme that corresponded to the fitted SOSIP coordinates from lower-resolution reconstructions. Estimations of local resolution were performed using cryoSPARC v4 (*125*). To quantify interfacial interactions, buried surface areas were analyzed via PDBePISA v1.48.65 (*130*), employing a 1.4 Å solvent probe radius. Hydrogen bonds were defined as interactions involving distances below 4.0 Å and an angle between donor, hydrogen, and acceptor atoms greater than 90°. Van der Waals contacts were considered when interatomic distances were under 4.0 Å. These interaction assessments are considered approximate due to limitations in resolution. Structural comparisons, including root-mean-square deviation (r.m.s.d.) values from pairwise Cα alignments, were calculated in PyMOL (*129*) without applying outlier exclusion. Criteria used to define epitope regions are provided in the respective figure legends.

The angles of approach of V3 Abs were calculated as described (*31*). The Env trimer 3-fold symmetry axis was aligned on the z axis, with the center of mass of one monomer's $N332_{gp120}$ epitope placed on the x axis. The $N332_{gp120}$ epitope was defined by residues $G324_{gp120}$, $D325_{gp120}$, $V326_{gp120}$, $R327_{gp120}$, $M328_{gp120}$, $A329_{gp120}$, $H330_{gp120}$, $I415_{gp120}$, $L416_{gp120}$, and $P417_{gp120}$. The center of mass of a gp41 residue ($L587_{gp41}$ in all three protomers) was aligned to the negative z axis. The latitudinal angle was that formed by the z axis and a vector from the $N332_{gp120}$ epitope center of mass to the HC center of mass (carbon-a atoms for six b-strands; HC residues 21-24, 34-39, 46-52, 67-71, 77-82, and 89-92) in the x-z plane. The longitudinal angle was that formed by the x axis and the $N332_{gp120}$ epitope-HC vector in the x-y plane. The HC-LC twist angle was the angle between the x axis and a vector from the LC center of mass (carbon-a atoms for six b-strands, LC residues 18-24, 34-37, 45-48, 62-66, 70-76, and 84-88) to the HC center of mass in the x-y plane.

Site-specific glycan analysis
100 μg aliquots of each sample were denatured for 1h in 50 mM Tris/HCl, pH 8.0 containing 6 M of urea and 5 mM dithiothreitol (DTT). Next, Env samples were reduced and alkylated by adding 20 mM iodoacetamide (IAA) and incubated for 1h in the dark, followed by a 1h incubation with 20 mM DTT to eliminate residual IAA. The alkylated Env samples were buffer exchanged into 50 mM Tris/HCl, pH 8.0 using Vivaspin columns (10 kDa) and two of the aliquots were digested separately overnight using Trypsin (Mass Spectrometry Grade, Promega), chymotrypsin (Promega), or alpha-lytic protease (Sigma Aldrich) at a ratio of 1:30 (w/w). The next day, the peptides were dried and extracted using an Oasis HLB μElution Plate (Waters).

The peptides were dried again, re-suspended in 0.1% formic acid, and analyzed by nanoLC-ESI MS with a Vanquish Neo (Thermo Fisher Scientific) system coupled to an Orbitrap Eclipse Tribrid mass spectrometer (Thermo Fisher Scientific) using stepped higher energy collision-induced dissociation (HCD) fragmentation. Peptides were separated using a μPAC Neo HPLC Column (180 μm × 110 cm). A trapping column (PepMap 100 C18 3μM 75μM × 2cm) was used in line with the LC prior to separation with the analytical column. The LC conditions were as follows: 280-minute linear gradient consisting of 4-32% acetonitrile in 0.1% formic acid over 260 minutes followed by 20 minutes of alternating 76% acetonitrile in 0.1% formic acid and 4% ACN in 0.1% formic acid, used to ensure all the sample had eluted from the column. The flow rate was set to 300 nL/min. The spray voltage was set to 2.5 kV and the temperature of the heated

capillary was set to 55 °C. The ion transfer tube temperature was set to 275 °C. The scan range was 350−2000 m/z. The stepped HCD collision energies were set to 15, 25 and 45% and the MS2 for each energy was combined. Precursor and fragment detection was performed using an Orbitrap at a resolution MS^1 = 120,000, MS^2 = 30,000. A standard AGC target for MS^1 ($4e^5$) and MS^2 ($1e^4$) and auto injection times (MS^1 =50ms MS^2 =54ms) were used.

Glycopeptide fragmentation data were extracted from the raw file using Byos (Version 5.5; Protein Metrics Inc.). The glycopeptide fragmentation data were evaluated manually for each glycopeptide; the peptide was scored as true-positive when the correct b and y fragment ions were observed along with oxonium ions corresponding to the glycan identified. The MS data was searched using the Protein Metrics 305 N-glycan library. The relative amounts of each glycan at each site as well as the unoccupied proportion were determined by comparing the extracted chromatographic areas for different glycotypes with an identical peptide sequence. All charge states for a single glycopeptide were summed. The precursor mass tolerance was set at 4 ppm and 10 ppm for fragments. A 1% false discovery rate (FDR) was applied. The relative amounts of each glycan at each site as well as the unoccupied proportion were determined by comparing the extracted ion chromatographic areas for different glycopeptides with an identical peptide sequence. Glycans were categorized according to the composition detected.

HexNAc(2)Hex(9−3) was classified as M9 to M3. Any of these structures containing a fucose were categorized as FM (fucosylated mannose). Complex-type glycans were classified according to the number of HexNAc subunits and the presence or absence of fucose. Core glycans refer to truncated structures smaller than M3. As this fragmentation method does not provide linkage information, compositional isomers are grouped.

To obtain data for sites that frequently present low intensity glycopeptides, the glycans present on the glycopeptides were homogenized to boost the intensity of these peptides. This analysis loses fine processing information but enables the ratio of high mannose: complex: unoccupied to be determined. The remaining glycopeptides were first digested with Endo H (New England Biolabs) to deplete oligomannose- and hybrid-type glycans and leave a single GlcNAc or GlcNAcFuc residue at the corresponding site. The reaction mixture was then dried completely and resuspended in a mixture containing 50 mM ammonium bicarbonate and PNGase F (New England Biolabs) using only H_2O^{18} (Sigma-Aldrich) throughout. This second reaction cleaves the remaining complex-type glycans but leaves the GlcNAc residues remaining after Endo H cleavage intact. The use of H_2O^{18} in this reaction enables complex glycan sites to be differentiated from unoccupied glycan sites as the hydrolysis of the glycosidic bond by PNGaseF leaves a heavy oxygen isotope on the resulting aspartic acid residue. The resultant peptides were purified as outlined above and subjected to reverse-phase (RP) nanoLC-MS. Instead of the extensive N-glycan library used above, three modifications were searched for: +203 Da corresponding to a single GlcNAc, or +349 corresponding to a GlcNAcFuc, a remnant of an oligomannose/hybrid glycan (+/- fucose), and +3 Da corresponding to the O^{18} deamidation product of a complex glycan. Data acquisition and analysis was performed as above and the relative amounts of each glycoform were determined, including unoccupied peptides.

Longitudinal Env evolution analyses

Our previously established algorithm LASSIE was used to identify Env sites under natural selection in each RM using the threshold of ≥80% TF loss at any timepoint (*80*), as was done previously for SHIV-infected macaques (*87*). To identify selected Env sites significantly enriched in SHIV.5MUT-infected macaques that developed bNAbs, the contingency tables of number of

macaques that developed or failed to develop bNAbs with or without a site under selection were subjected to Fisher's exact tests and all significant associations (with uncorrected $p < 0.05$) were identified. All significantly enriched sites also met Benjamini-Hochberg multiple test correction with false discovery rate (FDR) < 20%. Hypervariable V1 loop characteristics of length and number of glycans were also tested for statistical enrichment using maximum increase in each characteristic's timepoint average over that of TF for each macaque. For calculation of hypervariable V1 loop number of PNGS for a given sequence, if two sequons were separated by a Pro (e.g. NNTPNAT), only one was assumed to be glycosylated based on 5MUT site-specific glycan analyses (**Fig. 4E**). Mutations at LASSIE selected sites and V3 loop sites were visualized using the Python software Logomaker (*131*).

Env-bNAb coevolution analysis

To assess whether plasma recognition of heterologous viruses required quasispecies mutation away from the infecting SHIV.5MUT (termed transmitted founder or TF virus) at key sites, we determined how often mutations emerged in the viral quasispecies at these sites prior to or contemporaneously with the earliest observed neutralization of each heterologous virus. Of the ten key sites identified as being preferentially selected in macaques that developed bNAbs (15, 87, 308, 325, 330, 332, 343, 359, 363, and 440), we determined the subset relevant to recognition of a particular heterologous virus on a per-animal basis. We assumed that the bNAb lineage in a given animal would select the most relevant resistance sites, and thus the subsets of sites differ between animals. For each heterologous virus in our screening panel, we only analyzed sites that differed between that virus and SHIV.5MUT, assuming that variation at those sites must be acquired before the bNAb lineage can recognize that virus. Additionally, sites 15 and 343 were not considered to be relevant to recognition of viruses encoding M-group consensus residues Q15 or Q343, as these residues were never sampled by the quasispecies in any SHIV.5MUT-infected macaque that developed bNAbs and thus are unlikely to be associated with resistance.

We determined the earliest timepoint at which mutations were observed at each of the relevant key sites in the evolving quasispecies of a given animal, even if the mutant was rare at that timepoint. While the mutant residue often matched that carried by the heterologous virus, we interpreted any variant residue as potentially contributing to recognition. This is particularly pertinent for position 363, which is a PNGS in SHIV.5MUT but is highly variable in the heterologous virus panel. The loss of this glycan in the quasispecies, which can be achieved by mutation to any non-asparagine residue at 363, is likely more important than the particular residue sampled. Similarly, while the heterologous viruses exhibit high variability at position 440, none of them carry an arginine. Nevertheless, Q440R was by far the most common escape mutation observed in the quasispecies of SHIV.5MUT-infected macaques that developed bNAbs, suggesting that the loss of the TF glutamine was more important than accommodation of a particular residue at this site. An example is shown in **Fig. 6A**, where nine of the ten key sites are under selection in animal V634. Only six of these differ between SHIV.5MUT and T250, including Q15 and Q343 **(Fig. 7A)**. So only the remaining 4 sites (325, 330, 363, and 440) were considered to be relevant for the bNAb lineage in this animal to recognize T250 **(Fig. 7B)**. Consistent with our hypothesis, mutations emerged at all 4 of these sites before or at the timepoint at which plasma neutralization of T250 was first detected. Indeed, in animal V634 this was the case for 20 of 21 residues across all heterologous viruses and relevant selected sites. The one exception was for site 440 and recognition of 25710, where the plasma acquired neutralization activity against 25710 at week 20 even though mutations at site 440 were not detected until week 24. A summary of all

results is shown in **Fig. 7C-D**, and the individual analyses for each macaque are shown in **fig. S18-31**.

Given that increased V1h length and number of glycans were also significantly enriched in the quasispecies of SHIV.5MUT-infected macaques that developed bNAbs, we queried their contribution to the acquisition of plasma neutralization breadth in each macaque. V1h elongation was considered a key relevant feature for recognition of six of eight heterologous viruses that had longer V1h regions than SHIV.5MUT (>13 AA) **(Fig. 7A)**. In addition, all eight heterologous viruses had more V1h glycans than SHIV.5MUT (>1 glycan) **(Fig. 7A)**, and so an increased number of V1h PNGS was considered a relevant key feature for all viruses. In our analyses, we thus assumed that any increase in these parameters selected for antibodies capable of tolerating longer, more-glycosylated V1h regions. Indeed, the number of V1h glycans began to increase early in most SHIV.5MUT-infected macaques, and variants with >1 PNGS were observed in the quasispecies of animal V634 by week 4 post-infection **(Fig. 7B)**. V1h elongation was observed in the quasispecies at week 32, contemporaneous with plasma neutralization of 2/6 of the heterologous viruses for which V1h length is relevant (CAP256SU and BJOX02000). While there was low-titer neutralization of the other 4 viruses (T250, Ce1176, TRO11, and CH119) prior to this timepoint, we observed a substantial increase in potency upon emergence of the V1-elongated variants, suggesting that these variants may have selected for bNAb lineage members with enhanced affinity.

Statistical analyses

All statistical tests were calculated in GraphPad Prism 10 (version 10.4.2) or using the Stats module from SciPy (version 0.18.0) (*132*).

Supplementary Text

Immunogen design

To retain immunogen Envs in their prefusion conformation, we stabilized protein and mRNA constructs using MD39 mutations (*24*) and introduced potential N-linked glycosylation sites (PNGS) at gp120 positions 230, 241, and 344 (“3fill”) to shield immunodominant off-target epitopes (*27, 69, 70*). Envs expressed by mRNA constructs were further engineered to include an HLA-DR signal peptide (*133*), a flexible $(GGGGS)_2$ linker in place of the furin cleavage site (*134*), two helix-breaking prolines (*135*), and a truncated SIVmac cytoplasmic tail with an endocytosis knockout mutation (*136*) **(fig. S1A)**. Both RC1-3fill and 11MUTB-3fill mRNA constructs expressed well on the surface of 293F cells and exhibited favorable antigenic profiles **(fig. S1B)**. The protein immunogens were multimerized on mi3 nanoparticles (*99, 104*) and delivered with saponin/MPLA nanoparticle (SMNP) (*137*) adjuvant as either a bolus (n=4) or an escalating dose regimen over the course of two weeks (n=4) (*103, 138*). The mRNA immunogens were encapsulated in lipid nanoparticles (LNPs) and delivered as a bolus.

A

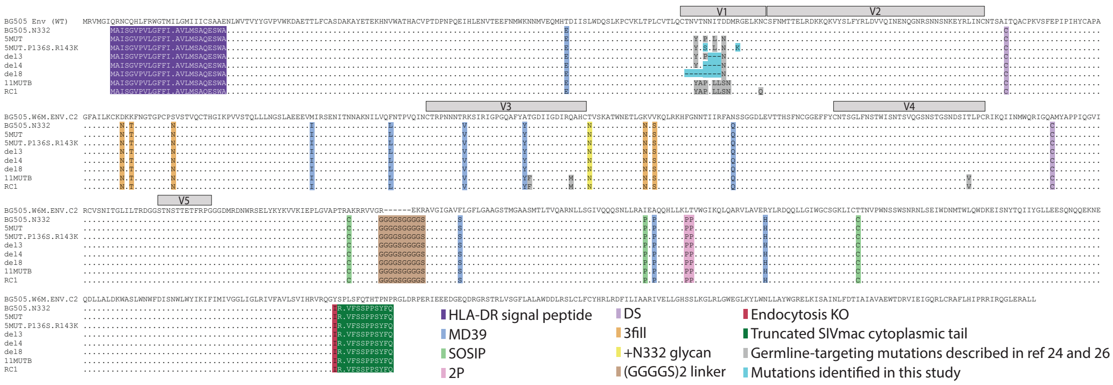

B

MFI Fold Change over Mock (0-3, 3-10, 10-100, 100-1000, >1000)

Class	Epitope	mAb ID	BG505	P136S	5MUT	del3	del4	del8	RC1	11MUTB
bNAb	V2	PGDM1400	384.9	340.8	504.3	474.5	349.2	304.0	129.9	69.55
		PGT145	416.3	404.3	517.0	488.8	418.3	255.3	151.4	66.99
	V3	DH270.6	614.2	799.1	1212.2	812.8	822.8	726.7	224.3	135.3
		BG18	463.6	390.0	574.5	469.3	382.5	339.4	118.0	66.84
		2G12	541.5	515.8	556.2	556.2	397.4	517.0	184.5	102.7
	CD4bs	N6	335.9	410.1	467.8	402.2	253.9	370.3	112.4	62.32
		VRC01	422.9	502.7	607.8	882.4	513.8	550.1	152.6	75.94
		IOMA	328.8	333.6	661.3	654.9	462.3	424.5	98.98	42.27
	MPER	10E8	131.1	104.6	213.0	247.1	127.0	130.4	43.19	35.80
nnAb	CD4i	17b	1.91	1.11	1.53	1.58	1.80	1.50	1.01	1.04
	V2i	830A	6.62	6.56	9.96	7.52	5.40	5.98	2.18	1.78
		697	1.59	2.12	3.64	2.02	1.49	1.48	1.30	1.07
		1361	6.25	2.32	2.49	2.15	1.21	1.35	1.06	0.98
		1393A	1.70	1.07	1.16	1.21	1.07	1.22	0.96	0.90
	Linear V3	3074-GB	247.8	201.6	514.0	574.9	279.1	283.2	113.2	136.4
		3791	210.1	191.6	412.1	495.9	154.0	231.0	81.47	100.8
Negative control		anti-SARS2	1.65	0.86	1.86	1.03	0.88	0.87	0.90	0.83

C

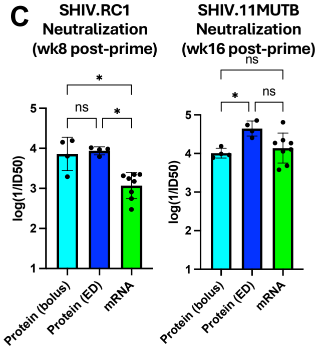

Group		RM ID	Weeks post-prime	MLV	RC1	11MUTB	5MUT	BG505.N332
1	Bolus	V630	16	<20	21395	7806	<20	<20
		V631	16	<20	18379	9747	<20	<20
		V632	16	<20	17908	15370	<20	<20
		V633	16	<20	13472	9124	<20	<20
	Escalating-dose	V634	16	<20	50176	38124	<20	<20
		V635	16	<20	95238	54645	<20	<20
		V636	16	<20	121403	71788	<20	<20
		V637	16	<20	36900	25471	<20	<20
2	mRNA	V638	16	<20	64309	64267	<20	<20
		V639	16	<20	7553	4789	<20	<20
		V640	16	<20	22148	18762	<20	<20
		V641	16	<20	14023	11362	<20	<20
		V642	16	<20	31606	22080	<20	<20
		V643	16	<20	21079	12721	<20	<20
		V644	16	<20	30340	18536	<20	<20
		V645	16	<20	7962	3797	<20	<20

D

Class	Epitope	mAb ID	SHIV.BG505.N332	SHIV.5MUT
nnAb	Linear V3	3074	>250	136
		3791	>250	>250
		19b	>250	>250
	V2p	CAP228-16H	>250	>250
		CAP228-3D	>250	>250
	V2i	1393A	>250	>250
		830A	>250	>250
		697-D	>250	>250
		1361	>250	>250
	CD4i	17b-PDB	>250	>250
		A32	>250	>250
bNAb	V3	PGT121	0.03	0.007
		PGT128	0.03	0.01
		DH270.6	0.57	0.08
	V2	PGDM1400	0.001	0.001
		PGT145	0.04	0.16
	CD4bs	VRC01	0.15	0.28
		IOMA	1.80	4.64

E

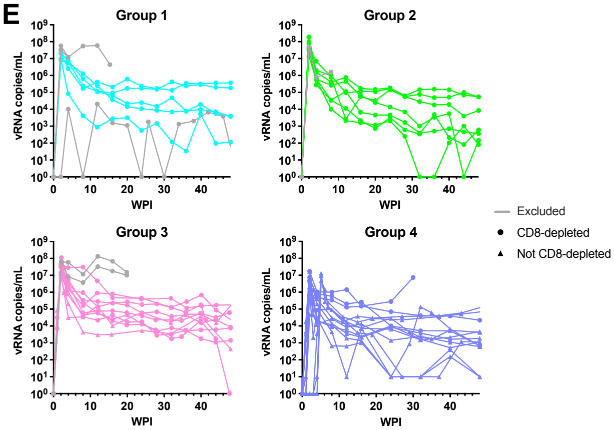

Fig. S1. RC1 and 11MUTB immunization induces robust autologous neutralizing responses. **(A)** mRNA constructs used in this study. Amino acid sequences are aligned to the BG505 wildtype Env sequence (dots indicate identity and dashes indicate deletions). Stabilization, glycan filling, and germline-targeting mutations are indicated. The RC1 and 11MUTB mRNA constructs were used as immunogens in this study **(Fig. 1A)**. **(B)** Antigenicity of all mRNA-expressed, stabilized, membrane-bound Envs shown in **(A)**. mRNA was transfected into 293F cells and expressed Envs were tested by flow cytometric analysis for binding to a panel of bNAbs (top) as well as conformation-sensitive non-neutralizing antibodies (nnAb, bottom). Binding was quantified as fold-change in mean fluorescence intensity (MFI) over mock-transfected cells, and data points with a >3-fold increase are colored as indicated. **(C)** Plasma neutralization of the indicated virus at 8 and 16 weeks post RC1 immunization, represented as log(1/ID_{50}) (left) or reciprocal ID_{50}

(right). Protein was given as bolus or escalating dose (ED). Kruskal-Wallis test with Dunn's correction for multiple comparisons. Not significant (ns) $P > 0.05$; $^{*}P < 0.05$. **(D)** Antigenicity of SHIV.5MUT and SHIV.BG505.N332. Neutralization sensitivity was tested using a panel of conformation-sensitive non-neutralizing antibodies (nnAb) and broadly neutralizing antibodies (bNAb). Titers are expressed as IC_{50} in µg/mL. **(E)** Longitudinal viral load in macaques infected with SHIV.5MUT (Groups 1-3) or SHIV.BG505.N332 (Group 4), represented as vRNA copies/mL. Circles indicate animals that were CD8-depleted 2-3 days prior to infection, and triangles indicate animals that were not. Grey lines indicate animals that were excluded from subsequent analysis due to rapid progression to AIDS or abnormally low viral load. WPI, weeks post-infection.

					Tier-2 Autologous				Tier-2 Heterologous							
Group	RM ID	Weeks post-infection	Weeks post-imm	MLV	5MUT A1	5MUT. S334A A1	5MUT. D325N A1	BG505 A1	T250 02_AG	CAP256 SU C	Q23 A1	25710 C	BJOX02000 07_BC	Ce1176 C	TRO11 B	CH119 07_BC
1	V631	20	36	<20	898	1,908	894	<20	<20	<20	<20	<20	<20	<20	<20	<20
		32	48	<20	505	1,273	1,068	90	<20	<20	<20	<20	<20	<20	<20	<20
		40	56	<20	879	1,247	362	101	<20	<20	<20	<20	<20	<20	<20	<20
		44	60	<20	903	1,016	464	158	<20	<20	<20	<20	<20	<20	<20	<20
		48	64	<20	639	933	369	40	<20	<20	<20	<20	<20	<20	<20	<20
	V632	20	36	<20	1,267	1,637	772	134	<20	<20	<20	<20	<20	<20	<20	<20
		32	48	<20	1,844	685	2,247	646	<20	<20	<20	<20	<20	<20	<20	<20
		40	56	<20	3,666	895	2,279	216	<20	<20	<20	<20	<20	<20	<20	<20
		44	60	<20	1,834	511	1,300	304	<20	<20	<20	<20	<20	<20	<20	<20
		48	64	<20	2,838	265	1,814	78	<20	<20	<20	<20	<20	<20	<20	<20
	V633	20	36	<20	5,750	2,366	5,133	450	<20	<20	<20	48	<20	<20	<20	<20
		32	48	<20	3,077	1,187	2,692	691	24	37	368	225	28	43	85	100
		40	56	<20	2,677	1,380	2,562	416	85	32	163	99	25	<20	32	55
		44	60	<20	2,723	1,342	1,443	567	82	23	230	127	34	<20	43	100
		48	64	<20	1,998	1,032	1,148	568	91	<20	244	37	53	<20	<20	<20
		65	81	<20	657	572	494	298	33	20	163	<20	28	<20	<20	<20
		74	90	<20	495	461	392	225	26	<20	298	<20	37	<20	<20	<20
	V634	20	36	<20	6,406	8,525	2,630	919	<20	<20	<20	33	<20	<20	<20	<20
		24	40	<20	2,658	4,591	1,707	663	29	<20	99	38	<20	<20	<20	<20
		28	44	<20	3,828	5,063	2,008	670	97	<20	326	277	<20	50	138	54
		32	48	<20	4,092	8,000	3,224	1,724	239	22	887	629	69	98	422	93
		36	52	<20	2,995	5,621	2,572	1,393	407	66	590	431	108	204	628	120
		40	56	<20	6,402	7,102	3,575	2,459	542	64	1,241	743	137	202	1,076	160
		44	60	<20	4,914	3,614	2,928	1,109	783	42	1,590	1,117	193	238	803	122
		48	64	<20	5,780	5,107	3,855	1,631	1,092	29	2,094	1,110	142	291	913	278
		65	81	<20	953	5,522	1,081	861	491	27	435	105	70	40	183	40
		74	90	<20	952	3,894	1,054	573	209	<20	265	92	47	24	91	35
	V635	20	36	<20	6,064	10,416	2,698	657	<20	<20	<20	<20	<20	<20	<20	<20
		24	40	<20	2,340	6,042	1,576	478	<20	<20	<20	<20	<20	<20	<20	<20
		28	44	<20	6,472	12,101	853	365	<20	<20	36	<20	<20	<20	<20	<20
		32	48	<20	7,364	10,476	1,285	585	<20	22	135	<20	20	<20	<20	<20
		40	56	<20	7,042	7,593	3,328	428	<20	120	1,225	<20	474	58	128	116
		44	60	<20	7,593	5,263	1,716	538	<20	324	1,981	45	878	93	198	260
		48	64	<20	4,202	6,658	3,229	870	92	297	1,689	48	498	117	181	252
		65	81	<20	6,519	3,815	1,410	723	104	211	700	62	338	35	112	71
		74	90	<20	2,465	3,914	1,825	391	41	130	497	44	577	56	105	125
2	V638	20	36	<20	2,275	3,441	1,826	546	<20	<20	<20	<20	<20	<20	<20	<20
		32	48	<20	2,350	1,601	1,923	815	<20	<20	<20	<20	<20	<20	<20	<20
		40	56	<20	1,300	343	2,513	354	<20	<20	<20	<20	<20	31	<20	<20
		44	60	<20	1,843	531	1,754	406	<20	<20	<20	<20	<20	52	<20	<20
		52	68	<20	470	426	366	34	<20	<20	<20	<20	<20	<20	<20	<20
		65	81	<20	754	420	292	60	<20	<20	<20	<20	<20	<20	<20	<20
		74	90	<20	293	349	228	70	24	<20	<20	33	<20	<20	<20	<20
	V639	20	36	<20	2,900	5,501	1,984	455	30	<20	33	<20	<20	<20	<20	<20
		32	48	<20	6,993	8,354	4,088	1,326	34	46	127	<20	<20	<20	<20	<20
		40	56	<20	3,765	5,467	6,075	852	64	<20	97	<20	<20	<20	<20	<20
		44	60	<20	3,220	3,885	3,101	251	71	132	402	<20	<20	<20	<20	<20
		52	68	<20	2,896	4,490	4,082	249	20	<20	126	<20	<20	<20	<20	<20
		56	72	<20	3,514	6,684	5,345	978	<20	<20	233	<20	<20	<20	<20	<20
		65	81	<20	2,160	3,617	1,735	701	50	<20	591	<20	26	<20	<20	<20
		74	90	<20	2,459	4,831	1,421	463	30	<20	251	23	26	<20	<20	<20
	V640	20	36	<20	5,510	7,570	4,023	1,455	<20	<20	<20	<20	<20	<20	<20	<20
		32	48	<20	5,615	3,808	3,908	1,220	<20	<20	<20	<20	<20	<20	<20	<20
		40	56	<20	4,230	2,068	4,671	986	<20	<20	75	<20	<20	26	40	<20
		44	60	<20	4,496	2,067	2,543	421	<20	<20	41	<20	<20	<20	<20	<20
		52	68	<20	2,367	953	2,088	174	<20	<20	45	<20	<20	<20	<20	<20
		65	81	<20	1,087	954	854	446	<20	<20	95	<20	<20	<20	<20	<20
		74	90	<20	1,001	789	552	195	<20	<20	50	<20	<20	<20	<20	<20
	V641	20	36	<20	2,416	3,478	4,202	128	<20	<20	<20	<20	<20	<20	<20	<20
		24	40	<20	1,754	6,329	3,499	110	30	<20	<20	26	<20	<20	<20	<20
		28	44	<20	3,319	8,150	3,471	191	81	23	63	56	<20	24	28	<20
		32	48	<20	2,259	3,791	2,901	220	27	47	152	422	<20	<20	33	24
		40	56	<20	4,769	1,132	3,428	444	36	826	2,257	1,178	<20	131	192	153
		44	60	<20	6,601	9,276	9,141	401	399	1,789	2,038	1,420	<20	375	185	611
		52	68	<20	2,863	1,364	3,289	396	563	177	424	344	<20	76	72	38
		56	72	<20	1,992	2,380	2,886	298	1,834	86	766	245	<20	23	50	43
		65	81	<20	2,885	2,042	8,489	328	2,683	165	1,667	110	50	54	21	53
		74	90	<20	3,105	945	4,655	190	3,891	457	805	193	42	88	63	110
	V642	20	36	<20	2,741	3,627	1,075	502	<20	<20	<20	<20	<20	<20	<20	<20
		32	48	<20	2,846	5,999	1,153	509	<20	<20	<20	<20	<20	<20	<20	<20
		40	56	<20	1,330	7,052	6,325	718	<20	<20	<20	<20	<20	<20	<20	<20
		44	60	<20	4,188	7,315	1,089	725	<20	<20	<20	<20	<20	<20	<20	<20
	V644	20	36	<20	508	661	356	50	<20	<20	<20	<20	<20	<20	<20	<20
		32	48	<20	404	1,055	453	42	<20	<20	<20	<20	<20	<20	<20	<20
		40	56	<20	182	449	398	43	<20	<20	<20	<20	<20	<20	<20	<20
		44	60	<20	455	669	228	24	<20	<20	<20	<20	<20	<20	<20	<20
	V645	12	28	<20	695	710	217	42	<20	<20	<20	<20	<20	<20	<20	<20
		20	36	<20	10,621	12,311	9,970	223	<20	<20	360	<20	<20	<20	<20	<20
		24	40	<20	8,467	10,724	11,025	229	27	21	237	<20	<20	<20	<20	<20
		28	44	<20	16,461	16,532	13,378	489	374	85	377	21	<20	<20	<20	<20
		32	48	<20	12,561	17,153	19,759	1,369	425	288	551	42	56	<20	<20	<20
		40	56	<20	11,430	12,229	9,690	1,365	1,508	403	845	82	165	37	122	24
		44	60	<20	11,947	13,646	17,699	494	2,465	1,385	1,769	148	402	96	272	79
		52	68	<20	6,623	10,190	7,955	714	529	225	608	47	169	25	121	28
		56	72	<20	13,667	16,827	14,654	730	2,153	370	1,182	63	197	39	98	21
		65	81	<20	3,662	7,369	3,849	484	1,292	241	978	42	202	26	139	35
		74	90	<20	2,563	4,310	1,116	362	812	477	473	<20	28	24	50	<20
3	V650	16	N/A	<20	339	475	212	<20	<20	<20	<20	<20	<20	<20	<20	<20
		40	N/A	<20	3,315	655	928	108	<20	<20	227	<20	<20	<20	<20	<20
		44	N/A	<20	7,199	3,220	3,237	941	<20	<20	1,771	<20	142	<20	<20	<20
		48	N/A	<20	11,573	2,340	2,470	561	<20	26	5,241	<20	508	334	356	139
		52	N/A	<20	4,182	995	1,513	232	<20	<20	1,228	<20	369	47	89	35
		60	N/A	<20	956	439	398	170	<20	<20	261	<20	79	25	22	<20
		68	N/A	<20	1,099	258	502	162	<20	<20	171	<20	61	22	29	<20
		72	N/A	<20	993	461	298	629	<20	<20	90	<20	61	<20	<20	<20
		80	N/A	<20	547	229	265	30	<20	<20	20	<20	49	<20	<20	<20
		85	N/A	<20	313	951	683	73	<20	<20	68	<20	<20	<20	<20	<20
	V651	16	N/A	<20	4,062	1,677	1,529	<20	<20	<20	<20	<20	<20	<20	<20	<20
		40	N/A	<20	15,788	7,634	9,425	32	<20	<20	<20	<20	<20	<20	<20	<20
		44	N/A	<20	16,469	8,718	10,047	36	<20	<20	<20	<20	<20	<20	<20	<20
		48	N/A	<20	13,721	8,826	10,571	64	<20	<20	<20	<20	<20	<20	<20	<20
		52	N/A	<20	15,444	7,843	8,251	<20	<20	<20	<20	<20	<20	<20	<20	<20
		60	N/A	<20	10,801	7,072	8,453	36	<20	<20	<20	<20	<20	<20	<20	<20
		68	N/A	<20	8,425	7,278	7,740	39	<20	<20	<20	<20	<20	<20	<20	<20
		72	N/A	<20	16,258	4,815	8,244	44	<20	<20	<20	<20	<20	<20	<20	<20
		80	N/A	<20	5,112	1,857	3,720	<20	<20	<20	<20	<20	<20	<20	<20	<20
		85	N/A	<20	6,605	4,596	4,198	121	<20	<20	<20	<20	<20	<20	<20	<20
	CM03	16	N/A	<20	3,196	1,493	1,622	486	<20	<20	<20	<20	<20	<20	<20	<20
		20	N/A	<20	3,459	935	2,184	158	<20	<20	32	<20	<20	<20	25	<20
		24	N/A	<20	3,033	1,115	2,091	418	<20	<20	91	<20	<20	<20	44	<20
		28	N/A	<20	2,259	1,223	2,022	180	<20	23	65	<20	<20	<20	37	<20
		32	N/A	<20	2,409	998	1,746	387	<20	31	100	<20	<20	<20	40	<20
		36	N/A	<20	1,970	1,459	3,478	177	<20	31	43	<20	<20	<20	44	<20
		40	N/A	<20	1,800	2,131	2,386	380	<20	<20	37	<20	<20	<20	31	<20
		44	N/A	<20	2,225	1,648	2,821	209	<20	<20	49	20	<20	<20	<20	<20
		48	N/A	<20	1,368	1,311	2,664	62	<20	<20	23	<20	<20	<20	<20	<20
		52	N/A	<20	1,970	1,459	3,478	177	<20	31	43	<20	<20	<20	44	<20
		56	N/A	<20	521	558	878	42	<20	73	169	26	26	<20	41	27
		60	N/A	<20	217	266	570	26	<20	<20	24	<20	<20	<20	<20	<20
	CK36	16	N/A	<20	115	152	125	<20	<20	<20	<20	<20	<20	<20	<20	<20
		20	N/A	<20	433	276	148	<20	<20	<20	<20	<20	<20	<20	<20	<20
		24	N/A	<20	419	267	307	26	<20	<20	<20	<20	<20	<20	<20	<20
		28	N/A	<20	535	453	532	99	<20	<20	<20	<20	<20	<20	<20	<20
		32	N/A	<20	891	944	1,108	347	<20	<20	<20	<20	<20	<20	<20	<20
		36	N/A	<20	721	1,131	1,136	705	<20	<20	<20	<20	<20	<20	<20	<20
		40	N/A	<20	1,402	1,318	1,096	448	<20	<20	<20	<20	<20	<20	<20	<20
		44	N/A	<20	876	703	951	323	<20	<20	<20	<20	<20	<20	<20	<20
		48	N/A	<20	903	724	667	158	<20	<20	<20	<20	<20	<20	<20	<20
		52	N/A	<20	721	1,131	1,136	705	<20	<20	<20	<20	<20	<20	<20	<20
		56	N/A	<20	547	638	611	118	<20	<20	<20	<20	<20	<20	<20	<20
		60	N/A	<20	457	487	617	229	<20	<20	<20	<20	<20	<20	<20	<20
	NN58	16	N/A	<20	6,394	7,174	3,979	174	<20	<20	<20	<20	<20	<20	<20	<20
		20	N/A	<20	4,726	8,190	3,381	1,268	<20	<20	<20	<20	<20	<20	<20	<20
		24	N/A	<20	9,681	5,429	4,021	1,192	<20	<20	122	26	<20	<20	<20	<20
		28	N/A	<20	7,536	5,914	5,063	1,934	<20	<20	550	<20	<20	26	<20	<20
		32	N/A	<20	6,341	4,502	2,869	1,092	<20	<20	611	<20	<20	35	<20	<20
		36	N/A	<20	3,628	4,011	2,406	912	<20	<20	350	<20	<20	43	<20	<20
		40	N/A	<20	3,554	2,437	2,064	518	<20	<20	195	30	<20	41	<20	<20
		44	N/A	<20	2,481	2,045	2,244	627	<20	<20	701	34	<20	67	<20	<20
		48	N/A	<20	2,430	1,695	1,790	255	<20	<20	451	48	<20	68	<20	<20
		52	N/A	<20	3,628	4,011	2,406	912	<20	<20	350	54	<20	82	<20	<20
		56	N/A	<20	1,619	1,996	2,251	220	<20	<20	218	43	<20	31	<20	<20
		60	N/A	<20	903	867	861	113	<20	<20	140	<20	<20	<20	<20	<20
	NN39	16	N/A	<20	4,699	5,435	4,854	482	<20	<20	<20	<20	<20	<20	<20	<20
		20	N/A	<20	11,101	11,380	7,716	1,218	<20	<20	42	<20	<20	<20	<20	<20
		24	N/A	<20	13,891	6,969	5,640	1,310	<20	<20	1,436	<20	126	51	<20	<20
		28	N/A	<20	6,636	3,614	5,734	1,290	<20	38	1,169	<20	200	63	40	<20
		32	N/A	<20	8,071	4,699	16,340	1,721	32	354	2,404	20	980	481	426	395
		36	N/A	<20	13,161	5,444	35,714	1,323	193	1,000	4,630	139	3,448	1,680	1,082	1,380
		40	N/A	<20	9,025	4,785	55,648	1,413	307	171	1,723	106	493	216	411	317
	AM12	12	N/A	<20	52	113	55	<20	<20	<20	<20	<20	<20	<20	<20	<20
		16	N/A	<20	375	277	176	<20	<20	<20	<20	<20	<20	<20	<20	<20
		24	N/A	<20	7,364	572	1,633	3,472	65	558	923	53	125	73	<20	<20
		32	N/A	<20	4,352	2,077	2,859	721	347	536	696	353	202	369	184	35
		36	N/A	<20	13,495	2,149	5,373	1,069	5,464	2,332	2,189	1,465	741	552	227	124
		40	N/A	<20	4,836	1,196	3,515	607	3,034	1,308	1,179	581	345	321	172	52
		44	N/A	<20	9,398	2,817	6,435	846	5,602	2,471	2,255	1,400	995	623	446	250
		48	N/A	<20	1,856	180	1,482	356	2,413	1,362	730	517	391	336	114	81
		*51	N/A	<20	1,513	694	775	119	1,412	345	407	238	159	171	48	<20
	AJ09	12	N/A	<20	1,301	763	710	<20	<20	<20	<20	<20	<20	<20	<20	<20
		16	N/A	<20	3,018	3,233	183	<20	<20	<20	<20	<20	<20	<20	<20	<20
		24	N/A	<20	2,307	2,822	1,103	286	<20	21	24	<20	<20	<20	<20	<20
		32	N/A	<20	1,239	1,780	974	342	41	<20	<20	<20	<20	<20	<20	<20
		36	N/A	<20	644	996	362	148	23	<20	<20	<20	<20	<20	<20	<20
		40	N/A	<20	518	1,869	534	97	26	<20	149	<20	<20	<20	<20	<20
		44	N/A	<20	404	5,222	405	116	101	<20	96	<20	<20	<20	<20	<20
		48	N/A	<20	1,285	7,587	880	110	57	48	166	29	<20	<20	<20	32
		52	N/A	<20	3,181	10,555	1,491	143	145	159	627	31	<20	25	<20	75
		56	N/A	<20	2,608	3,609	1,306	123	167	216	682	123	64	41	62	120
		60	N/A	<20	3,435	9,042	1,559	60	44	24	181	32	<20	<20	32	27
		64	N/A	<20	15,326	9,452	980	102	85	126	259	68	<20	<20	83	81
		68	N/A	<20	3,519	4,407	1,256	158	73	196	962	32	25	<20	61	51
		76	N/A	<20	4,250	6,596	1,086	220	60	37	395	27	<20	<20	<20	<20
		80	N/A	<20	2,492	3,628	768	132	49	64	365	51	<20	<20	<20	<20
		84	N/A	<20	2,125	3,348	757	97	40	24	184	<20	<20	<20	<20	<20
	AH82	12	N/A	<20	85	<20	<20	<20	<20	<20	<20	<20	<20	<20	<20	<20
		16	N/A	<20	3,352	250	623	31	<20	<20	<20	<20	<20	<20	<20	<20
		24	N/A	<20	4,598	402	1,309	435	<20	20	40	<20	<20	<20	<20	<20
		32	N/A	<20	4,378	606	3,256	388	25	21	32	<20	<20	<20	<20	<20
		36	N/A	<20	4,097	546	2,917	459	<20	<20	<20	<20	<20	<20	<20	<20
		40	N/A	<20	2,473	382	2,007	328	34	52	28	<20	<20	<20	<20	<20
		44	N/A	<20	1,633	245	1,127	225	53	43	34	<20	<20	<20	<20	<20
		48	N/A	<20	3,274	1,361	2,124	218	37	33	<20	<20	<20	<20	<20	<20
		52	N/A	<20	2,335	262	1,204	265	26	<20	26	<20	<20	<20	<20	<20
		56	N/A	<20	1,203	159	622	158	<20	<20	<20	<20	<20	<20	<20	<20
		60	N/A	<20	1,076	217	596	151	<20	<20	<20	<20	<20	<20	<20	<20
		80	N/A	<20	824	401	500	220	<20	<20	<20	21	<20	<20	<20	<20
		84	N/A	<20	1,040	373	719	339	<20	<20	<20	<20	<20	<20	<20	<20
	AG99	12	N/A	<20	46	48	24	<20	<20	<20	<20	<20	<20	<20	<20	<20
		16	N/A	<20	300	364	223	<20	<20	<20	<20	<20	<20	<20	<20	<20
		24	N/A	<20	606	771	337	<20	<20	<20	<20	<20	<20	<20	<20	<20
		32	N/A	<20	333	402	273	<20	<20	<20	<20	<20	<20	<20	<20	<20
		56	N/A	<20	156	211	144	<20	<20	<20	<20	<20	<20	<20	<20	<20
		80	N/A	<20	84	174	74	<20	<20	<20	<20	51	<20	<20	<20	<20
		112	N/A	<20	75	76	61	<20	<20	<20	<20	<20	<20	<20	<20	<20
		116	N/A	<20	42	60	40	<20	<20	<20	<20	<20	<20	<20	<20	<20

Reciprocal ID_{50}

<20
20-100
100-1000
1000-10000
>10000

Fig. S2. SHIV.5MUT-infected rhesus macaques develop heterologous plasma neutralization breadth by week 20-32 post-infection. (A) Longitudinal plasma neutralization against the indicated viruses, expressed as reciprocal ID_{50}.

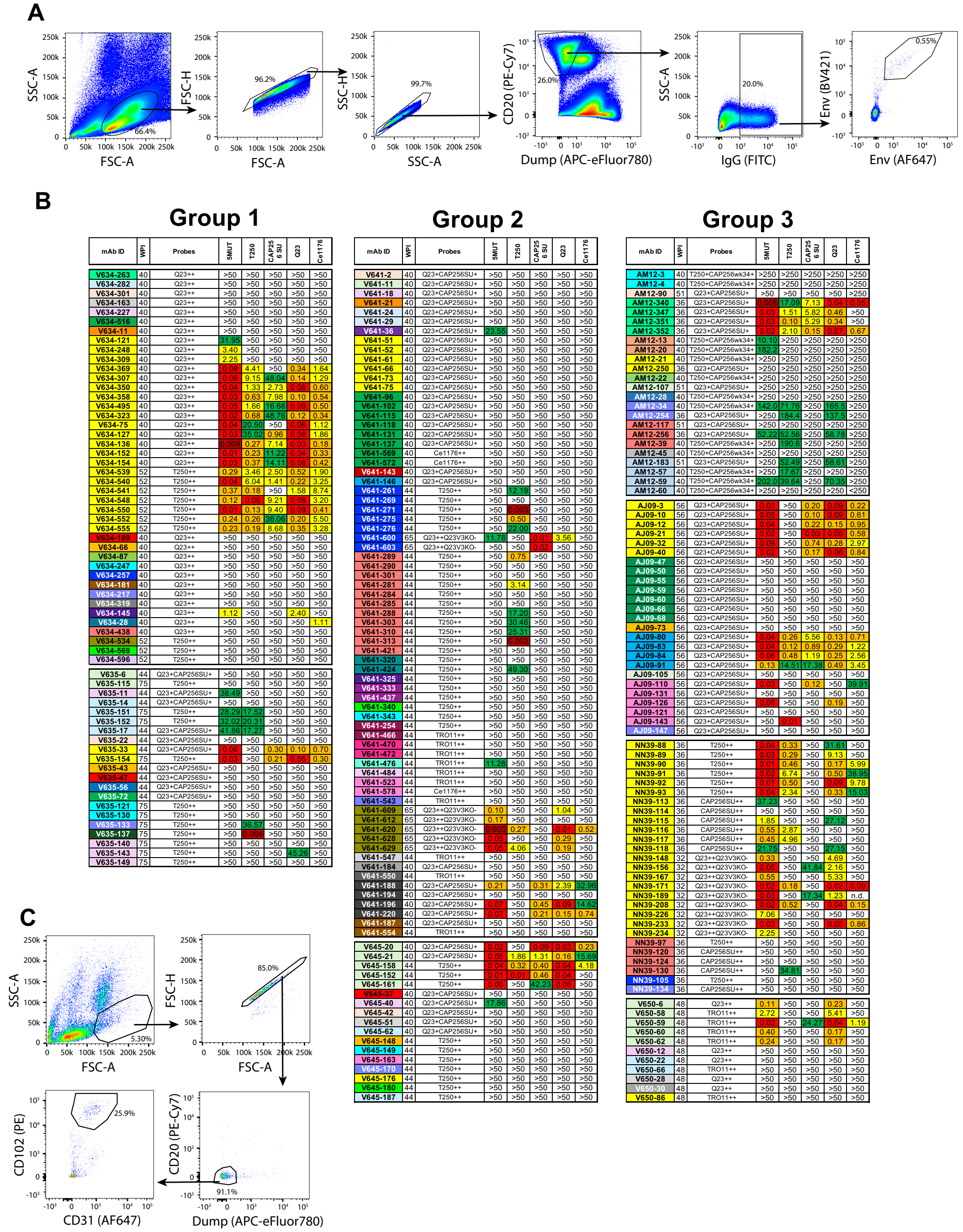

Fig. S3. Isolation of candidate bNAbs from SHIV.5MUT-infected macaques. (A) Representative gating strategy for the fluorescence-activated cell sorting used to isolate Env-binding B cells. Live $CD3^-CD8a^-CD14^-CD16^-CD20^+IgG^+Env^{++}$ B cells were sorted using Env trimer probes. **(B)** Monoclonal antibodies were synthesized and screened for neutralization activity against the autologous (SHIV.5MUT) and heterologous (T250, CAP256SU, Q23, and Ce1176) viruses. Titers are expressed as IC_{50} in μg/mL. The timepoint and Env probes used to isolate each B cell are listed. Within each animal, antibody names are color-coded by lineage. **(C)** Gating strategy for the isolation of bone marrow plasma cells. WPI, weeks post-infection.

Virus	Clone Name	Subtype	V1 length (C-to-C)	Glycan 332	324-336	AJ09-21	AJ09-83	AJ09-110	AM12 lineage AM12-347	AM12 lineage AM12-340	AM12 lineage AM12-352	AM12 lineage AM12-BM5	V635-33	V645-158	V650-59	V634 lineage V634-136	V634 lineage V634-540	V634 lineage V634-555	NN39-171	V641-220	V641-600	V641-620
5MUT	5MUT	A	17	332	GDIRQAHCNVSKA	0.030	0.040	0.030	0.033	0.008	0.015	0.009	0.061	0.035	0.018	0.009	0.044	0.232	0.016	0.065	11.780	0.003
A1.KE.1993.BG505_W6M_C2.DQ208458 T332N	BG505.T332N	A	17	332	GDIRQAHCNVSKA	4.580	0.270	2.030	2.010	0.039	0.100	0.077	0.092	1.030	>50	0.191	1.064	1.400	0.102	0.384	>50	0.504
B.US.1995.6535_3.AY835438	6535.3	B	27	332	GDIRQAHCNISRA	0.019	0.084	0.013	>50	0.105	0.084	0.027	0.020	0.011	0.110	0.182	0.031	0.159	0.014	>50	>50	0.300
B.US.1995.6240_08_TA5_4622.EU289190	6240_08_TA5_4622	B	31	332	GDIRQAHCNLSSK	39.505	2.134	0.863	>50	0.401	3.773	0.512	0.230	0.963	33.220	0.553	2.462	>50	0.012	>50	>50	>50
B.IT.1995.TRO_11.AY835445	TRO.11	B	30	332	GDIRQAHCNISRT	4.514	0.566	>50	1.713	0.529	0.429	0.135	0.222	4.998	>50	0.154	0.908	0.222	0.020	>50	>50	>50
B.US.1998.1056_10_TA11_1826.EU289186	1056_10_TA11_1826	B	25	332	GDIRQAHCNISGE	>50	2.623	>50	0.128	0.017	0.042	0.009	0.192	2.742	>50	1.223	0.881	>50	>50	>50	>50	0.192
B.IT.1996.PVO_4.AY835444	PVO.4	B	29	332	GDIRQAHCNLSRA	>50	9.601	>50	>50	3.956	3.817	0.200	0.218	2.917	>50	6.444	8.122	1.751	3.972	>50	>50	>50
B.TT.1994.QH0692_42.AY835439	QH0692.42	B	29	332	GDIRQAHCNLSSV	>50	4.478	>50	>50	1.483	2.006	0.266	0.245	>50	>50	0.756	13.664	35.161	>50	>50	>50	>50
B.US.1998.AC10_29.AY835446	AC10.0.29	B	25	332	GDIRQAHCNISRQ	>50	40.245	>50	>50	1.476	49.062	0.145	0.021	>50	>50	>50	13.356	>50	0.357	>50	>50	42.939
B.US.2000.RHPA4259_7.AY835447	RHPA4259.7	B	21	332	GDIRQAHCNISRE	0.061	0.220	>50	>50	>50	7.693	0.023	0.057	0.463	>50	0.128	0.467	1.494	>50	>50	>50	>50
B.US.1990.WEAU_D15_410.EU289202	WEAU_d15_410_787	B	30	332	GDIRRAHCNLSRT	>50	13.319	0.545	>50	>50	0.616	0.090	0.178	1.320	>50	8.348	3.269	15.185	0.076	>50	>50	>50
B.US.2004.CAAN5342.AY835452	CAAN5342.A2	B	22	332	GDIRKAHCNISGE	>50	49.129	1.580	>50	>50	0.146	0.010	0.615	0.176	>50	>50	0.838	>50	3.771	>50	>50	7.248
B.X2278_C2_B6.FJ817366	X2278	B	27	332	GDIRKAYCNISAT	>50	>50	2.150	>50	>50	2.947	3.505	>50	0.520	12.900	>50	>50	20.290	0.028	>50	>50	>50
B.US.1996.6244_13_B5_4567.EU289191	6244_13_B5_4576	B	37	332	GDIRQAHCNISRA	>50	4.096	>50	>50	>50	0.422	0.097	>50	6.721	>50	0.534	5.487	20.968	21.780	>50	>50	>50
B.US.2001.TRJO4551_58.AY835450	TRJO4551.58	B	32	332	GDIRKAYCNISEA	>50	>50	>50	>50	>50	0.655	0.076	>50	1.359	>50	>50	>50	>50	>50	>50	>50	>50
B.TT.1993.SC05_8C11_2344.EU289200	SC05_8C11_2344	B	20	332	GDIRQAHCNLSNT	2.910	1.078	1.455	37.288	>50	>50	0.454	0.053	0.125	17.166	1.890	2.958	47.689	0.017	10.822	>50	>50
B.US.1997.1012_11_TC21_3257.EU289184	1012_11_TC21_3257	B	28	332	GNIRQAHCNISRS	>50	2.852	>50	>50	>50	>50	>50	>50	>50	>50	1.019	7.217	>50	>50	>50	>50	>50
B.TT.1995.SC422_8.AY835441	SC422661.8	B	22	332	GDIRQVHCNLSSA	13.155	>50	>50	>50	>50	>50	>50	0.100	>50	>50	47.796	>50	>50	>50	>50	>50	>50
B.US.2000.WITO4160_33.AY835451	WITO4160.33	B	23	332	GDIRKAHCNISTE	>50	>50	>50	>50	>50	>50	2.341	>50	>50	>50	>50	>50	>50	>50	>50	>50	>50
B.US.1997.1006_11_C3_1601.EU289183	1006_11_C3_1601	B	21	332	GDIKRAYCNISRT	>50	>50	>50	>50	>50	>50	>50	>50	0.044	>50	1.626	>50	0.486	>50	>50	0.012	>50
B.US.1997.1054_07_TC4_1499.EU289185	1054_07_TC4_1499	B	29	332	GDIRKAYCNISRA	>50	>50	>50	>50	>50	>50	0.187	>50	>50	>50	>50	>50	>50	>50	>50	>50	>50
B.US.1997.62357_14_D3_4589.EU289189	62357_14_D3_4589	B	18	332	GDIRQAYCNISRA	>50	>50	>50	>50	>50	>50	>50	>50	>50	>50	>50	>50	>50	>50	>50	>50	>50
B.US.2001.REJO4541_67.AY835449	REJO4541.67	B	27	334	GDIRKAYCTINES	>50	>50	>50	>50	>50	>50	>50	>50	>50	>50	>50	>50	>50	>50	>50	>50	>50
B.US.2000.THRO4156.AY835448	THRO4156.18	B	33	334	GDIRKAYCTVNGT	>50	>50	>50	>50	>50	>50	>50	>50	>50	>50	>50	>50	>50	>50	>50	>50	>50
C.ZA.1999.DU156_12.DQ411852	Du156.12	C	23	332	GDIRQAHCNISRN	0.108	0.352	6.054	>50	0.099	3.078	0.280	0.187	0.913	1.477	0.246	0.649	8.116	0.017	3.328	>50	4.623
C.ZA.2005.CAP256_SU.KF241776	CAP256.SU	C	20	332	GDIRQAHCNISEI	0.020	1.270	0.050	5.819	7.529	0.146	0.046	0.300	0.399	24.270	7.137	1.406	8.680	>50	0.205	0.011	>50
C.ZA.1998.DU422_1.DQ411854	Du422.1	C	25	332	GDIREAHCNISRE	2.107	26.124	>50	45.467	41.187	0.501	0.085	0.326	6.319	1.842	39.098	>50	>50	0.022	>50	>50	17.447
C.MW.2004.CE1176_A3.FJ444437	Ce1176_A3	C	21	332	GDIRQAHCNVSKQ	1.279	1.077	>50	>50	0.058	0.762	0.088	0.244	5.050	3.163	0.452	3.186	3.944	0.042	1.926	>50	21.202
C.IN.1999.25710_2_43.EF117271	25710	C	14	332	GDIRQAHCNISKD	33.780	0.820	>50	>50	0.095	0.111	0.052	14.390	>50	>50	0.094	0.387	0.488	6.398	0.140	>50	7.876
C.ZA.1998.DU172_17.DQ411853	Du172.17	C	28	332	GDIRQAHCNISRK	>50	15.663	>50	>50	0.023	2.788	0.501	17.283	>50	>50	2.825	45.341	>50	>50	>50	>50	>50
C.MW.2004.CE1172_H1.FJ444434	Ce1172_H1	C	23	332	GDIRQAHCNISRE	>50	0.742	>50	>50	0.038	2.268	0.123	0.106	>50	>50	0.239	1.718	2.822	0.158	>50	>50	>50
C.ZM.2003.ZM247_V1.FJ496204	ZM247v1(Rev-)	C	20	332	GDIREAHCNITKH	>50	8.305	>50	>50	44.267	1.618	0.138	0.388	>50	>50	0.743	>50	>50	>50	>50	>50	>50
C.MW.2004.1394_C9G1.FJ444527	1394C9G1(Rev-)	C	32	332	GDIRQAHCNISKA	>50	0.268	>50	0.673	0.107	0.150	0.019	0.156	>50	>50	4.210	>50	>50	1.757	1.266	>50	0.477
C.ZA.2007.704809221_1B3.FJ444103	Ce704809221_1B3	C	34	332	GDIRQAHCNISEE	>50	9.764	>50	>50	0.122	0.844	0.086	2.121	>50	>50	28.757	>50	>50	7.738	>50	>50	0.717
C.ZM.2003.ZM214_15.DQ388516	ZM214M.PL15	C	26	332	GDIRQAHCNISKD	>50	>50	>50	48.187	0.677	1.245	0.118	2.406	>50	>50	>50	>50	>50	>50	0.312	>50	>50
C.CN.2007.CNE17.HM215403	CNE17	C	44	332	GDIRQAHCNISKG	>50	33.732	>50	>50	0.633	5.629	0.596	>50	>50	>50	11.929	>50	>50	>50	30.875	>50	>50
C.CH1012	CH1012	C	27	332	GDIRRAYCNISAE	>50	>50	>50	>50	23.490	0.610	0.314	>50	>50	>50	>50	>50	>50	>50	>50	1.828	>50
C.MW.2003.CE0682_E4.FJ444325	Ce0682_E4	C	27	332	GDIRQAHCNISDE	>50	>50	>50	0.048	>50	0.020	0.006	>50	>50	>50	>50	>50	>50	>50	>50	>50	>50
C.CE703010217_B6.FJ443575	Ce0217	C	15	332	GDIRRAYCNISEK	>50	>50	>50	>50	>50	8.332	0.264	>50	27.550	12.390	>50	>50	17.020	0.026	>50	>50	>50
C.IN.2000.001428_2_42.EF117266	HIV-001428-2.42	C	25	332	GNIREAHCNISEK	>50	0.588	>50	>50	12.382	>50	0.279	>50	>50	>50	11.423	>50	>50	>50	>50	>50	>50
C.IN.1999.16845_2_22.EF117269	HIV-16845-2.22	C	31	332	GDIRQAHCNISEA	>50	26.393	>50	>50	22.610	>50	29.934	>50	>50	>50	27.530	>50	>50	>50	>50	>50	>50
C.ZM.2003.ZM246F.FJ496194	246F C1G	C	12	332	GDIREAHCNISST	>50	>50	>50	>50	0.029	>50	>50	>50	>50	>50	>50	25.824	>50	>50	>50	>50	>50
C.CH848_3_d0017_10_02.KX216883	CH848	C	32	332	GDIRQAHCNISER	>50	>50	>50	>50	16.220	>50	36.590	>50	>50	>50	>50	>50	>50	>50	>50	>50	>50
C.MW.2007.703010200_1E5.FJ443999	7030102001E5(Rev-)	C	21	332	GDIRQAHCNISKK	3.196	0.058	0.301	>50	>50	>50	0.097	0.007	0.048	>50	0.039	0.029	0.126	0.413	32.201	0.014	0.357
C.TZ.2003.3301_V1_C24.HM215294	3301.v1.c24	C	19	332	GDIRQAHCNITRN	>50	0.975	>50	>50	>50	3.946	0.347	0.209	>50	>50	18.696	0.822	0.932	0.543	>50	>50	>50
C.ZM.2002.ZM233_6.DQ388517	ZM233M.PB6	C	15	332	GNIREAHCNISAS	>50	0.168	>50	>50	>50	>50	>50	>50	>50	>50	0.654	>50	1.142	>50	42.564	>50	>50
C.ZM.1998.ZM135_10A.AY424079	ZM135M.PL10a	C	21	332	GDIRQAHCNISGE	>50	47.228	>50	>50	>50	>50	41.736	25.306	>50	>50	>50	>50	>50	0.810	>50	>50	>50
C.ZA.2005.CAP210_E8.DQ435683	CAP210.2.00.E8	C	17	332	GNIREAHCNISEK	>50	46.928	>50	>50	>50	>50	>50	>50	>50	>50	>50	>50	>50	>50	>50	>50	>50
C.CN.2006.CNE58.HM215421	CNE58	C	37	332	GDIRQAHCNITKS	>50	>50	>50	>50	>50	>50	>50	>50	>50	>50	>50	>50	>50	>50	>50	>50	>50
C.ZM.2003.ZM249_1.DQ388514	ZM249M.PL1	C	21	332	GKIREAHCNISKE	>50	>50	>50	>50	>50	>50	>50	>50	>50	>50	>50	>50	>50	>50	>50	>50	>50
C.MW.2005.CE2060_G9.FJ444607	Ce2060_G9	C	32	332	GNIRQAYCNITKG	>50	>50	>50	>50	>50	>50	>50	>50	>50	>50	>50	>50	>50	>50	>50	>50	>50
C.IN.2000.0013095_2_11.EF117267	HIV-0013095-2.11	C	22	332	GDIRQARCNISEE	>50	>50	>50	>50	>50	>50	>50	>50	>50	>50	>50	>50	>50	>50	>50	>50	>50
C.ZM.2003.249M_B10.EU166862	249M B10	C	21	332	GKIREAHCNISKE	>50	>50	>50	>50	>50	>50	>50	>50	>50	>50	>50	>50	>50	>50	>50	>50	>50
C.MW.2002.BF1266_431A.HM215360	BF1266.431a	C	24	334	GDTRQAYCTINGT	>50	>50	>50	>50	>50	0.631	0.389	>50	>50	>50	>50	>50	>50	>50	>50	>50	>50
C.MW.2008.CH505_TF.KC247556	CH505	C	16	334	GDIREAYCNINES	>50	>50	>50	>50	>50	2.106	1.428	>50	>50	>50	>50	>50	>50	>50	>50	>50	>50
C.ZM.2000.ZM109_4.AY424138	ZM109F.PB4	C	15	334	GDIRKAYCKINGS	>50	>50	>50	>50	>50	>50	>50	>50	>50	>50	>50	>50	>50	>50	>50	>50	>50
C.ZA.2005.CAP45_G3.DQ435682	CAP45.2.00.G3	C	15	334	GDIRQAHCNINNS	>50	>50	>50	>50	>50	>50	>50	>50	>50	>50	>50	>50	>50	>50	>50	>50	>50
C.MW.2004.CE1086_B2.FJ444395	Ce1086_B2	C	16	334	GNIRQAHCNINES	>50	>50	>50	>50	>50	>50	>50	>50	>50	>50	>50	>50	>50	>50	>50	>50	>50
C.MW.2005.CE2010_F5.FJ444561	Ce2010_F5	C	31	334	GNIRQAHCTINAT	>50	>50	>50	>50	>50	>50	>50	>50	>50	>50	>50	>50	>50	>50	>50	>50	>50
C.MW.2007.703010054_2A2.FJ443808	Ce703010054_2A2	C	15	334	GDIRQAHCDINGT	>50	>50	>50	>50	>50	>50	>50	>50	>50	>50	>50	>50	>50	>50	>50	>50	>50
C.ZM.2000.ZM53_12.AY423984	ZM53M.PB12	C	13	none	GDIRQAYCIINKA	>50	>50	>50	>50	>50	0.818	0.045	>50	>50	>50	>50	>50	>50	>50	>50	>50	>50
C.ZM.2002.ZM197_7.DQ388515	ZM197M.PB7	C	37	none	GDIRQAHCDLSKS	>50	>50	>50	>50	>50	>50	>50	>50	>50	>50	>50	>50	>50	>50	>50	>50	>50
C.IN.1999.16055_2_3.EF117268	HIV-16055-2.3	C	22	none	GNIRQAYCNIKKD	>50	>50	>50	>50	>50	>50	>50	>50	>50	>50	>50	>50	>50	>50	>50	>50	>50
C.MW.2003.CE0393_C3.FJ444215	Ce0393_C3	C	32	none	GDIRQASCNIDEK	>50	>50	>50	>50	>50	>50	>50	>50	>50	>50	>50	>50	>50	>50	>50	>50	>50
07_BC.CN.2004.CH119_10.EF117261	CH119	BC	19	332	GDIRQAHCNISKW	0.080	0.240	>50	>50	3.325	2.971	0.422	0.037	45.080	7.068	0.062	0.437	0.947	0.029	12.000	>50	17.320
07_BC.CN.2007.BJOX002000_03_2.HM215364	BJOX002000	BC	20	332	GDIRQAHCNISGK	5.510	1.210	0.450	>50	14.580	0.484	0.065	0.012	0.940	12.760	0.040	2.436	7.846	0.018	>50	>50	38.880
07_BC.CN.2007.CNE21.HM215407	CNE21	BC	24	332	GDIRQAHCNISET	>50	0.305	>50	>50	0.024	0.666	0.028	13.382	>50	>50	0.037	0.411	0.402	1.036	30.481	>50	9.944
07_BC.CN.2007.CNE20.HM215406	CNE20	BC	15	332	GNIRQAHCNISEN	31.652	0.027	>50	>50	>50	45.624	0.011	>50	>50	>50	0.007	0.111	0.032	>50	0.027	>50	1.958
08_BC.CN.2007.CNE53.HM215417	CNE53	BC	18	332	GDIRQAHCNISES	0.291	0.558	>50	>50	0.585	>50	0.819	0.319	15.932	19.139	0.153	0.549	0.326	0.037	2.035	>50	>50
08_BC.CN.2007.CNE30.HM215411	CNE30	BC	23	332	GDIRQAHCNISRN	>50	21.972	>50	>50	>50	>50	>50	4.944	>50	>50	4.875	38.840	>50	1.582	>50	>50	>50
08_BC.CN.2007.CNE52.HM215416	CNE52	BC	23	332	GDIRQAHCNISKE	>50	>50	>50	>50	>50	>50	>50	>50	>50	>50	>50	>50	>50	>50	>50	>50	>50
07_BC.CN.2007.CNE19.HM215405	CNE19	BC	20	332	GDIRQAYCNISKD	>50	>50	>50	>50	>50	>50	1.872	>50	>50	>50	>50	>50	1.432	>50	>50	0.022	>50
A1.KE.1994.Q23_17.AF004885	Q23.17	A	17	332	GDIRQAHCNVTRS	0.050	0.234	0.006	0.193	0.018	0.077	0.010	0.019	0.011	0.016	0.061	0.130	0.158	0.003	0.057	0.222	0.075
A1.UG.2007.9004SS_A3_4.HM215350	9004SS_A3_4	A	31	332	GNIRQAHCNVSKA	>50	0.204	>50	38.799	8.051	1.944	0.018	25.787	>50	>50	>50	10.368	25.113	>50	>50	>50	>50
A1.TZ.2005.0260_V5_C36.HM215256	0260.v5.c36	A	32	332	GDIRQAHCNVSKA	>50	20.377	9.558	>50	>50	3.821	0.778	>50	8.242	>50	3.181	15.435	>50	0.107	>50	>50	>50
A1.TZ.2004.3365_V2_C20.HM215298	3365.v2.c2	A	20	332	EDIREAHCNVSQT	>50	2.149	>50	>50	>50	1.168	0.028	0.535	>50	>50	>50	12.049	>50	0.061	3.622	>50	>50
A1D.KE..MS208_A1.DQ187010	MS208.A1	A	21	332	GDIRQAHCNVSGS	>50	>50	>50	>50	>50	0.148	0.031	>50	>50	>50	>50	>50	>50	>50	>50	>50	>50
A1.UG.2007.191084_B7_19.HM215266	191084 B7-19	A	25	332	GDIRQAHCNVSES	>50	12.385	1.407	>50	>50	>50	3.840	0.029	0.096	2.853	>50	23.794	>50	0.015	1.803	>50	>50
A1.TZ.2003.3415_V1_C1.HM215299	3415.v1.c1	A	22	332	GGIRQAHCNVSRK	>50	1.576	>50	>50	>50	>50	>50	>50	>50	>50	0.623	20.837	21.036	>50	>50	>50	>50
A1.KE.1994.Q842_D12.AF407160	Q842.d12	A	17	334	GDIRQAHCNVNRT	>50	>50	>50	>50	1.404	3.652	0.919	>50	>50	>50	>50	>50	>50	>50	>50	>50	>50
A1.KE.1994.Q259_17.AF407152	Q259.d2.17	A	19	334	GNIRQAYCTVNRT	>50	>50	>50	>50	>50	1.932	0.021	>50	>50	>50	>50	>50	>50	>50	>50	>50	>50
A1.UG.2007.191955_A11.HM215272	191955_A11	A	21	334	GDIRQAYCKVNKS	>50	>50	>50	>50	>50	>50	>50	>50	>50	>50	>50	>50	>50	>50	>50	>50	>50
A1.KE.1995.Q461_E2.AF407156	Q461.e2	A	25	334	GDIRNAHCVVNRT	>50	>50	>50	>50	>50	>50	8.823	>50	>50	>50	>50	>50	>50	>50	>50	>50	>50
A1.KE.1996.Q769_D22.AF407158	Q769.d22	A	22	none	GDIRKAHCNVNRQ	>50	>50	>50	>50	>50	2.710	>50	>50	>50	>50	>50	>50	>50	>50	>50	>50	>50
02_AG.CI.1999.928_28.EU513199	928-28	AG	23	332	GDIRQAHCNVSKI	>50	>50	>50	>50	>50	>50	>50	>50	>50	>50	>50	>50	>50	>50	>50	>50	>50
02_AG.CM.2004.263_8.EU513182	263-8	AG	27	332	GDIRQAYCNVSKG	>50	>50	>50	>50	>50	>50	>50	>50	0.642	>50	>50	>50	0.652	>50	>50	>50	>50
02_AG.CM.2004.T250_4.EU513189	T250-4	AG	20	332	GNIRKAYCNVSKR	>50	0.301	>50	13.903	3.330	22.602	0.012	>50	0.525	>50	0.813	22.163	0.173	>50	>50	0.004	>50
02_AG.CM.2004.T251_18.EU513196	T251-18	AG	22	332	GDIRQAHCNVSRG	>50	4.881	>50	>50	>50	>50	0.317	>50	>50	>50	>50	>50	>50	>50	>50	>50	>50
02_AG.CM.2005.T278_50.EU513198	T278-50	AG	24	332	GDIRQAYCNVSGT	>50	>50	>50	>50	>50	>50	1.267	>50	1.188	>50	>50	>50	>50	>50	>50	>50	>50
02_AG.CM.2004.T255_34.EU513184	T255-34	AG	26	332	GDIRQAHCNVSK	>50	0.162	>50	>50	>50	0.061	0.008	>50	>50	>50	>50	31.629	0.280	>50	>50	>50	>50
02_AG.CM.2004.211_9.EU513187	211-9	AG	32	332	GNIRQAHCNVSRA	>50	>50	>50	>50	>50	>50	>50	>50	>50	>50	>50	>50	>50	>50	>50	>50	>50
02_AG.CM.2004.235_47.EU513195	235-47	AG	20	332	GDIRQAHCNVSST	>50	>50	>50	>50	>50	>50	>50	>50	>50	6.484	>50	>50	>50	5.762	>50	>50	>50
02A1.CM.2004.T257_31.EU513185	T257-31	AG	31	334	GDIRQAHCKVNKT	>50	>50	>50	>50	40.754	10.319	6.449	>50	>50	>50	>50	>50	>50	>50	>50	>50	>50
01_AE.CN.2007.BJOX028000_10_3.HM215389	BJOX028000.10.3	AE	29	332	GDIRQAYCNISRT	>50	>50	>50	>50	>50	>50	0.960	>50	>50	>50	>50	>50	>50	>50	>50	>50	>50
01_AE.TH.1999.C1080_C3.JN944660	C1080.c03	AE	26	334	GDIRKAYCEINGT	>50	>50	>50	>50	>50	5.819	0.798	>50	>50	>50	>50	>50	>50	>50	>50	>50	>50
01_AE.TH.1999.C2101_C1.JN944661	C2101.c01	AE	26	334	GDIRAVYCEINET	>50	>50	>50	>50	>50	1.271	0.250	>50	>50	>50	>50	>50	>50	>50	>50	>50	>50
01_AE.CN.2006.CNE5.HM215415	CNE5	AE	28	334	GDIRKAYCEINGT	>50	>50	>50	>50	>50	8.675	4.108	>50	>50	>50	>50	>50	>50	>50	>50	>50	>50
01_AE.CN.2006.CNE8.HM215427	CNE8	AE	24	334	GDIRKAYCEINRT	>50	>50	>50	>50	>50	0.975	0.231	>50	>50	>50	>50	>50	>50	>50	>50	>50	>50
01_AE.CN.2007.BJOX009000_02_4.HM215372	BJOX009000.02.4	AE	25	334	GDIRKAYCEINGT	>50	>50	>50	>50	>50	0.640	0.389	>50	>50	>50	>50	>50	>50	>50	>50	>50	>50
01_AE.CN.2007.BJOX010000_06_2.HM215373	BJOX010000.06.2	AE	32	334	GDIRKAYCKINAT	>50	>50	>50	>50	>50	0.304	0.216	>50	>50	>50	>50	>50	>50	>50	>50	>50	>50
01_AE.CN.2007.BJOX015000_11_5.HM215377	BJOX015000.11.5	AE	29	334	GDIRKAYCEINGT	>50	>50	>50	>50	>50	0.318	0.135	>50	>50	>50	>50	>50	>50	>50	>50	>50	>50
01_AE.CN.2007.BJOX025000_01_1.HM215386	BJOX025000.01.1	AE	26	334	GDIRQAYCEINGT	>50	>50	>50	>50	>50	29.184	3.422	>50	>50	>50	>50	>50	>50	>50	>50	>50	>50
01_AE.TH.1999.C3347_C11.JX512902	C3347.c11	AE	30	334	GNIRKAYCEINGT	>50	>50	>50	>50	>50	>50	0.166	>50	>50	>50	>50	>50	>50	>50	>50	>50	>50
01_AE.TH.1999.R3265_C6.JX512903	R3265.c06	AE	27	334	GDIRQAYCEINQT	>50	>50	>50	>50	>50	>50	7.964	>50	>50	>50	>50	>50	>50	>50	>50	>50	>50
01_AE.TH.2001.R2184_C4.JN944665	R2184.c04	AE	28	334	GNIRKAYCVINGT	>50	>50	>50	>50	>50	>50	>50	>50	>50	>50	>50	>50	>50	>50	>50	>50	>50
01_AE.TH.2005.620345_C1.JX512900	620345.c01	AE	27	334	TSIRKAYCEINGT	>50	>50	>50	>50	>50	>50	>50	>50	>50	>50	>50	>50	>50	>50	>50	>50	>50
01_AE.TH.1998.R1166_C1.JQ352783	R1166.c01	AE	39	334	GDIRRAYCEINRT	>50	>50	>50	>50	>50	>50	>50	>50	>50	>50	>50	>50	>50	>50	>50	>50	>50
01_AE.CN.2007.CNE55.HM215418	CNE55	AE	23	none	GDIRKAYCEIDGT	>50	>50	>50	>50	>50	7.196	2.139	>50	>50	>50	>50	>50	>50	>50	>50	>50	>50
01_AE.TH.2000.C4118_9.JQ352782	C4118.c09	AE	27	none	GDIRNAYCEIDGT	>50	>50	>50	>50	>50	>50	>50	>50	>50	>50	>50	>50	>50	>50	>50	>50	>50
G.ES.2008.P1981_C5_3.FJ817369	P1981_C5_3	G	19	332	GDIRQAHCNISKS	0.084	0.147	0.014	>50	0.069	0.836	0.034	0.033	0.080	>50	2.832	0.118	0.055	0.039	0.021	>50	0.072
G.ES.2003.X1254_C3.EU885762	X1254_c3	G	24	332	GDIRQAHCNISGT	>50	2.169	>50	>50	0.084	1.319	0.122	4.303	>50	>50	>50	1.875	1.853	0.286	46.377	>50	0.336
G.ES.2002.X1193_C1.EU885761	X1193_c1	G	28	332	GDIRQAHCNISKT	>50	2.436	>50	>50	0.109	0.506	0.080	27.541	>50	>50	0.649	10.408	>50	>50	0.236	>50	1.868
G.ES.2005.X2131_C1_B5.FJ817368	X2131_C1_B5	G	28	332	GDIRQAHCNISKT	>50	1.099	>50	>50	0.251	0.847	0.207	1.907	>50	>50	0.272	0.655	0.765	0.209	0.090	>50	28.042
G.ES.2002.P0402_C2_11.EU885759	P0402_c2_11	G	22	332	GNIRQAHCNISGA	>50	0.365	>50	>50	4.191	>50	0.059	47.673	>50	>50	>50	39.917	>50	>50	>50	>50	>50
G.ES.2006.X2088_C9.EU885764	X2088_c9	G	13	332	GDIRQAHCNVTRG	>50	9.522	4.193	>50	>50	>50	>50	0.065	>50	0.018	>50	>50	>50	0.002	>50	>50	>50
G.ES.2004.X1632_S2_B10.FJ817370	X1632_S2_B10	G	25	334	GDTRQAHCNINGS	>50	>50	>50	>50	>50	>50	>50	>50	>50	>50	>50	>50	>50	>50	>50	>50	>50
D.UG.1999.A07412M1_VRC12.HM215357	A07412M1.vrc12	D	23	332	GNIRQAHCNVSKV	>50	21.609	>50	>50	>50	>50	16.774	>50	>50	>50	10.641	>50	>50	>50	>50	>50	>50
D.TZ.2004.6405_V4_C34.HM215327	6405.v4.c34	D	28	332	GDIRQAHCNVSRV	>50	1.888	>50	>50	>50	>50	0.202	3.092	>50	>50	1.760	3.041	8.007	>50	>50	>50	>50
D.UG.2001.231965_C1.JQ361079	231965.c01	D	34	332	GNIRLAYCNISRK	>50	>50	>50	>50	>50	>50	>50	>50	>50	>50	14.227	>50	>50	>50	>50	1.597	>50
D.UG.2001.231966_C2.JX512899	231966.c02	D	30	332	GDIRQAYCNISET	>50	>50	>50	>50	>50	>50	2.463	>50	>50	>50	>50	>50	>50	>50	>50	>50	>50
D.TZ.2005.3016_V5_C45.HM215283	3016.v5.c45	D	21	none	GDIRQAYCTIDGT	>50	>50	>50	>50	>50	>50	>50	>50	>50	>50	>50	>50	>50	>50	>50	>50	>50
CD.TZ.2006.6811_V7_C18.HM215340	6811.v7.c18	CD	11	332	GDIRQAHCNISES	0.153	0.275	0.042	>50	0.147	0.130	0.053	0.012	0.486	0.008	0.444	0.263	11.736	0.002	0.558	>50	13.046
CD.TZ.2004.6480_V4_C25.HM215329	6480.v4.c25	CD	19	332	GDIRKAHCNISKD	>50	10.315	0.182	>50	4.024	0.068	0.020	0.050	0.129	0.708	5.593	0.786	1.453	0.008	>50	>50	>50
CD.TZ.2004.3817_V2_C59.HM215310	3817.v2.c59	CD	21	332	GDIRQAHCNISRG	>50	>50	>50	>50	43.877	8.067	0.151	9.703	>50	>50	>50	>50	>50	23.449	>50	>50	>50
41_CD.TZ.2003.6952_V1_C20.HM215343	6952.v1.c20	CD	28	332	GDIRQAHCNISGT	>50	0.648	>50	>50	>50	>50	>50	11.070	>50	>50	0.080	5.668	0.792	>50	2.668	>50	>50
CD.TZ..89_F1_2_25.HM215349	89-F1_2_25	CD	14	none	GDIRKAYCTISKG	>50	>50	>50	>50	>50	>50	>50	>50	>50	>50	>50	>50	>50	>50	>50	>50	>50
A1CD.TZ.2004.3103_V3_C10.HM215288	3103.v3.c10	A1CD	23	332	GDIRQAHCNISEA	44.839	0.392	>50	0.626	0.539	1.106	0.184	0.158	4.763	29.943	0.080	0.311	0.313	0.054	>50	>50	14.604
A1D.TZ.2004.0815_V3_C3.HM215260	0815.v3.c3	A1D	26	332	GDIRQAHCNVSRA	>50	>50	2.838	>50	>50	3.735	0.172	0.806	>50	1.163	>50	7.882	>50	0.089	>50	>50	>50
A1C.TZ.2004.6041_V3_C23.HM215321	6041.v3.c23	A1C	26	334	GNIRQAHCDINRT	>50	>50	>50	>50	>50	>50	>50	>50	>50	>50	>50	>50	>50	>50	>50	>50	>50
A1C.TZ.2004.6540_V4_C1.HM215330	6540.v4.c1	A1C	23	334	GNIRQAYCTVNRT	>50	>50	>50	>50	>50	>50	1.174	>50	>50	>50	>50	>50	>50	>50	>50	>50	>50
A1C.TZ.2005.6545_V4_C1.HM215332	6545.v4.c1	A1C	23	334	GNIRQAYCTVNRT	>50	>50	>50	>50	>50	>50	14.385	>50	>50	>50	>50	>50	>50	>50	>50	>50	>50
A1C.TZ.246_F3_C10_2.HM215279	246F3	A1C	20	334	GNIRQAHCTVNKT	>50	>50	>50	>50	>50	>50	7.004	>50	>50	>50	>50	>50	>50	>50	>50	>50	>50
MuLV						>50	>50	>50	>50	>50	>50	>50	>50	>50	>50	>50	>50	>50	>50	>50	>50	>50

	AJ09-21	AJ09-83	AJ09-110	AM12-347	AM12-340	AM12-352	AM12-BM5	V635-33	V645-158	V650-59	V634-136	V634-540	V634-555	NN39-171	V641-220	V641-600	V641-620
Whole Panel	15.4%	44.6%	13.8%	9.2%	32.3%	50.0%	67.7%	36.9%	24.6%	15.4%	37.7%	36.2%	30.0%	30.8%	17.7%	6.2%	16.9%
GMT IC50 (μg/mL)	0.97	1.80	0.41	2.79	0.79	1.16	0.25	0.41	0.81	2.13	0.97	2.25	1.62	0.13	1.48	0.06	2.80
N332 Panel	22.5%	65.2%	20.2%	13.5%	44.9%	55.1%	75.3%	53.9%	36.0%	22.5%	55.1%	52.8%	43.8%	44.9%	25.8%	9.0%	24.7%
GMT IC50 (μg/mL)	0.97	1.80	0.41	2.79	0.71	0.95	0.17	0.41	0.81	2.13	0.97	2.25	1.62	0.13	1.48	0.06	2.80

Fig. S4. Breadth and potency of SHIV.5MUT-elicited V3-glycan bNAbs. Neutralization breadth and potency of the SHIV.5MUT-elicited V3-glycan bNAbs was assessed using a panel of 130 tier-2 viruses consisting of a 119-virus global panel (blue) plus 11 additional viruses (purple).

Viruses are separated by subtype, and sequence features including V1 length, N332/N334 glycan status, and V3 epitope sequence are shown. Neutralization titers are expressed as IC_{50} in µg/mL.

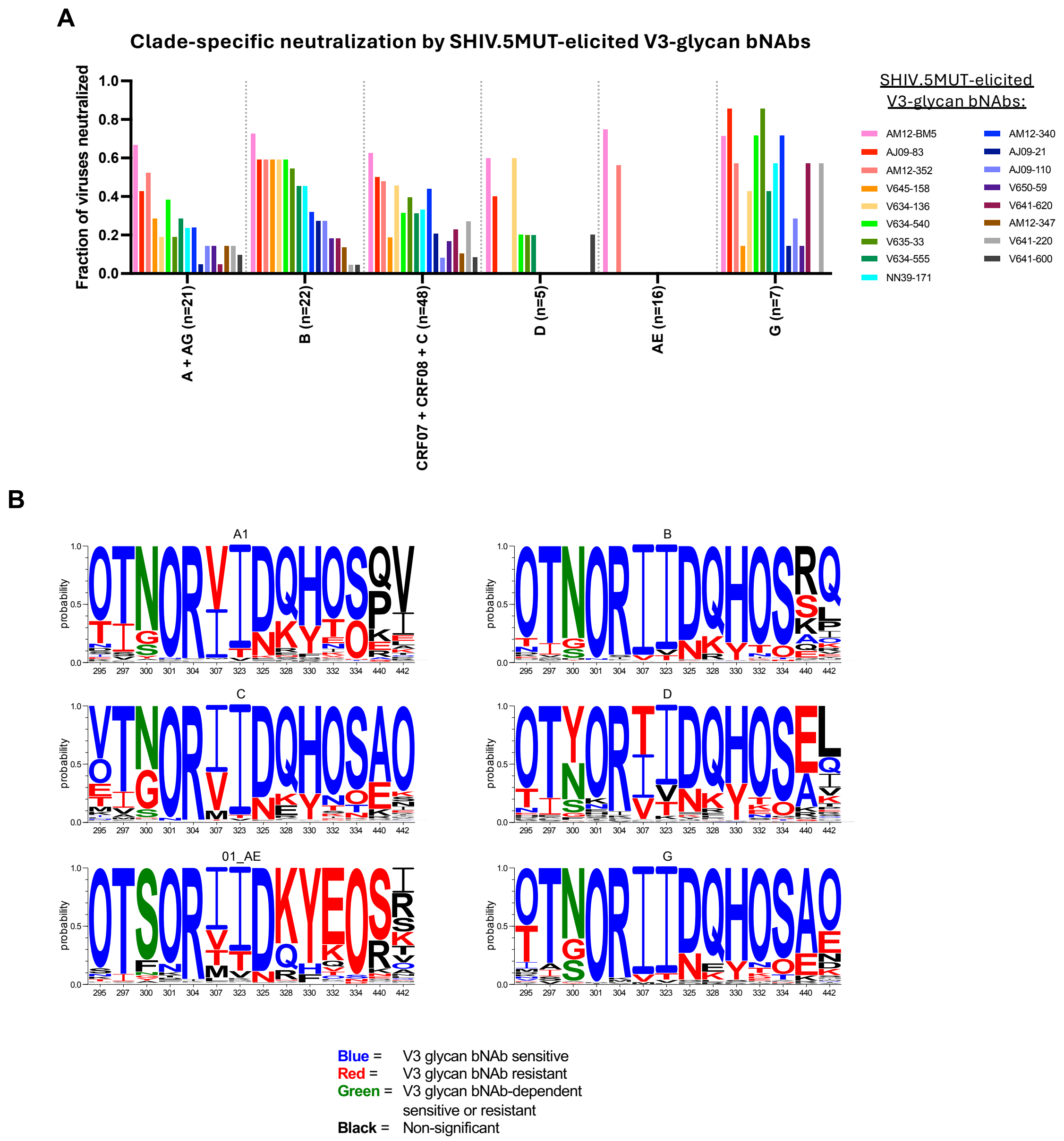

Fig. S5. Clade-specific breadth of SHIV.5MUT-elicited V3-glycan bNAbs. (A) Summary bar graph highlighting the fraction of heterologous viruses in each clade that is neutralized by the indicated antibody. CRF07 & CRF08 are clade C in Env, and CRF02 is clade A in Env, and hence these CRFs are grouped with the parental clades. **(B)** Sequence diversity at major V3-glycan bNAb epitope signature sites shown for six M-group clades (*21*). Logos were produced using the AnalyzeAlign tool on the Los Alamos HIV Database (www.hiv.lanl.gov) using the database alignment of M-group Envs.

A

mAb ID	MLV	Q23							CAP256SU				
		WT	D325N	R327K	H330Y	T334A	T334N	T303A	WT	D325A	H330Y	S334A	T303A
AM12-352	>50	0.09	6.99	>50	0.38	0.16	0.14	>50	0.15	>50	1.02	1.31	>50
AM12-340	>50	0.05	1.49	18.46	0.88	>50	>50	0.01	7.53	>50	>50	>50	1.63
AM12-347	>50	0.38	15.13	>50	>50	>50	>50	0.02	5.82	>50	>50	>50	3.33

B

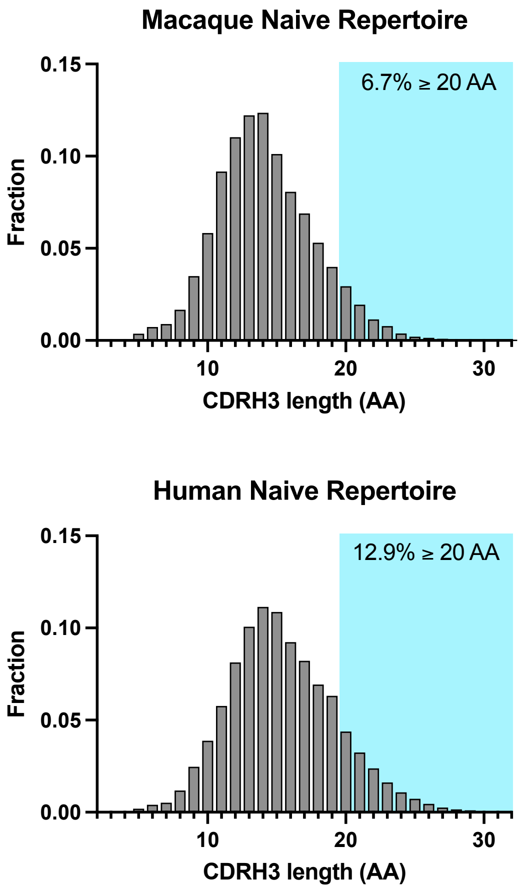

Fig. S6. Representatives of three AM12 V3-glycan bNAb sub-lineages exhibit diversity in epitope recognition. (A) Epitope mapping of representative members of three AM12 V3-glycan bNAb sub-lineages. Neutralization activity against a panel of Q23 and CAP256SU mutant viruses with mutations at key residues in the V3-glycan epitope is shown, with titers expressed as IC_{50} in µg/mL. **(B)** Distribution of CDRH3 lengths in naïve IgM^+ peripheral B cells in humans (top) and macaques (bottom), identified by bulk BCR repertoire sequencing. Sequences with CDRH3 ≥20 AA are highlighted. The human dataset was taken from ref (*52*), and sequences from outlier participant 1070 were excluded. The rhesus dataset was generated in this study and includes 2,172,207 IgM sequences from eight macaques.

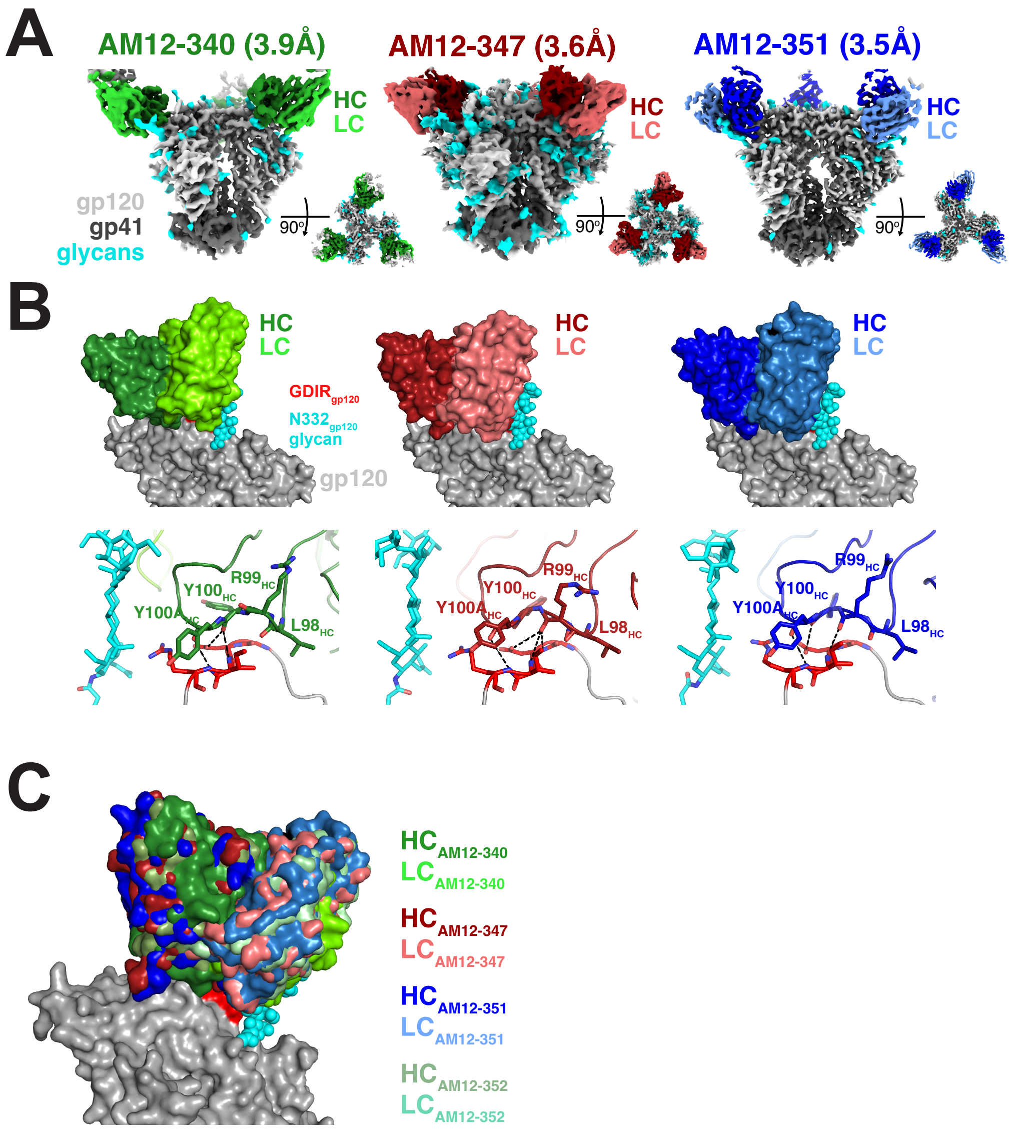

Fig. S7. Structural characterization of AM12-352 lineage members. (A) Cryo-EM maps of AM12-340 (left), AM12-347 (middle), and AM12-351 (right) bNAbs in complex with the 5MUT-3fill SOSIP. The heavy chain of each bNAb is shown in a darker shade, and the light chain is shown in a lighter shade. The N332 glycan is modeled as cyan spheres and the conserved GDIR peptide motif is red. **(B)** Top, surface representations of the V3-glycan bNAbs from **(A)** bound to gp120, with the N332 glycan and GDIR motif as depicted in **(A)**. Bottom, interfaces of the predicted interactions between the V3-glycan bNAbs and the GDIR motif. **(C)** Structural overlay of AM12 lineage members demonstrating nearly identical approach angles for binding to Env.

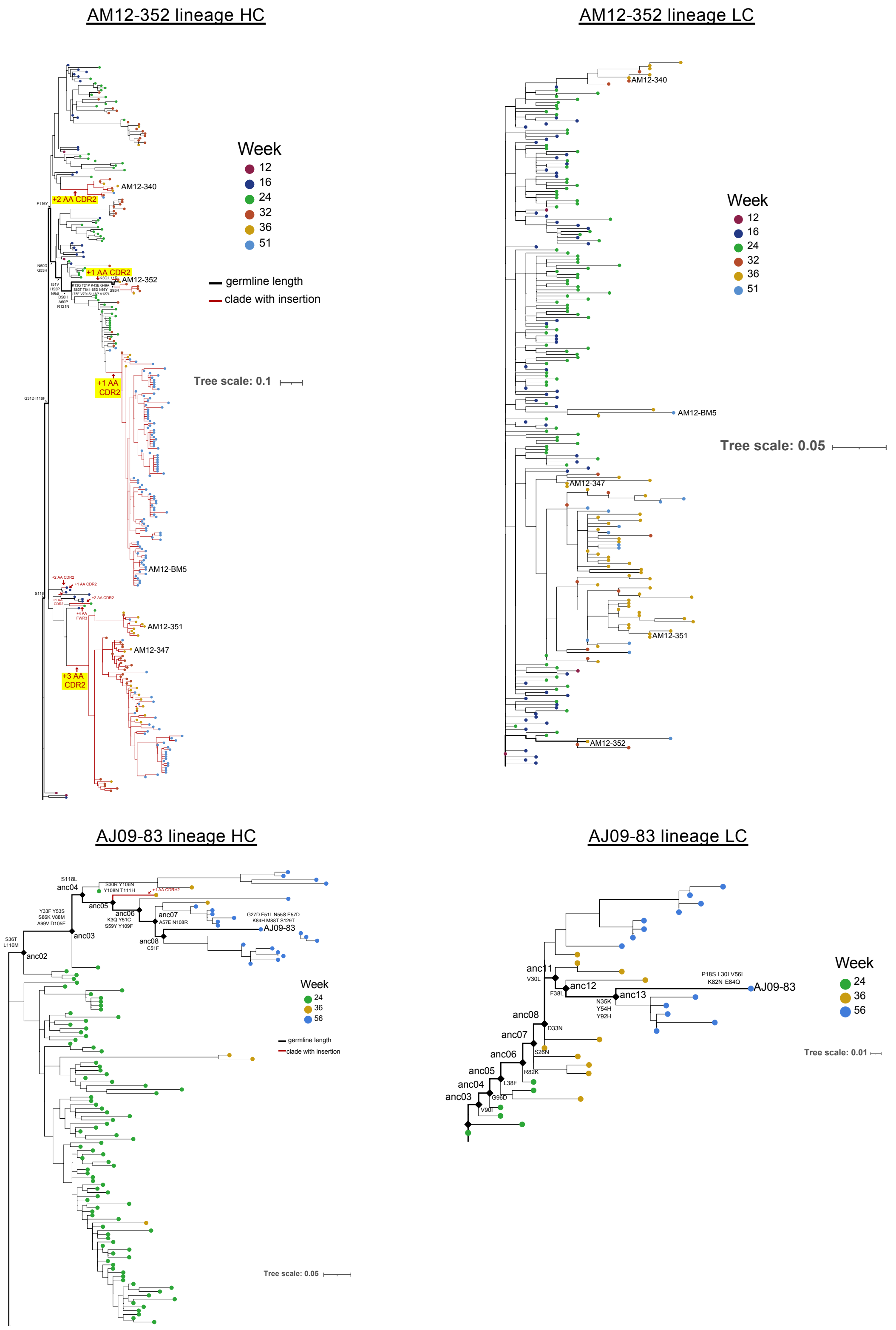

Fig. S8. Evolution of the AM12-352 and AJ09-83 bNAb lineages. Phylogenetic trees of heavy and light chain sequences derived from NGS and isolated mAbs are shown for two rhesus bNAb lineages whose UCAs did not bind to common V1 escape mutants **(Fig. 5F)**. Ancestors were

inferred at each node by analyzing longitudinal bulk BCR sequencing data using the SONAR computational analysis pipeline. Sequences are color coded according to their week of isolation and the position of mature bNAbs on the trees are shown. Branches in the heavy chain tree on which amino acid insertions occurred are indicated in red, with four particular CDRH2 insertions highlighted. The bold black line highlights intermediary nodes to the mature bNAb. The tree was constructed from heavy and light chain nucleotide sequences using IgPhyML and the scale bar indicates the number of estimated mutations per site.

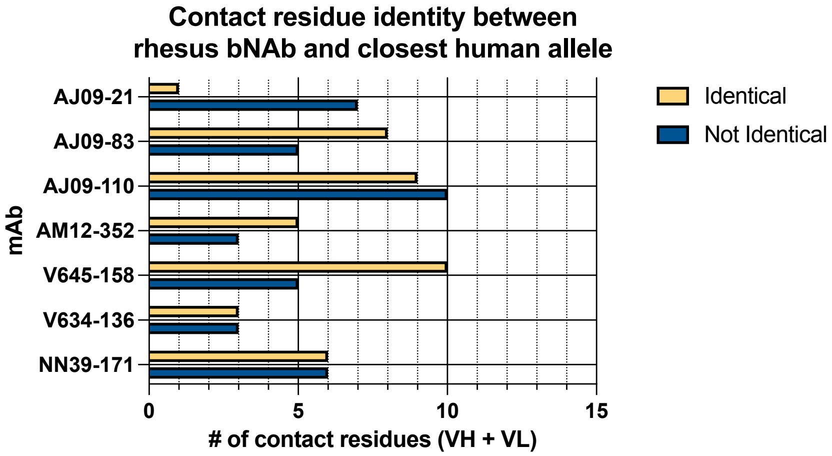

Fig. S9. SHIV.5MUT-elicited bNAbs share the majority of their contact residues with the closest human V allele. The number of contact residues that are (yellow) or are not (blue) identical in the closest human V_H and V_K alleles are shown for the indicated bNAbs.

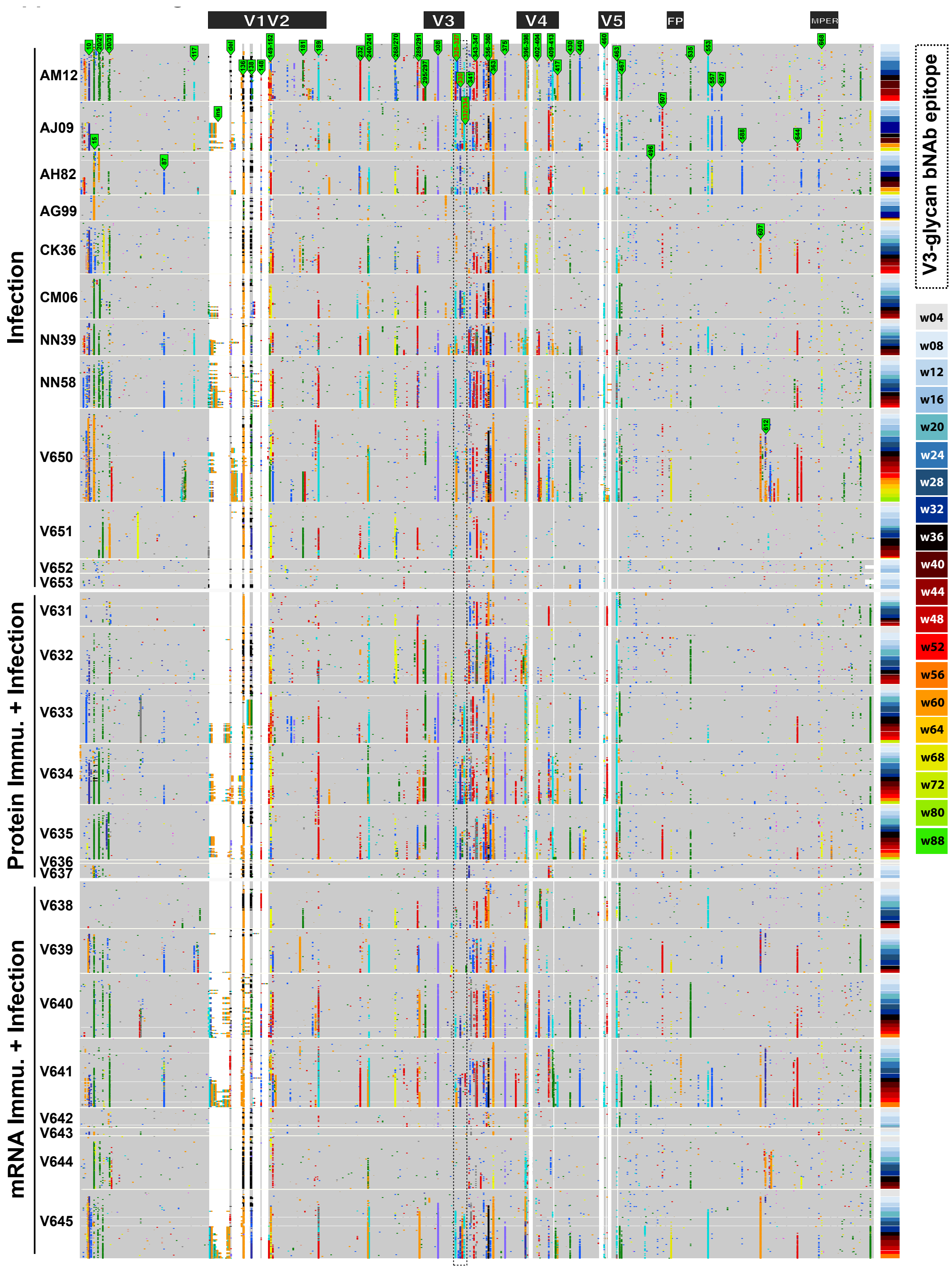

Fig. S10. Immune selection in V1 and V3 regions in SHIV.5MUT-infected rhesus macaques. A highlighter plot of longitudinal Env amino acid sequences obtained by single-genome sequencing of plasma viral RNA is shown for each macaque. Sequences are grouped first by

macaque (left) and then by timepoint (right). Each row represents a single sequence, depicted as a string of horizontal pixels with each pixel representing a single amino acid in the alignment. Sequences are shown for the entire Env ectodomain, with a schematic map depicted at top. A single alignment was generated from sequences of all SHIV.5MUT-infected macaques at all timepoints, such that the vertical "stripes" indicate mutations at the same site in different Envs. All sequences are aligned to the SHIV.5MUT Env sequence. Residues with substantial selection are indicated, with colors denoting different amino acids. Timepoints are color-coded on the right. Deletions are denoted by black boxes.

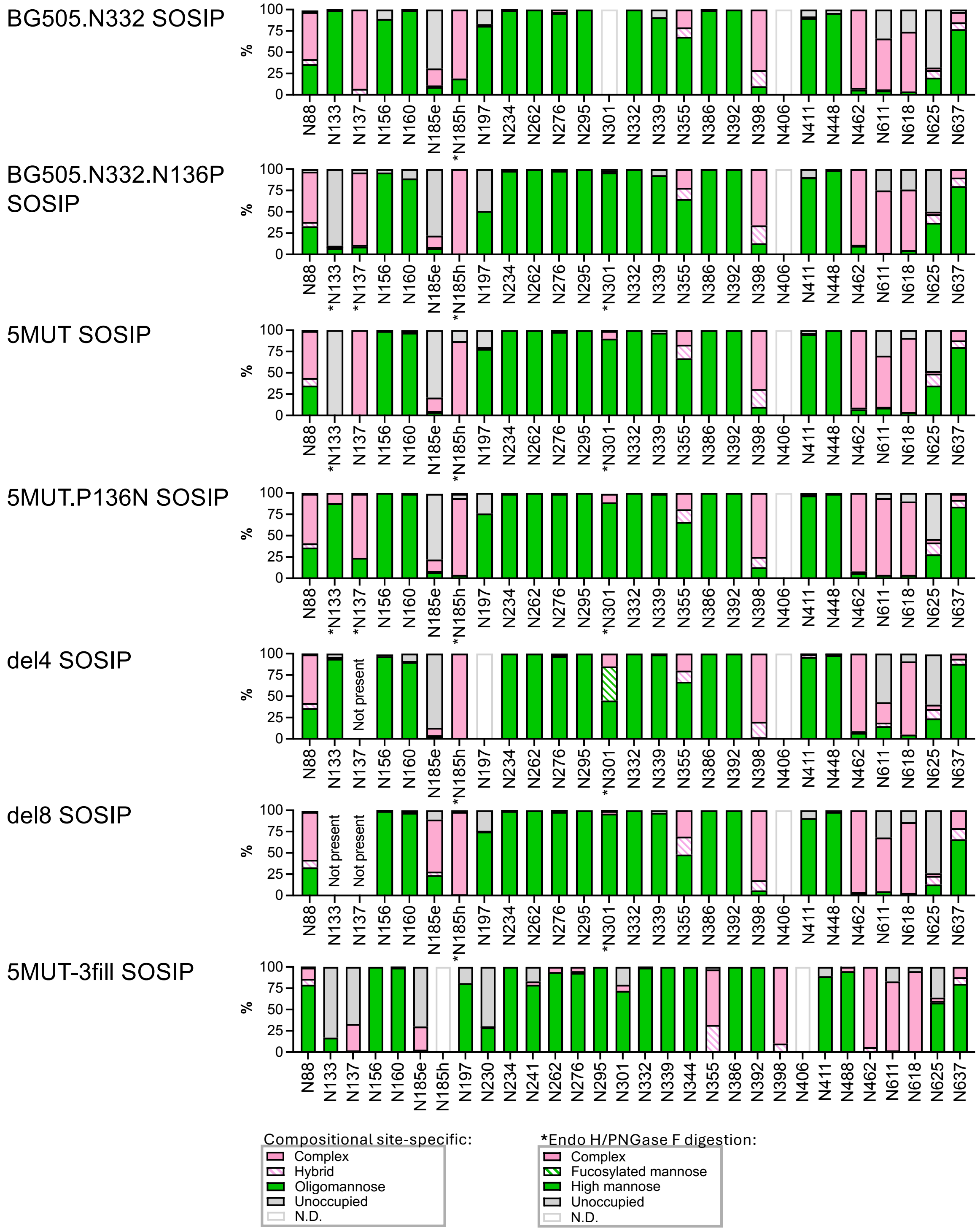

Fig. S11. Hypoglycosylation of position N133 in the 5MUT Env trimer. Mass spectroscopy-based site-specific glycan analysis of MD39-stabilized BG505.N332, BG505.N332.N136P, 5MUT, 5MUT.P136N, del4, del8, and 5MUT-3fill SOSIP proteins. Glycan compositions are grouped into their corresponding categories, with complex-type glycans displayed in pink, hybrid in hatched pink, oligomannose in green, and unoccupied in gray. For sites in which the site-specific data was deduced by employing glycosidase digestion by Endo H followed by PNGase F in O18

water (indicated by asterisks), the peptide modifications which correspond to distinct glycan types are grouped into high mannose in green, fucosylated mannose in hatched green, complex in pink, and unoccupied in gray. Glycan sites that could not be determined are denoted as “N.D.”

BG505 SOSIP	N88	N133	N137	N156	N160	N185e	N185h	N197	N234	N262	N276	N295	N301	N332	N339	N355	N386	N392	N398	N406	N411	N448	N468	N611	N618	N625	N637
**Oligomannose**	36	99	0	89	99	9	0	81	99	100	96	100		100	91	68	99	100	10		90	96	6	5	4	20	77
**High Mannose**	0						19	0						0							0						
M9Glc	0	0	0	0	0	0		0	1	1	0	0		0	0	0	0	0	0		0	0	0	0	0	0	0
M9	0	24	0	55	40	0		43	59	65	0	83		84	48	0	86	10	0		1	43	0	0	0	0	2
M8	0	48	0	28	32	0		26	24	27	38	16		12	29	3	10	79	1		21	41	0	0	0	2	18
M7	1	17	0	6	10	1		4	9	5	26	0		2	6	9	2	8	0		21	8	1	0	0	3	29
M6	5	7	0	1	7	0	19	3	2	2	12	0	N.D.	1	4	6	0	2	1	N.D.	18	2	0	1	0	5	15
M5	30	2	0	0	7	7		6	3	1	18	0		1	3	49	0	1	8		29	1	4	4	3	10	11
M4	0	0	0	0	2	0		0	1	0	2	0		0	1	0	0	0	1		0	0	0	0	0	0	2
M3	0	0	0	0	0	0		0	0	0	1	0		0	0	0	0	0	0		0	0	0	0	0	0	0
FM	0	0	0	0	0	0		0	0	0	0	0		0	0	0	0	0	0		0	0	0	0	1	0	0
**Hybrid/FM**	6	1	7	0	0	2	0	0	0	0	1	0		0	0	12	0	0	19		2	0	2	1	0	9	8
Hybrid	6	0	7	0	0	1	0	0	0	0	1	0	N.D.	0	0	6	0	0	1	N.D.	2	0	0	0	0	9	5
Fhybrid	0	1	0	0	0	1		0	0	0	0	0		0	0	6	0	0	18		0	0	1	0	0	0	3
**Complex**	55	0	93	0	1	20	81	2	0	0	3	0		0	0	21	1	0	71		0	0	93	60	70	3	12
HexNAc(3)(x)	9	0	0	0	0	0		0	0	0	1	0		0	0	1	0	0	0		0	0	0	1	0	2	1
HexNAc(3)(F)(x)	0	0	19	0	0	4		0	0	0	0	0		0	0	9	0	0	15		0	0	8	2	1	0	3
HexNAc(4)(x)	16	0	0	0	0	0		0	0	0	0	0		0	0	0	0	0	0		0	0	1	0	1	0	0
HexNAc(4)(F)(x)	6	0	59	0	0	11		2	0	0	0	0		0	0	5	0	0	32		0	0	50	7	36	1	8
HexNAc(5)(x)	12	0	0	0	0	0	81	0	0	0	0	0	N.D.	0	0	0	0	0	0	N.D.	0	0	1	0	0	0	0
HexNAc(5)(F)(x)	11	0	16	0	0	4		0	0	0	0	0		0	0	5	0	0	23		0	0	26	47	24	0	0
HexNAc(6+)(x)	0	0	0	0	0	0		0	0	0	0	0		0	0	0	0	0	0		0	0	0	0	0	0	0
HexNAc(6+)(F)(x)	1	0	0	0	0	0		0	0	0	0	0		0	0	0	0	0	0		0	0	5	2	7	0	0
Core	0	0	0	0	0	0		0	0	0	1	0		0	0	0	1	0	0		0	0	1	0	0	0	0
**Unoccupied**	2	0	0	11	0	69	0	17	1	0	0	0		0	9	0	0	0	0		8	4	0	34	26	68	3

5MUT SOSIP	N88	N133	N137	N156	N160	N185e	N185h	N197	N234	N262	N276	N295	N301	N332	N339	N355	N386	N392	N398	N406	N411	N448	N468	N611	N618	N625	N637
**Oligomannose**	35	0	0	99	98	4	0	78	100	100	98	100	0	100	97	67	100	100	10		94	99	7	9	4	35	80
**High Mannose**	0	0	0				0	0					90	0							0						
M9Glc	0		0	0	0	0		0	3	1	0	0		0	0	0	0	0	0		0	2	0	0	0	0	0
M9	0		0	1	51	0		47	26	69	0	84		90	57	0	74	1	0		1	27	0	0	0	1	2
M8	0		0	12	29	0		22	50	26	45	16		8	30	5	17	89	1		27	61	0	0	0	4	15
M7	1		0	40	8	0		4	12	2	29	0		1	5	9	6	7	1		22	6	1	0	0	8	28
M6	2	0	0	19	5	0	0	2	6	1	9	0	90	0	3	8	2	1	0	N.D.	17	2	0	0	0	8	18
M5	31		0	26	4	3		3	1	0	15	0		0	2	44	1	1	7		26	1	6	9	3	15	16
M4	1		0	0	1	0		0	2	0	1	0		0	0	1	0	0	1		0	0	0	0	0	0	1
M3	0		0	0	0	0		0	0	0	0	0		0	0	0	0	0	0		0	0	0	0	0	0	0
FM	0		0	0	0	0		0	0	0	0	0		0	0	0	0	0	0		0	0	0	0	1	0	0
**Hybrid/FM**	9	0	0	1	0	1	1	0	0	0	1	0	0	0	0	15	0	0	21		1	0	2	1	0	14	8
Hybrid	8	0	0	1	0	0	1	0	0	0	1	0	0	0	0	7	0	0	1	N.D.	1	0	0	0	0	14	6
Fhybrid	0		0	0	0	0		0	0	0	0	0		0	0	8	0	0	20		0	0	2	1	0	0	2
**Complex**	55	0	100	0	1	16	87	2	0	0	1	0	9	0	0	17	0	0	69		0	0	91	60	87	3	12
HexNAc(3)(x)	10		0	0	0	0		0	0	0	0	0		0	0	1	0	0	0		0	0	0	0	0	2	1
HexNAc(3)(F)(x)	1		1	0	0	2		0	0	0	0	0		0	0	7	0	0	10		0	0	5	2	2	0	2
HexNAc(4)(x)	20		0	0	0	0		0	0	0	0	0		0	0	0	0	0	0		0	0	0	0	0	0	0
HexNAc(4)(F)(x)	7		1	0	0	10		1	0	0	0	0		0	0	5	0	0	42		0	0	55	5	41	1	9
HexNAc(5)(x)	10	0	0	0	0	0	87	0	0	0	0	0	9	0	0	0	0	0	0	N.D.	0	0	0	0	0	0	0
HexNAc(5)(F)(x)	6		0	0	0	4		0	0	0	0	0		0	0	3	0	0	17		0	0	26	51	34	0	1
HexNAc(6+)(x)	0		0	0	0	0		0	0	0	0	0		0	0	0	0	0	0		0	0	0	0	0	0	0
HexNAc(6+)(F)(x)	0		98	0	0	0		0	0	0	0	0		0	0	0	0	0	0		0	0	3	2	10	0	0
Core	0		0	0	0	0		0	0	0	1	0		0	0	0	0	0	0		0	0	1	0	0	0	0
**Unoccupied**	1	100	0	0	2	79	13	20	0	0	0	0	1	0	3	0	0	0	0		4	0	0	30	9	48	0

BG505 N136P SOSIP	N88	N133	N137	N156	N160	N185e	N185h	N197	N234	N262	N276	N295	N301	N332	N339	N355	N386	N392	N398	N406	N411	N448	N468	N611	N618	N625	N637
**Oligomannose**	33	0	0	96	89	7	0	51	98	100	98	100	0	100	92	65	100	100	13		89	99	10	2	5	37	80
**High Mannose**	0	16	57	0			0	0					95	0							0						
M9Glc	0			0	0	0		0	0	3	0	0		0	0	0	0	0	0		0	0	0	0	0	0	0
M9	0			56	22	0		32	72	1	1	87		77	60	0	89	6	0		0	50	0	0	0	1	0
M8	1			22	56	0		18	19	90	52	13		15	21	6	6	84	0		38	47	1	0	0	7	16
M7	2			14	7	1		1	2	3	18	0		3	6	13	2	7	0		20	1	1	1	0	0	30
M6	2	16	57	3	4	0	0	0	4	2	11	0	95	3	3	7	2	1	1	N.D.	6	0	0	1	0	9	18
M5	27			2	0	6		0	2	1	15	0		1	2	38	1	1	11		25	1	8	0	5	20	14
M4	1			0	0	0		0	0	0	2	0		0	1	1	0	0	1		0	0	0	0	0	1	1
M3	0			0	0	0		0	0	0	1	0		0	0	1	0	0	0		0	0	0	0	0	0	0
FM	0			0	0	0		0	0	0	0	0		0	0	0	0	0	0		0	0	0	0	0	0	0
**Hybrid/FM**	5	0	16	0	0	1	1	0	0	0	1	0	0	0	0	13	0	0	21		1	0	1	0	0	10	10
Hybrid	5	0	16	0	0	0	1	0	0	0	1	0	0	0	0	7	0	0	1	N.D.	1	0	0	0	0	9	6
Fhybrid	0			0	0	1		0	0	0	0	0		0	0	6	0	0	20		0	0	1	0	0	0	4
**Complex**	58	4	22	0	0	14	99	0	1	0	1	0	2	0	0	22	0	0	66		0	0	89	73	71	4	10
HexNAc(3)(x)	14			0	0	0		0	0	0	0	0		0	0	1	0	0	0		0	0	0	0	0	3	1
HexNAc(3)(F)(x)	0			0	0	3		0	0	0	0	0		0	0	9	0	0	10		0	0	5	2	3	0	1
HexNAc(4)(x)	21			0	0	0		0	0	0	0	0		0	0	0	0	0	0		0	0	0	2	0	0	0
HexNAc(4)(F)(x)	7			0	0	7		0	0	0	0	0		0	0	6	0	0	44		0	0	57	2	32	1	7
HexNAc(5)(x)	10	4	22	0	0	0	99	0	0	0	0	0	2	0	0	0	0	0	0	N.D.	0	0	0	2	0	0	0
HexNAc(5)(F)(x)	6			0	0	3		0	0	0	0	0		0	0	4	0	0	12		0	0	26	21	27	0	0
HexNAc(6+)(x)	0			0	0	0		0	0	0	0	0		0	0	0	0	0	0		0	0	0	44	0	0	0
HexNAc(6+)(F)(x)	0			0	0	0		0	0	0	0	0		0	0	0	0	0	0		0	0	0	0	9	0	0
Core	0			0	0	0		0	1	0	0	0		0	0	1	0	0	0		0	0	0	0	1	0	0
**Unoccupied**	3	81	5	4	11	78	0	49	2	0	0	0	2	0	7	0	0	0	0		9	1	0	24	24	50	1

5MUT P136N SOSIP	N88	N133	N137	N156	N160	N185e	N185h	N197	N234	N262	N276	N295	N301	N332	N339	N355	N386	N392	N398	N406	N411	N448	N468	N611	N618	N625	N637
**Oligomannose**	36	0	0	100	99	7	0	76	99	100	99	100	0	100	98	65	100	100	13		97	99	6	4	5	29	84
**High Mannose**	0	86	24	0			4	0					89	0							0						
M9Glc	0			0	0	0		0	0	1	0	0		0	0	0	0	0	0		0	0	0	0	0	0	0
M9	0			23	48	0		42	63	63	0	84		86	58	0	77	12	0		1	50	0	0	0	0	3
M8	0			28	34	0		25	23	27	45	16		14	31	4	15	78	0		23	40	0	0	0	3	31
M7	1			30	8	1		4	7	5	27	1		0	5	12	5	8	1		24	6	0	0	0	6	16
M6	4	86	24	10	5	0	4	2	4	3	11	0	89	0	2	9	2	1	0	N.D.	20	2	0	0	0	7	22
M5	30			10	4	6		3	1	1	13	0		0	1	39	1	0	11		30	1	5	3	3	13	10
M4	1			0	1	0		0	0	0	1	0		0	0	1	0	0	1		0	0	0	0	0	0	2
M3	0			0	0	0		0	0	0	0	0		0	0	0	0	0	0		0	0	0	0	0	0	0
FM	0			0	0	0		0	0	0	0	0		0	0	0	0	0	0		0	0	0	0	2	0	0
**Hybrid/FM**	5	0	1	0	0	1	1	0	0	0	1	0	0	0	0	15	0	0	12		1	0	2	0	0	14	8
Hybrid	5	0	1	0	0	0	1	0	0	0	1	0	0	0	0	7	0	0	0	N.D.	1	0	0	0	0	14	5
Fhybrid	1			0	0	1		0	0	0	0	0		0	0	9	0	0	12		0	0	1	0	0	0	2
**Complex**	58	12	75	0	0	14	90	0	1	0	1	0	10	0	0	19	0	0	75		0	0	92	90	86	4	7
HexNAc(3)(x)	6			0	0	0		0	0	0	0	0		0	0	1	0	0	0		0	0	0	0	0	2	0
HexNAc(3)(F)(x)	1			0	0	3		0	0	0	0	0		0	0	8	0	0	25		0	0	3	1	2	0	2
HexNAc(4)(x)	19			0	0	0		0	0	0	0	0		0	0	0	0	0	0		0	0	0	2	3	0	0
HexNAc(4)(F)(x)	10			0	0	9		0	0	0	0	0		0	0	6	0	0	37		0	0	56	2	25	1	4
HexNAc(5)(x)	15	12	75	0	0	0	90	0	0	0	0	0	10	0	0	0	0	0	0	N.D.	0	0	0	2	0	0	0
HexNAc(5)(F)(x)	7			0	0	3		0	0	0	0	0		0	0	4	0	0	13		0	0	30	18	44	0	0
HexNAc(6+)(x)	1			0	0	0		0	0	0	0	0		0	0	0	0	0	0		0	0	0	63	0	0	0
HexNAc(6+)(F)(x)	0			0	0	0		0	0	0	0	0		0	0	0	0	0	0		0	0	3	3	11	0	0
Core	0			0	0	0		0	1	0	0	0		0	0	0	0	0	0		0	0	0	0	0	0	0
**Unoccupied**	1	0	0	0	1	77	5	24	0	0	0	0	0	0	1	0	0	0	0		2	1	0	6	10	54	1

5MUT del8 SOSIP	N88	N133	N137	N156	N160	N185e	N185h	N197	N234	N262	N276	N295	N301	N332	N339	N355	N386	N392	N398	N406	N411	N448	N468	N611	N618	N625	N637
**Oligomannose**	33			99	97	24	0	75	99	100	98	100	0	100	97	48	100	100	6		91	98	3	5	3	13	66
**High Mannose**	0			0			1	0					96	0							0						
M9Glc	0			0	0	0		0	1	1	0	0		0	0	0	0	0	0		0	0	0	0	0	0	0
M9	0			3	51	0		46	64	59	0	31		83	52	0	73	8	0		0	48	0	0	0	0	1
M8	0			14	29	1		22	22	25	45	69		17	33	1	15	83	0		32	41	0	0	0	0	15
M7	1			59	8	1		3	5	7	27	0		0	6	4	6	7	0		34	7	0	0	0	1	6
M6	3			12	5	1	1	1	4	5	11	0	96	0	3	4	4	1	0	N.D.	21	1	0	0	0	4	21
M5	29			11	3	20		2	3	2	13	0		0	2	38	2	1	5		2	1	2	5	2	8	21
M4	1			0	1	0		0	1	1	1	0		0	1	1	0	0	0		2	0	0	0	0	0	1
M3	0			0	0	0		0	0	0	0	0		0	0	0	0	0	0		0	0	0	0	0	0	0
FM	0			0	0	0		0	0	0	0	0		0	0	0	0	0	0		0	0	0	0	1	0	0
**Hybrid/FM**	9	Not present		1	0	4	2	0	0	0	1	0	0	0	0	20	0	0	12		0	0	1	0	0	10	13
Hybrid	9			1	0	1	2	0	0	0	1	0	0	0	0	9	0	0	0	N.D.	0	0	0	0	0	10	8
Fhybrid	0			0	0	3		0	0	0	0	0		0	0	11	0	0	12		0	0	1	0	0	0	5
**Complex**	56			0	1	61	98	1	1	0	1	0	3	0	0	31	0	0	81		0	0	96	63	83	3	21
HexNAc(3)(x)	8			0	0	0		0	0	0	0	0		0	0	0	0	0	0		0	0	0	0	0	2	1
HexNAc(3)(F)(x)	1			0	0	10		0	0	0	0	0		0	0	14	0	0	27		0	0	4	2	2	0	6
HexNAc(4)(x)	19			0	0	0		0	0	0	0	0		0	0	0	0	0	0		0	0	1	0	3	0	0
HexNAc(4)(F)(x)	7			0	0	33		1	0	0	0	0		0	0	10	0	0	29		0	0	51	6	35	1	13
HexNAc(5)(x)	15			0	0	0	98	0	0	0	0	0	3	0	0	0	0	0	0	N.D.	0	0	0	0	0	0	0
HexNAc(5)(F)(x)	7			0	0	16		0	0	0	0	0		0	0	6	0	0	25		0	0	32	50	35	0	1
HexNAc(6+)(x)	0			0	0	0		0	0	0	0	0		0	0	0	0	0	0		0	0	0	0	0	0	0
HexNAc(6+)(F)(x)	0			0	0	1		0	0	0	0	0		0	0	0	0	0	0		0	0	6	4	8	0	0
Core	0			0	0	0		0	1	0	1	0		0	0	0	0	0	0		0	0	0	0	0	0	0
**Unoccupied**	1			0	2	11	0	25	1	0	0	0	1	0	3	0	0	0	0		9	2	0	32	14	74	0

5MUT del4 SOSIP	N88	N133	N137	N156	N160	N185e	N185h	N197	N234	N262	N276	N295	N301	N332	N339	N355	N386	N392	N398	N406	N411	N448	N468	N611	N618	N625	N637
**Oligomannose**	36	94		97	90	3	0		100	100	97	100	0	100	99	67	100	100	2		96	98	7	15	5	24	88
**High Mannose**	0			0			0		0				45	0							0						
M9Glc	0	0		0	0	0			1	1	0	0		1	0	0	0	0	0		0	0	0	0	0	0	0
M9	0	1		1	44	0			57	62	0	88		62	64	0	80	15	0		1	43	0	0	0	0	1
M8	0	18		5	34	0			31	25	35	12		36	32	3	15	78	0		24	41	0	0	0	2	20
M7	0	33		59	7	0			6	6	22	1		1	2	8	3	6	0		15	8	0	0	0	6	37
M6	6	23		14	3	0	0	N.D.	3	3	15	0	45	0	1	7	2	1	0	N.D.	24	3	0	0	0	5	19
M5	28	19		18	2	3			2	1	24	0		0	0	47	0	0	2		32	1	6	15	3	10	10
M4	1	0		0	0	0			0	1	2	0		0	0	1	0	0	0		0	1	0	0	0	0	1
M3	0	0		0	0	0			0	0	0	0		0	0	0	0	0	0		0	0	0	0	0	0	0
FM	0	0		0	0	0			0	0	0	0		0	0	0	0	0	0		0	0	0	0	2	0	0
**Hybrid/FM**	6	1	Not present	2	0	1	0		0	0	1	0	40	0	0	13	0	0	18		3	0	2	4	0	11	6
Hybrid	6	1		2	0	0	0	N.D.	0	0	1	0	40	0	0	6	0	0	0	N.D.	2	0	0	0	0	11	4
Fhybrid	0	0		0	0	1			0	0	0	0		0	0	7	0	0	18		1	0	2	4	0	0	2
**Complex**	57	1		0	1	9	100		0	0	2	0	15	0	0	20	0	0	80		1	1	91	24	86	5	6
HexNAc(3)(x)	7	0		0	0	0			0	0	0	0		0	0	1	0	0	0		0	0	0	2	0	2	0
HexNAc(3)(F)(x)	1	0		0	0	2			0	0	0	0		0	0	6	0	0	21		0	0	7	1	3	0	1
HexNAc(4)(x)	19	0		0	0	0			0	0	0	0		0	0	0	0	0	0		0	0	0	0	2	1	0
HexNAc(4)(F)(x)	7	0		0	0	3			0	0	0	0		0	0	4	0	0	31		0	0	44	3	23	2	4
HexNAc(5)(x)	15	0		0	0	0	100	N.D.	0	0	0	0	15	0	0	0	0	0	0	N.D.	0	0	0	0	0	0	0
HexNAc(5)(F)(x)	8	0		0	0	2			0	0	0	0		0	0	2	0	0	24		0	0	31	16	29	1	0
HexNAc(6+)(x)	0	0		0	0	0			0	0	0	0		0	0	0	0	0	0		0	0	0	0	0	0	0
HexNAc(6+)(F)(x)	0	0		0	0	0			0	0	0	0		0	0	0	0	0	4		0	0	7	1	29	0	0
Core	0	1		0	1	1			0	0	1	0		0	0	8	0	0	0		0	1	1	2	0	0	0
**Unoccupied**	1	4		0	9	87	0		0	0	0	0	0	0	1	0	0	0	0		0	1	0	57	9	59	0

5MUT 3fill SOSIP	N88	N133	N137	N156	N160	N185e	N185h	N197	N230	N234	N241	N262	N276	N295	N301	N332	N339	N344	N355	N386	N392	N398	N406	N411	N488	N462	N611	N618	N625	N637
**Oligomannose**	79	17	0	100	99	0		81	29	100	79	94	94	100	72	99	100	100	1	100	100	0		89	95	0	0	0	58	80
**High Mannose**	0							0																0						
M9Glc	0	0	0	0	0	0		0	0	0	0	2	0	0	0	0	1	0	0	0	0	0		0	0	0	0	0	0	0
M9	0	0	0	7	28	0		54	11	70	24	39	0	81	0	77	4	77	0	71	24	0		0	69	0	0	0	1	2
M8	2	0	0	6	44	0		25	7	24	19	31	27	19	18	15	81	22	0	21	72	0		58	19	0	0	0	6	20
M7	5	0	0	68	12	0		0	5	4	20	7	30	0	15	4	12	1	0	6	1	0		16	4	0	0	0	19	31
M6	33	17	0	8	8	0	N.D.	0	3	2	9	11	14	0	5	2	1	0	0	0	2	0	N.D.	15	1	0	0	0	14	26
M5	39	0	0	10	6	0		2	1	0	4	3	22	0	34	1	2	0	0	1	1	0		0	0	0	0	0	18	0
M4	1	0	0	0	2	0		0	2	0	3	0	1	0	0	0	0	0	0	0	0	0		0	0	0	0	0	0	1
M3	0	0	0	0	0	0		0	0	0	2	0	1	0	0	0	0	0	1	0	0	0		0	0	0	0	0	0	0
FM	0	0	0	0	0	0		0	0	0	0	0	0	0	0	0	0	0	0	0	0	0		0	0	0	0	0	0	0
**Hybrid/FM**	7	0	2	0	0	3		0	0	0	0	0	2	0	0	0	0	0	31	0	0	10		0	0	6	2	0	2	9
Hybrid	7	0	0	0	0	0	N.D.	0	0	0	0	0	2	0	0	0	0	0	1	0	0	0	N.D.	0	0	0	0	0	2	5
Fhybrid	0	0	2	0	0	3		0	0	0	0	0	0	0	0	0	0	0	30	0	0	10		0	0	6	2	0	0	4
**Complex**	13	0	31	0	1	27		0	1	0	4	6	5	0	7	1	0	0	66	0	0	90		0	5	94	81	95	4	12
HexNAc(3)(x)	4	0	0	0	0	1		0	0	0	0	0	1	0	1	0	0	0	6	0	0	2		0	0	1	4	0	1	2
HexNAc(3)(F)(x)	0	0	2	0	0	6		0	0	0	0	0	0	0	0	0	0	0	25	0	0	47		0	0	11	10	5	0	0
HexNAc(4)(x)	6	0	0	0	0	0		0	0	0	0	0	1	0	0	0	0	0	0	0	0	0		0	0	0	0	0	0	0
HexNAc(4)(F)(x)	1	0	6	0	0	12		0	0	0	0	0	1	0	0	0	0	0	20	0	0	31		0	0	57	11	53	2	8
HexNAc(5)(x)	2	0	0	0	0	0	N.D.	0	0	0	0	0	0	0	0	0	0	0	0	0	0	0	N.D.	0	0	0	0	0	0	0
HexNAc(5)(F)(x)	1	0	2	0	0	6		0	0	0	0	0	0	0	0	0	0	0	13	0	0	7		0	0	24	56	26	0	1
HexNAc(6+)(x)	0	0	0	0	0	0		0	0	0	0	0	0	0	0	0	0	0	0	0	0	0		0	0	0	0	0	0	0
HexNAc(6+)(F)(x)	0	0	0	0	0	0		0	0	0	0	0	0	0	0	0	0	0	1	0	0	0		0	0	1	0	6	0	0
Core	0	0	21	0	0	3		0	0	0	2	6	1	0	6	1	0	0	0	0	0	3		0	5	1	0	6	0	0
**Unoccupied**	1	83	67	0	0	69		19	70	0	17	0	0	0	21	0	0	0	3	0	0	0		11	1	0	17	5	36	0

Fig. S12. Compositional site-specific glycan analysis of various BG505- and 5MUT-derived trimers. Quantification of site-specific occupancy and compositional glycan analysis of MD39-stabilized BG505.N332, BG505.N332.N136P, 5MUT, 5MUT.P136N, del4, del8, and 5MUT-3fill SOSIP proteins. The datasets show the glycoforms found at each potential N-linked glycosylation site (PNGS). Data for oligomannose glycans are shaded in green, hybrid-type glycans in orange, fully processed complex-type glycans in magenta, and the absence of a glycan from some PNGS in gray. Oligomannose-type glycans are categorized according to the number of mannose residues

present, hybrid-type glycans by the presence/absence of fucose, and complex-type glycans by the number of processed antenna and the presence/absence of fucose. For further information see **Methods**. Data that was obtained using Endo H and PNGase F in O18 water cannot be allocated into the above categories. They are merged to cover all oligomannose/hybrid compositions or complex-type glycans.

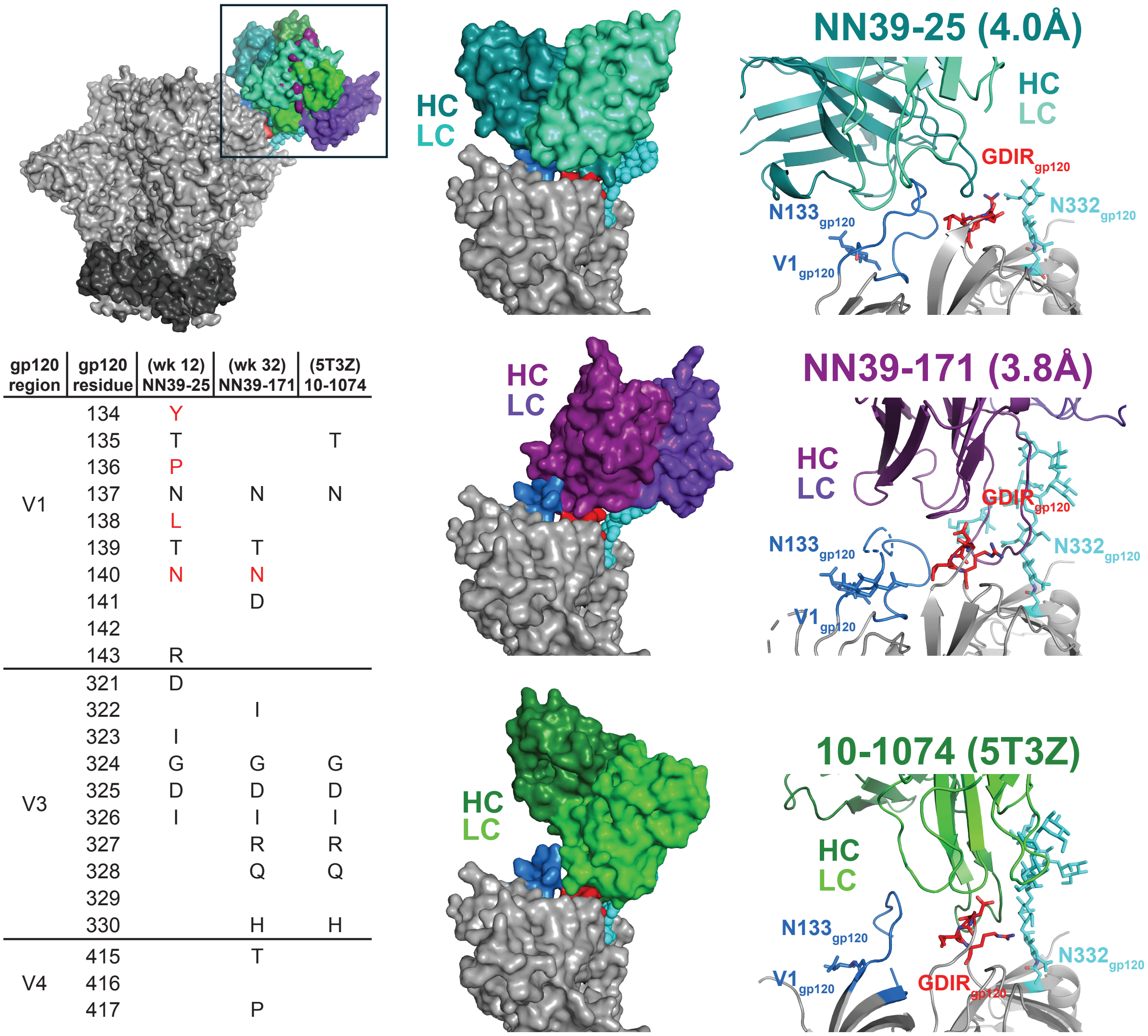

gp120 region	gp120 residue	(wk 12) NN39-25	(wk 32) NN39-171	(5T3Z) 10-1074
V1	134	Y		
	135	T		T
	136	P		
	137	N	N	N
	138	L		
	139	T	T	
	140	N	N	
	141		D	
	142			
	143	R		
V3	321	D		
	322		I	
	323	I		
	324	G	G	G
	325	D	D	D
	326	I	I	I
	327		R	R
	328		Q	Q
	329			
	330		H	H
V4	415		T	
	416			
	417		P	

Fig. S13. Comparative structural analysis of SHIV.5MUT-induced V1- and V3-directed neutralizing antibodies. Cryo-EM maps of two SHIV.5MUT-induced neutralizing antibodies (NN39-25 and NN39-171) in complex with 5MUT-3fill SOSIP, overlaid with a previously-published crystal structure of the human V3-glycan bNAb 10-1074 in complex with BG505 SOSIP. The heavy chain of each antibody is shown in a darker shade, while the light chain is shown in a lighter shade. The N332$_{gp120}$ glycan is shown in cyan, the conserved GDIR peptide motif in red, and the V1 loop (including the N133$_{gp120}$ glycan) in dark blue. NN39-25 exhibits strong V1 loop dependence, whereas NN39-171 and 10-1074 bNAbs bind predominantly to the V3 loop. Contact residues are shown in the table, with red font indicating the four residues differentiating 5MUT from BG505.

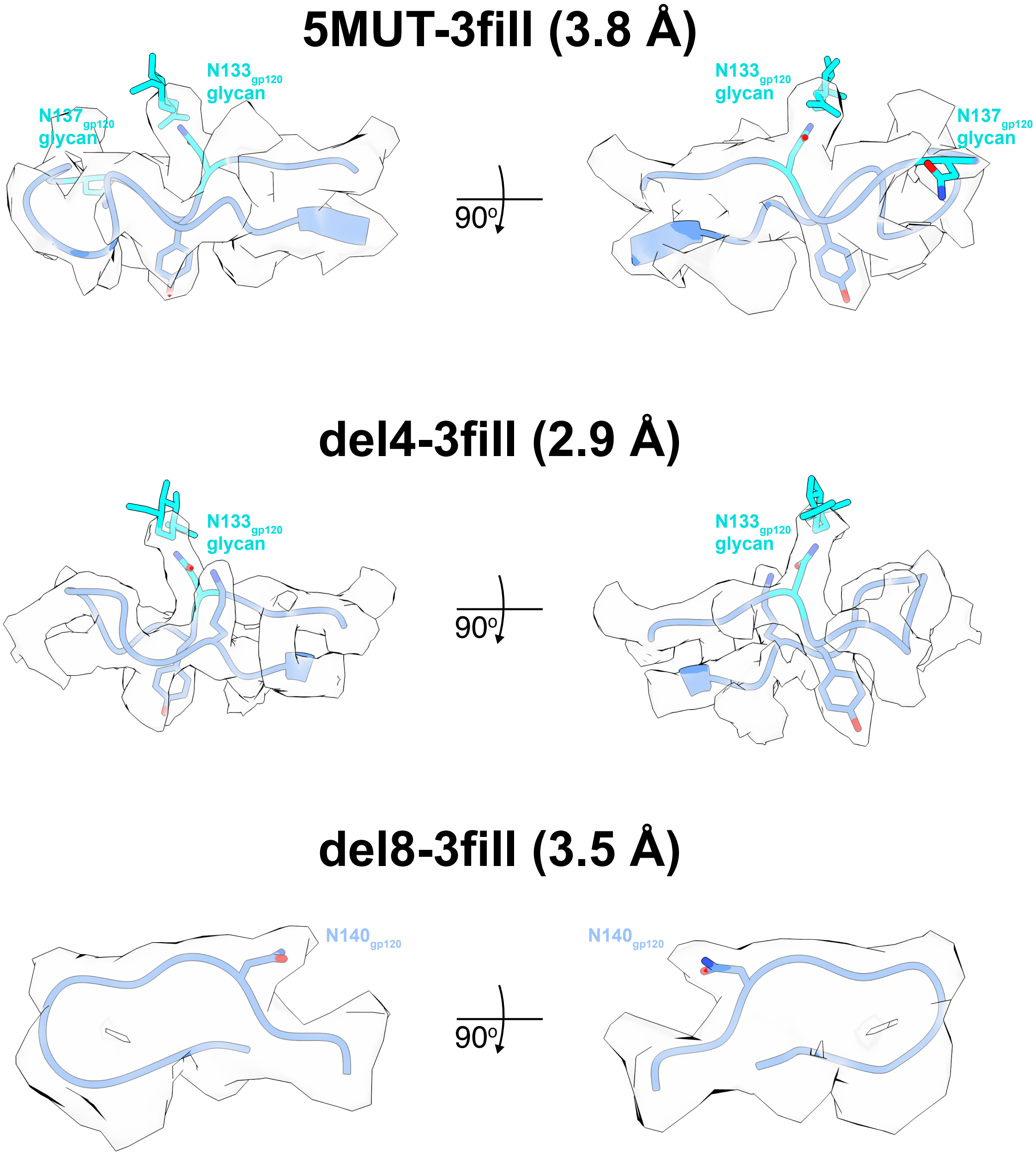

Fig. S14. 5MUT, del4, and del8 trimers exhibit minimal V1 glycosylation. Electron density maps showing minimal to no glycosylation within the V1 loops of 5MUT-3fill (top), del4-3fill (middle), and del8-3fill (bottom) SOSIPs.

A

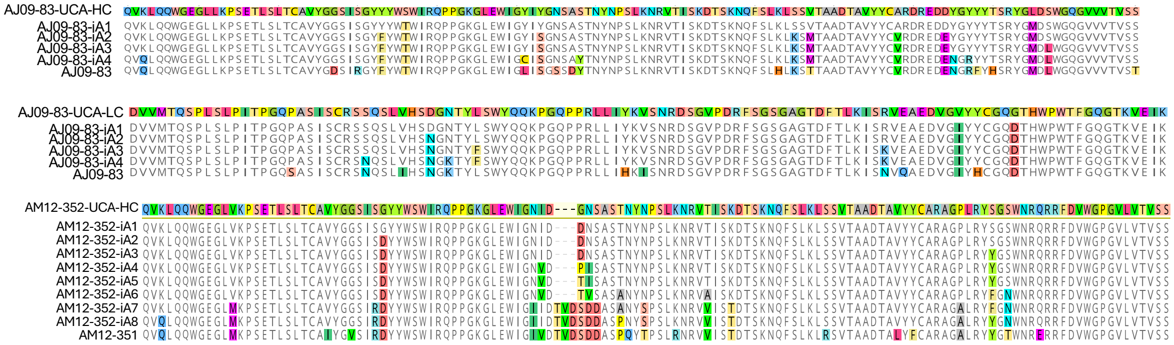

B

mAb ID	BG505. N332	5MUT V1 variants				
		P136S. R143K	5MUT	del3	del4	del8
AM12-352-UCA	1.09	1.07	0.91	0.74	1.00	0.95
AM12-352-iA1	0.88	0.87	0.89	0.87	0.90	0.86
AM12-352-iA2	0.93	0.94	1.01	1.09	0.87	0.93
AM12-352-iA3	1.10	1.06	191.0	14.78	8.58	0.98
AM12-352-iA4	1.06	0.94	155.3	16.06	9.52	0.98
AM12-352-iA5	0.89	0.89	131.0	12.13	7.12	0.84
AM12-352-iA6	1.01	1.00	176.5	18.59	10.43	0.91
AM12-352-iA7	6.32	23.71	622.3	363.3	266.0	2.24
AM12-352-iA8	51.07	120.4	921.3	591.4	356.4	15.63
AJ09-83-UCA	1.70	0.99	0.95	0.95	0.95	0.96
AJ09-83-iA1	0.85	0.88	0.91	0.87	0.92	0.98
AJ09-83-iA2	0.96	0.93	1.04	38.48	22.61	2.69
AJ09-83-iA3	0.98	49.51	232.0	599.4	386.7	260.1
AJ09-83-iA4	47.64	108.4	251.1	549.6	366.9	536.4

MFI Fold Change over Mock

0-3
3-10
10-100
100-1000

Fig. S15. Early intermediate antibodies in the AM12-352 and AJ09-83 lineages bind common SHIV.5MUT escape mutants that have shortened V1 loops. (A) Amino acid alignment of inferred unmutated common ancestor (UCA) and intermediate ancestors (iA1-8) in the AJ09-83 and AM12-352 lineages. Inferred ancestors for the heavy and light chains of the AJ09-83 lineage are shown, while only inferred ancestors for the heavy chain of the AM12-352 lineage are shown. **(B)** Representative heavy and light chain sequences were selected for synthesis and artificially paired. AJ09-83 intermediate heavy chains were paired with intermediate light chains from a similar developmental stage. AM12-352 intermediate heavy chains were paired with the AM12-352-UCA light chain. mRNA-encoded Env trimers were expressed on the surface of 293F cells and binding to UCA and intermediate ancestor mAbs was assessed by flow cytometry. Binding is quantified as fold-change in mean fluorescence intensity (MFI) over mock-transfected cells, and data points with a >3-fold increase are colored as indicated.

A

Site	V635	AM12	V640	V634	V641	V645	AJ09	V633	CM03	AH82	V650	NN58	NN39	V639	V632	V638	CK36	V651	V642	V644	AG99	V631	RM10N011	RM42591	RM6924	RM6923	RM6719	RM6717	RM6442	RM6434	RM6718	RMT283	RM08N021	RM6715	RM6706	RM6708	No of RMs showing selection at site
	Peak TF Loss (%) / Maximum change in average hypervariable loop characteristic																																				
	SHIV.5MUT-infected macaques that developed bNAbs														SHIV.5MUT-infected macaques that failed to								SHIV.BG505.N332-infected macaques (all of which failed to develop bNAbs)														
308	100	100	100	100	100	100	93	100	17	0	100	100	100	100	79	90	19	10	50	6	3	8	90	100	98	100	97	95	98	96	98	3	94	8	0	26	23
359	100	96	100	100	100	100	100	100	100	100	100	100	90	88	89	100	100	4	33	3	0	100	100	100	81	60	41	33	17	64	60	3	2	90	83	62	23
375	40	48	17	100	100	100	7	47	0	3	54	100	100	100	100	0	3	10	0	7	80	11	100	100	100	100	100	100	100	100	100	100	100	100	100	100	22
289	97	100	100	93	100	100	3	3	100	98	3	89	76	73	100	100	82	81	50	0	2	71	100	100	59	4	3	81	30	93	100	3	48	0	0	54	18
241	57	88	10	100	100	100	38	100	71	70	83	100	100	100	96	72	39	93	0	6	7	71	64	100	100	100	3	86	3	39	100	0	17	3	0	69	16
357	100	92	100	96	92	23	40	100	64	24	31	88	79	88	100	100	70	40	67	3	0	100	39	100	100	49	44	67	100	43	96	7	4	65	86	69	16
363	100	100	96	100	100	100	100	44	100	100	100	100	32	91	44	15	100	100	50	73	16	30	93	76	20	2	0	0	0	0	4	3	13	3	2	2	15
347	100	79	60	62	97	9	88	100	97	0	56	95	100	100	100	92	52	100	17	3	0	100	59	98	76	78	66	73	70	100	75	30	13	11	0	2	14
356	97	82	100	92	59	6	40	100	64	24	17	88	47	88	89	97	70	0	33	3	0	100	12	100	76	24	41	33	17	0	60	3	2	45	83	54	12
15	100	77	100	100	92	19	2	25	96	98	93	47	100	47	28	0	8	0	33	100	100	0	10	10	35	0	0	0	0	4	0	30	90	0	0	0	11
440	77	100	96	100	100	68	5	100	3	0	100	100	100	0	8	10	3	4	0	0	0	0	95	46	100	100	0	9	3	4	2	0	6	3	0	3	11
240	0	0	3	100	100	100	18	12	42	35	87	77	100	5	21	4	100	0	0	83	20	50	15	80	29	100	0	0	0	0	0	3	4	0	0	0	9
268	27	100	26	3	97	18	5	0	46	53	3	78	94	33	82	100	58	100	0	0	4	100	61	9	39	11	7	68	43	32	82	3	75	0	0	93	9
325	100	96	69	90	61	87	98	23	73	3	50	100	100	0	4	31	0	7	50	2	0	0	0	12	3	56	3	0	0	0	0	3	0	0	0	0	7
553	4	100	5	0	55	100	61	17	0	58	3	0	100	13	2	84	0	0	50	0	13	0	2	0	0	97	3	81	3	54	100	0	2	0	0	2	7
644	4	3	73	0	100	68	1	76	0	0	91	93	100	100	0	0	90	0	0	2	2	0	3	2	33	89	3	0	0	0	0	3	0	3	0	3	7
87	2	0	0	21	100	3	2	6	32	90	93	100	100	92	3	0	6	0	0	0	2	0	0	34	29	2	0	0	0	0	0	0	0	0	0	15	6
295	78	92	59	93	4	7	8	100	24	98	56	4	0	0	100	8	0	2	0	3	5	0	10	3	73	98	3	5	33	7	20	3	10	70	0	8	6
297	78	92	59	93	4	7	7	100	24	98	56	4	0	0	100	8	0	0	0	2	5	0	10	3	73	98	3	0	30	7	20	3	10	68	0	8	6
339	8	55	92	89	13	70	15	85	7	0	16	15	100	31	77	0	22	20	50	39	8	33	3	36	19	53	8	86	0	0	8	7	8	5	0	95	6
10	5	84	0	100	55	100	73	25	0	0	52	20	71	68	27	0	87	0	50	0	0	4	83	3	14	22	3	0	0	0	0	0	0	3	3	0	5
232	26	100	5	0	92	0	12	0	3	60	3	68	100	81	52	30	10	100	0	0	0	0	19	7	49	0	0	0	10	0	60	0	0	0	0	8	5
291	15	18	100	93	84	100	3	3	0	0	3	3	0	3	0	2	9	4	50	0	0	0	0	80	0	4	0	0	0	0	0	3	0	0	0	8	5
332	37	17	21	95	17	100	100	100	93	18	9	5	21	0	2	0	6	3	50	0	11	0	2	0	0	0	10	0	0	4	0	3	0	0	0	0	5
343	8	8	2	89	36	3	65	92	0	6	98	59	100	94	21	0	6	2	2	6	0	12	0	0	0	11	3	8	0	0	0	3	2	0	0	38	5
411	96	100	52	100	74	23	37	24	0	98	14	19	13	0	44	0	0	2	50	44	5	0	5	46	11	98	0	0	0	61	20	0	15	13	0	3	5
413	92	100	52	100	77	23	35	24	0	6	14	19	72	0	22	0	0	2	50	0	0	0	88	46	8	98	0	0	0	11	16	0	6	0	0	3	5
557	9	29	0	0	84	4	45	0	0	95	0	0	100	5	0	0	2	0	0	3	7	10	0	2	0	96	93	33	0	0	3	0	2	0	0	0	5
322	0	100	12	3	3	3	0	6	4	18	3	0	13	67	100	80	0	93	0	0	0	0	0	0	3	4	3	0	0	0	0	3	0	0	0	0	4
330	73	73	54	100	100	90	8	24	84	58	0	5	0	0	2	30	0	3	0	0	2	0	2	0	0	0	3	4	0	4	0	3	0	0	0	0	4
507	100	6	12	7	0	3	50	5	6	0	11	6	48	100	7	0	14	3	2	6	3	4	43	35	37	34	3	5	83	3	10	23	6	100	0	59	4
535	38	41	96	4	8	5	4	7	36	3	6	26	96	3	70	2	0	5	8	24	0	0	2	54	53	93	93	0	0	3	0	0	33	0	0	0	4
715	8	3	8	29	55	100	39	100	4	6	4	84	4	3	35	0	3	2	33	49	0	81	0	0	0	0	0	0	0	0	0	0	0	0	0	0	4
20	100	52	3	100	7	3	2	0	3	58	3	32	50	0	3	6	4	93	0	3	6	0	3	6	3	3	0	0	0	3	0	0	2	3	0	0	3
24	12	25	32	17	21	74	3	50	0	0	5	48	0	88	2	4	6	100	23	7	3	17	8	51	73	0	10	90	75	0	5	3	0	3	0	0	3
326	17	3	10	7	100	0	7	17	7	0	96	4	100	36	2	12	12	23	0	6	16	0	0	0	3	0	3	0	0	3	0	3	0	0	0	0	3
334	13	15	21	69	0	81	95	80	64	10	6	3	21	0	2	0	6	0	50	0	9	0	0	0	0	0	10	0	0	4	0	3	0	0	0	0	3
341	8	2	92	89	11	70	3	85	5	0	10	9	59	31	5	0	17	18	50	3	5	0	0	6	3	8	3	71	0	0	0	3	0	3	0	11	3
346	83	3	29	0	97	5	42	0	7	0	0	42	100	6	78	3	13	0	0	15	0	0	0	0	4	9	72	0	15	7	3	3	0	0	0	15	3
354	65	61	40	5	95	41	7	37	86	9	3	66	95	0	0	3	10	6	8	7	0	0	5	0	3	0	72	2	0	4	5	7	2	3	2	11	3
607	0	0	0	0	60	100	0	0	4	0	89	0	0	100	0	10	52	0	50	0	0	0	0	0	0	0	0	0	0	0	0	0	0	0	0	0	3
9	2	3	0	0	20	74	18	0	0	0	73	10	7	88	3	12	39	0	50	3	0	0	3	0	8	0	0	0	0	0	2	0	86	0	3	15	2
31	5	45	100	0	68	2	0	3	21	0	18	67	10	13	67	4	55	100	33	50	0	30	3	29	3	2	10	0	0	0	0	0	0	3	0	0	2
63	8	3	66	17	0	6	0	100	0	0	0	3	0	0	0	0	10	2	0	2	6	0	2	0	0	100	20	0	73	0	0	0	0	3	3	0	2
117	0	41	3	0	0	0	25	12	7	3	0	58	100	55	33	4	0	5	2	3	0	50	0	80	3	2	0	0	3	3	0	0	0	3	0	0	2
181	0	53	0	3	2	0	0	8	0	0	13	12	0	9	8	100	39	100	0	3	0	0	5	0	0	3	0	0	0	0	4	0	0	0	0	2	2
340	8	61	12	48	58	16	5	0	36	3	23	67	84	31	21	20	6	74	2	11	2	83	45	6	0	9	3	14	8	0	2	7	19	0	0	8	2
386	0	0	6	83	100	0	0	3	0	0	15	3	0	13	14	0	3	0	0	0	0	0	5	2	0	0	33	0	0	0	0	0	0	0	0	0	2
417	0	64	0	92	100	74	0	0	3	0	2	0	42	0	0	0	0	0	0	0	0	0	0	0	0	0	0	0	0	0	0	0	0	0	0	0	2
430	74	100	88	46	74	38	2	10	3	0	63	11	75	0	2	0	29	3	0	0	0	20	0	66	10	44	0	0	4	4	13	0	2	0	0	0	2
467	0	91	8	69	95	42	28	25	17	0	54	47	4	31	7	0	6	0	0	0	3	0	5	0	27	58	0	0	0	3	0	0	0	11	0	0	2
5	8	12	2	5	0	0	18	0	6	85	3	6	25	5	0	4	4	0	8	6	0	0	5	0	0	0	0	0	0	0	0	0	0	3	0	31	1
6	0	47	2	0	23	4	6	2	3	3	5	3	0	0	0	0	0	0	0	3	0	4	0	0	0	0	0	0	0	0	0	3	10	3	83	0	1
7	8	3	0	25	0	3	0	100	0	3	56	13	0	0	0	0	35	0	0	0	0	17	0	2	76	2	0	0	0	0	0	0	2	3	0	0	1
16	0	6	13	25	5	3	2	5	3	57	3	0	0	8	0	0	55	0	33	26	0	0	0	0	2	0	0	0	0	0	36	17	0	85	0	0	1
21	4	61	3	0	0	4	0	0	100	0	0	16	4	6	0	0	0	6	0	34	0	0	3	3	0	0	0	0	0	0	2	17	0	0	0	0	1
28	85	0	5	0	0	0	0	0	6	0	3	3	5	0	0	0	4	33	0	6	0	33	2	0	2	0	7	0	0	0	0	0	2	0	0	0	1
30	58	46	3	62	3	2	0	5	3	28	7	4	11	0	2	0	7	6	23	0	0	8	0	3	3	4	0	2	3	0	0	0	13	98	0	8	1
60	0	3	0	3	0	0	3	0	3	0	0	3	5	3	4	0	3	100	0	0	0	0	2	0	0	2	0	0	0	0	0	0	0	0	0	0	1
109	0	0	0	0	3	0	0	3	0	0	24	0	3	9	0	0	3	0	0	3	0	0	0	0	0	0	0	0	0	4	0	0	0	0	0	83	1
155	7	0	4	3	97	0	0	17	0	3	0	3	0	0	0	0	0	8	0	0	0	0	0	0	5	0	0	0	0	0	0	0	0	0	0	0	1
178	0	0	0	0	22	7	6	0	2	3	7	3	17	100	0	0	39	0	17	0	20	7	0	3	0	0	0	0	0	0	0	0	2	5	0	0	1
200	0	3	3	27	0	0	60	2	8	93	0	3	4	8	2	2	0	0	0	3	0	0	0	2	0	0	0	2	0	0	0	0	0	0	0	0	1
269	15	73	5	3	2	0	0	0	13	0	2	10	4	9	100	48	0	78	0	0	0	21	19	0	0	0	3	0	5	11	4	0	0	0	0	0	1
279	12	3	2	3	3	3	0	48	3	0	6	7	3	0	3	0	0	0	50	3	0	0	68	100	0	0	0	0	0	0	0	0	0	0	0	0	1
290	0	0	3	0	3	3	1	3	14	0	2	21	0	100	0	18	6	0	8	3	0	17	0	0	0	78	0	0	0	4	3	3	0	0	0	3	1
316	0	3	3	3	4	0	2	0	43	0	0	0	0	0	38	0	2	0	0	0	89	0	0	0	3	2	3	0	0	0	0	0	0	0	0	0	1
335	7	2	2	15	54	0	0	0	3	0	4	3	3	0	15	0	6	0	0	0	0	0	3	98	0	2	3	0	4	7	0	3	0	3	0	3	1
351	35	5	3	5	4	3	3	0	6	0	7	29	25	32	4	18	9	67	0	72	0	21	80	29	2	24	3	0	3	0	4	3	0	0	0	0	1
392	2	0	0	64	0	3	0	5	0	0	0	4	42	6	100	0	0	0	0	2	0	0	3	2	0	2	0	38	0	0	13	0	0	0	0	0	1
415	4	2	0	20	5	85	12	0	3	3	2	0	5	0	3	0	0	7	0	0	2	0	66	0	0	3	10	0	0	4	0	0	2	0	0	31	1
496	0	0	0	0	84	0	11	0	0	39	0	3	0	9	3	10	0	0	8	0	4	0	0	0	0	36	3	13	0	0	0	0	2	0	0	0	1
588	12	3	0	7	0	3	5	10	0	93	0	0	63	0	0	4	0	3	0	14	7	0	2	0	3	0	0	0	4	0	0	20	4	3	4	2	1
616	5	0	4	4	4	0	0	5	0	3	0	3	0	5	0	12	5	0	33	83	0	0	2	0	0	3	0	0	0	0	0	0	2	0	0	0	1
618	0	3	3	4	3	0	0	0	0	0	3	0	0	0	22	0	3	3	0	83	0	0	2	0	0	0	0	0	0	3	0	3	2	0	0	0	1
682	0	0	4	0	0	0	1	0	0	0	0	3	0	0	0	0	0	0	0	0	0	0	2	0	0	0	0	0	3	0	0	0	0	0	0	100	1
700	5	0	2	2	3	0	2	0	3	0	0	3	5	0	4	6	4	0	4	0	0	7	2	6	0	0	0	0	0	0	0	0	2	100	0	0	1
705	17	63	3	6	76	3	4	25	0	0	39	9	0	100	6	6	4	5	8	3	0	17	0	3	3	13	0	59	0	3	3	0	2	3	0	3	1
No. of sites selected in RM	15	20	14	26	26	17	7	17	9	12	12	14	23	19	13	10	6	12	0	4	3	7	9	13	6	14	4	7	4	4	7	1	4	6	5	5	

B

C

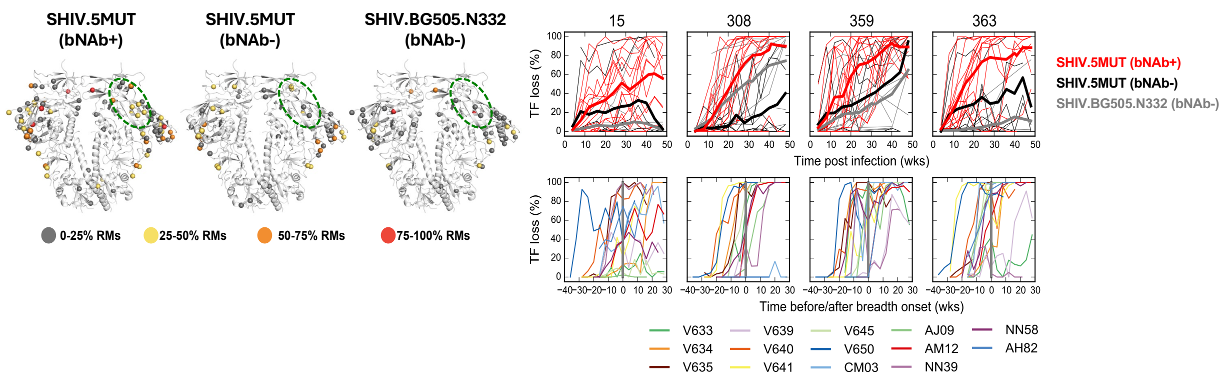

Fig. S16. Env sequence evolution in SHIV-infected rhesus macaques. (A) Analysis of the evolving Env quasispecies in all SHIV-infected macaques for sites under immune selection. Three groups, including 14 SHIV.5MUT-infected animals that developed bNAbs, 8 SHIV.5MUT-infected animals that did not develop bNAbs, and 14 SHIV.BG505.N332-infected animals that did not develop bNAbs, were subjected to LASSIE analysis, which identifies residues where mutations have altered the encoded amino acid in at least 80% of sequences at one or more timepoints. All

LASSIE-selected sites are highlighted in blue, with the number indicating the maximum percentage of sequences from any timepoint that exhibit loss of the transmitted founder (TF) residue for each macaque. **(B)** Structural mapping of LASSIE-selected sites in each group onto the BG505 Env (PDB ID: 9EHL). Sites are color-coded as indicated based on recurrence in multiple macaques. The green ellipse demarcates the approximate location of the V3-glycan bNAb epitope. **(C)** Same analysis as shown in **Fig. 6C**, but for sites that were not temporally associated with the acquisition of plasma breadth. Top: Longitudinal mutation frequency (%TF loss) at four sites enriched in SHIV.5MUT-infected macaques that developed bNAbs. Red and black curves denote SHIV.5MUT-infected macaques that did versus did not develop bNAbs, respectively, while grey curves indicate SHIV.BG505.N332-infected animals (all of which failed to develop bNAbs). Thin lines indicate data from individual animals and thick lines represent group averages. Bottom: Mutation frequency (%TF loss) in macaques that developed bNAbs, with the timepoint at which plasma breadth is first detected (defined as at least 1 heterologous virus with reciprocal $ID_{50} > 20$) set to zero weeks (wks). Different colors indicate different macaques. TF, transmitted founder.

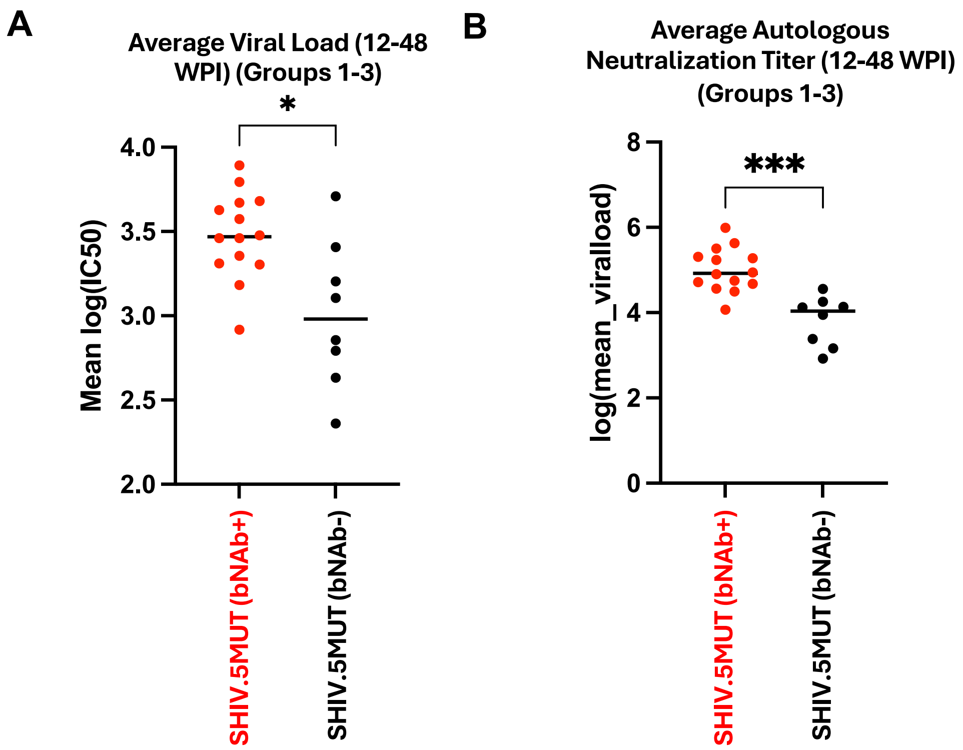

Fig. S17. bNAb development in SHIV.5MUT-infected macaques is associated with higher viral loads and autologous neutralization titers. (A) Average viral loads in SHIV.5MUT-infected macaques (Groups 1-3) between weeks 12-48 post-infection, quantified as log(mean viral load). **(B)** Average autologous neutralization titers in SHIV.5MUT-infected macaques (Groups 1-3), represented as mean(log(IC_{50})). Mann-Whitney U test was used. Not significant (ns) $P > 0.05$; $^*P < 0.05$; $^{**}P < 0.01$; $^{***}P < 0.001$.

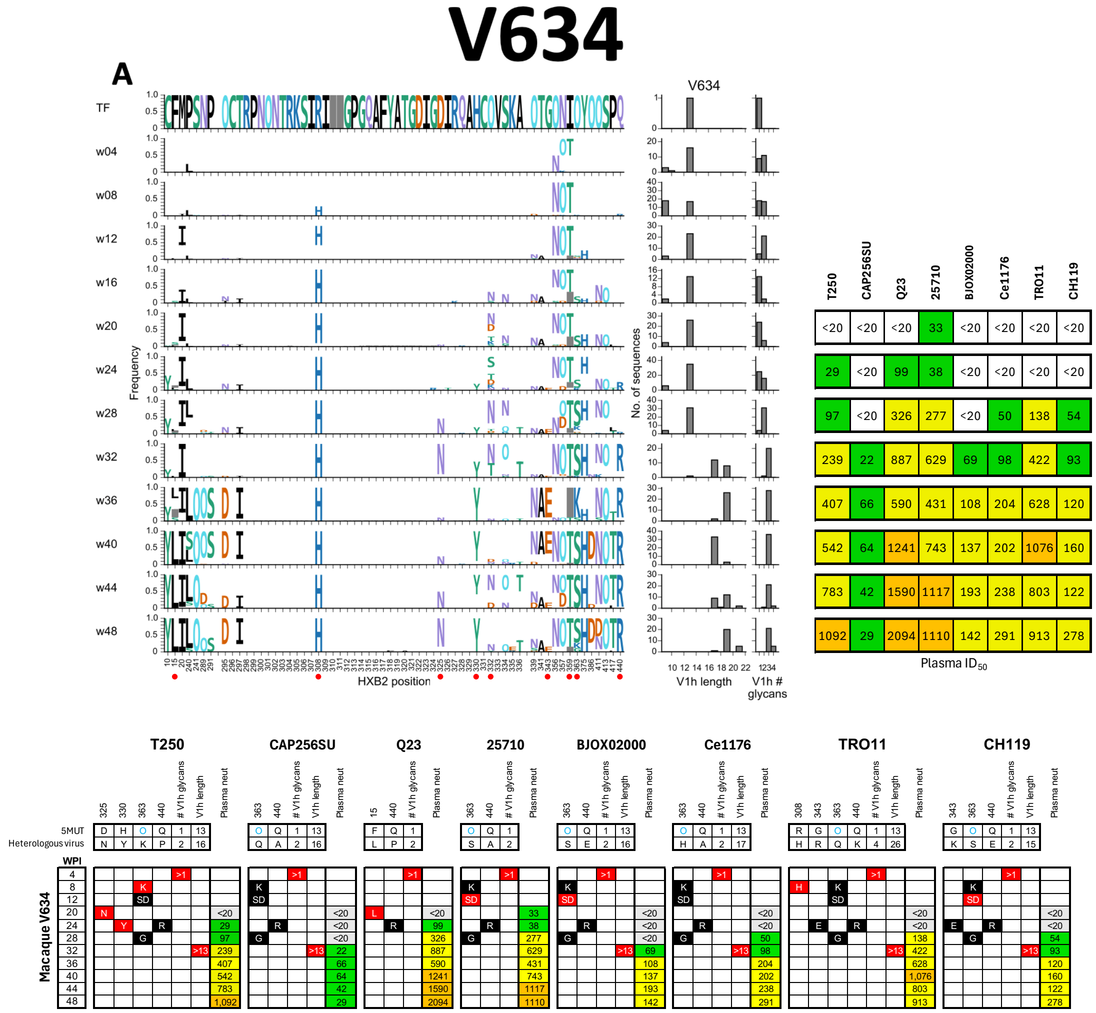

Fig. S18. Env evolution and development of plasma breadth in rhesus macaque V634. See **Fig. 6A** and **Fig. 7B** for details regarding figure layout and **Methods** for a description of the analysis. **(A)** Longitudinal patterns of mutations in Env and development of neutralization breadth. **(B)** Integrated information from the three graphs in **(A)**. 20/21 amino acid changes in key sites, 4/6 V1h elongations, and 8/8 V1h additions relevant to a given pseudovirus were detected prior to neutralization breadth being observed in the plasma.

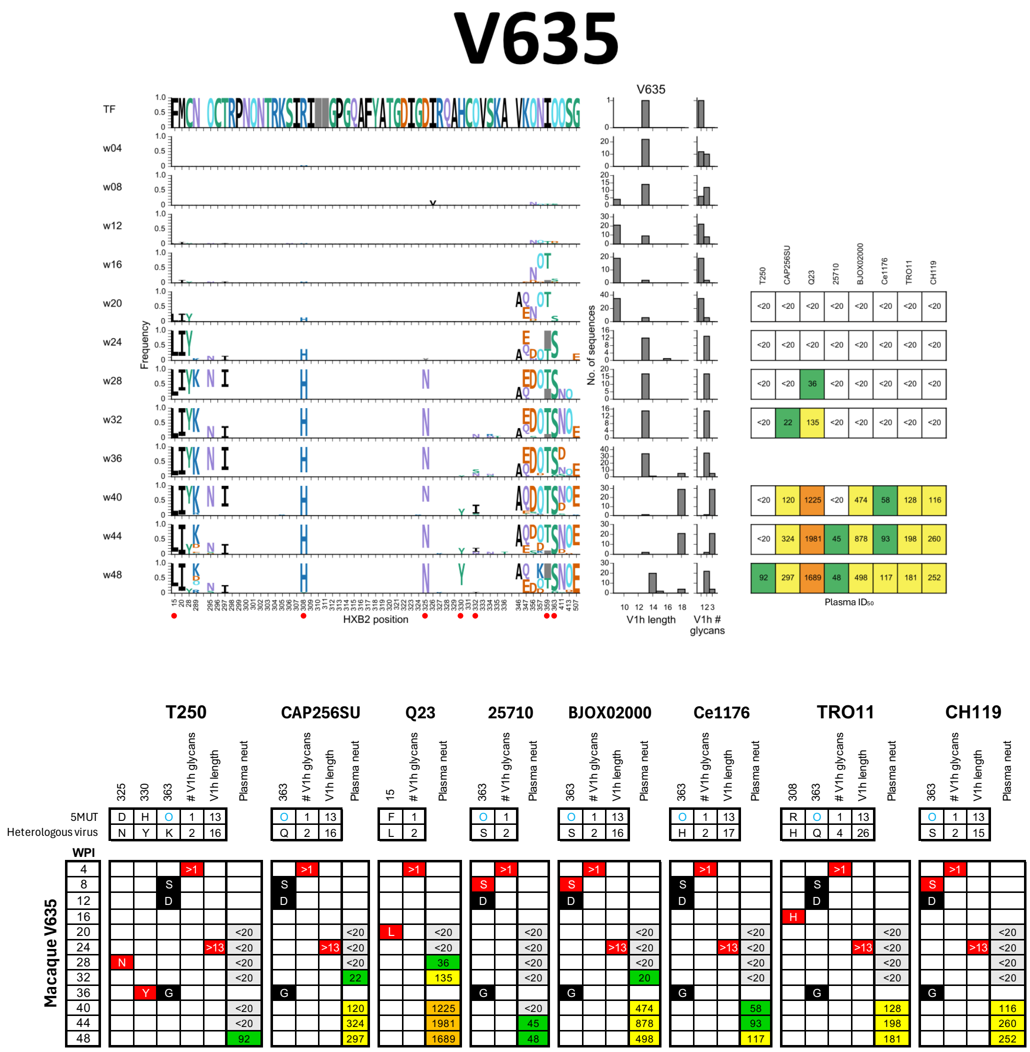

Fig. S19. Env evolution and development of plasma breadth in rhesus macaque V635. See **Fig. 6A** and **Fig. 7B** for details regarding figure layout and **Methods** for a description of the analysis. **(A)** Longitudinal patterns of mutations in Env and development of neutralization breadth. **(B)** Integrated information from the three graphs in **(A)**. 11/11 amino acid changes in key sites, 6/6 V1h elongations, and 8/8 V1h glycans additions relevant to a given pseudovirus were detected prior to neutralization breadth being observed in the plasma.

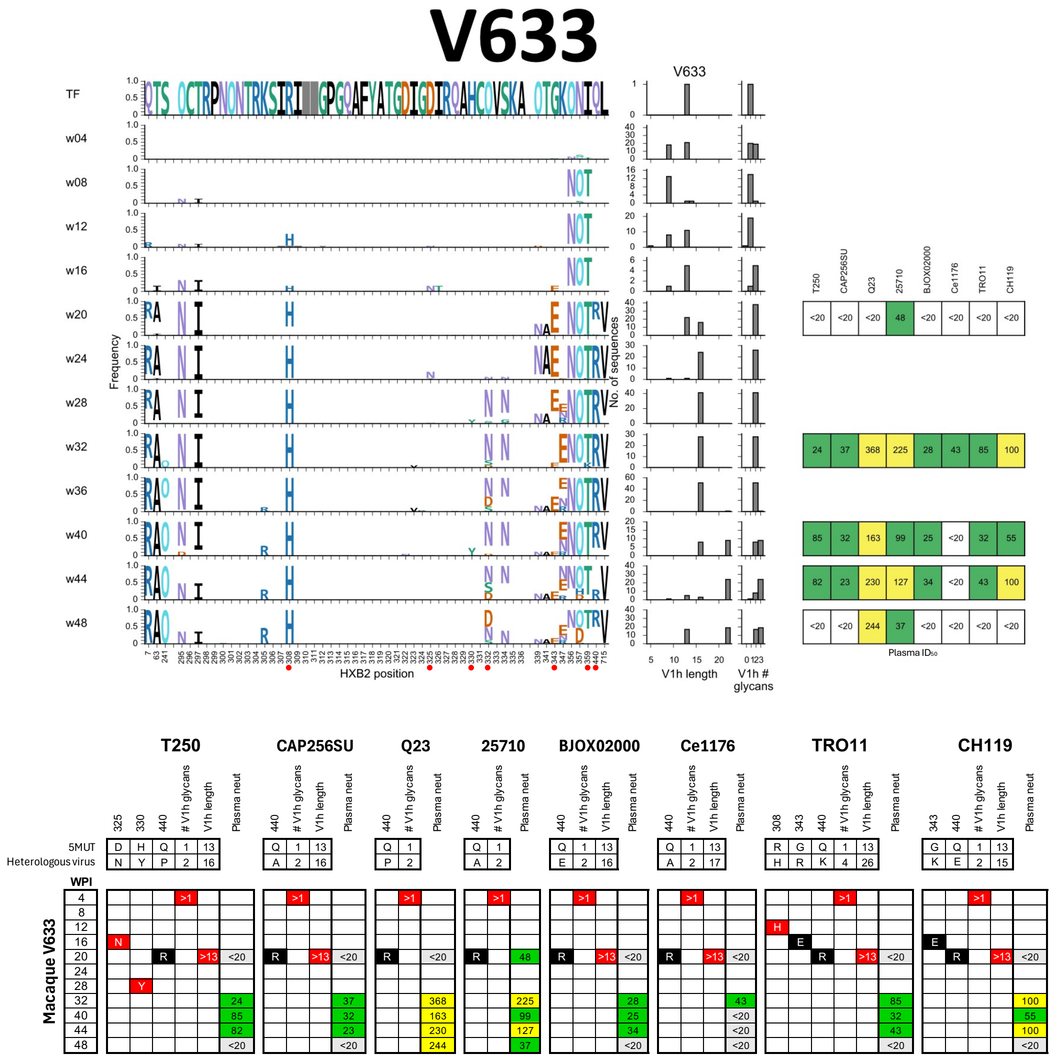

Fig. S20. Env evolution and development of plasma breadth in rhesus macaque V633. See **Fig. 6A** and **Fig. 7B** for details regarding figure layout and **Methods** for a description of the analysis. **(A)** Longitudinal patterns of mutations in Env and development of neutralization breadth. **(B)** Integrated information from the three graphs in **(A)**. 13/13 amino acid changes in key sites, 6/6 V1h elongations, and 8/8 V1h glycan additions relevant to a given pseudovirus were detected prior to or concurrent with neutralization breadth being observed in the plasma. The D325N and H330Y variants were rare but present.

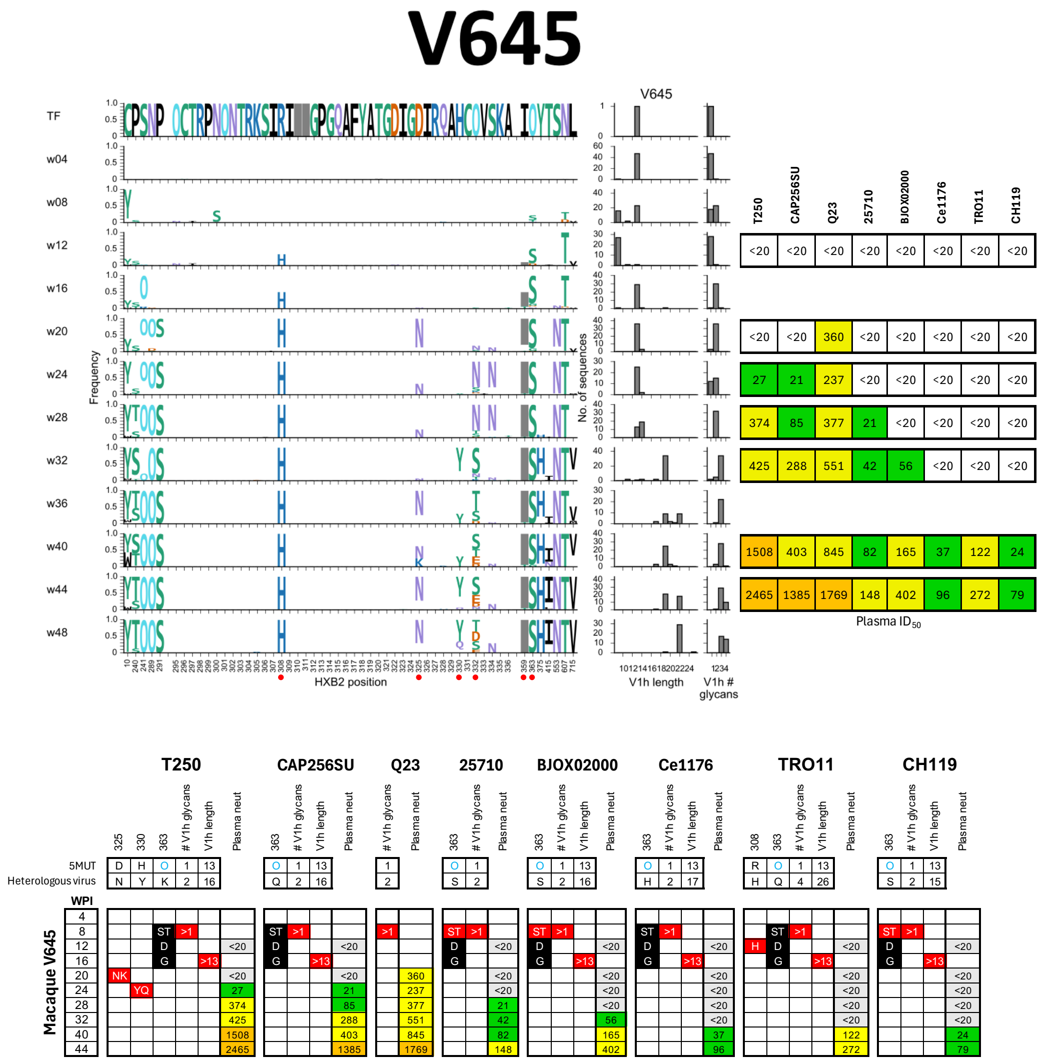

Fig. S21. Env evolution and development of plasma breadth in rhesus macaque V645. See **Fig. 6A** and **Fig. 7B** for details regarding figure layout and **Methods** for a description of the analysis. **(A)** Longitudinal patterns of mutations in Env and development of neutralization breadth. **(B)** Integrated information from the three graphs in **(A)**. 10/10 amino acid changes in key sites, 6/6 V1h elongations, and 8/8 V1h glycan additions relevant to a given pseudovirus were detected prior to or concurrent with neutralization breadth being observed in the plasma. O363S was rare at week 8 but common by week 16. Increases in V1h length were initially rare at week 16 but became common by week 32, perhaps contributing to bNAb selection for breadth and potency. Plasma neutralization of Q23 was detected first, perhaps in part because it has a short V1h region and a glycan at O363, much like 5MUT.

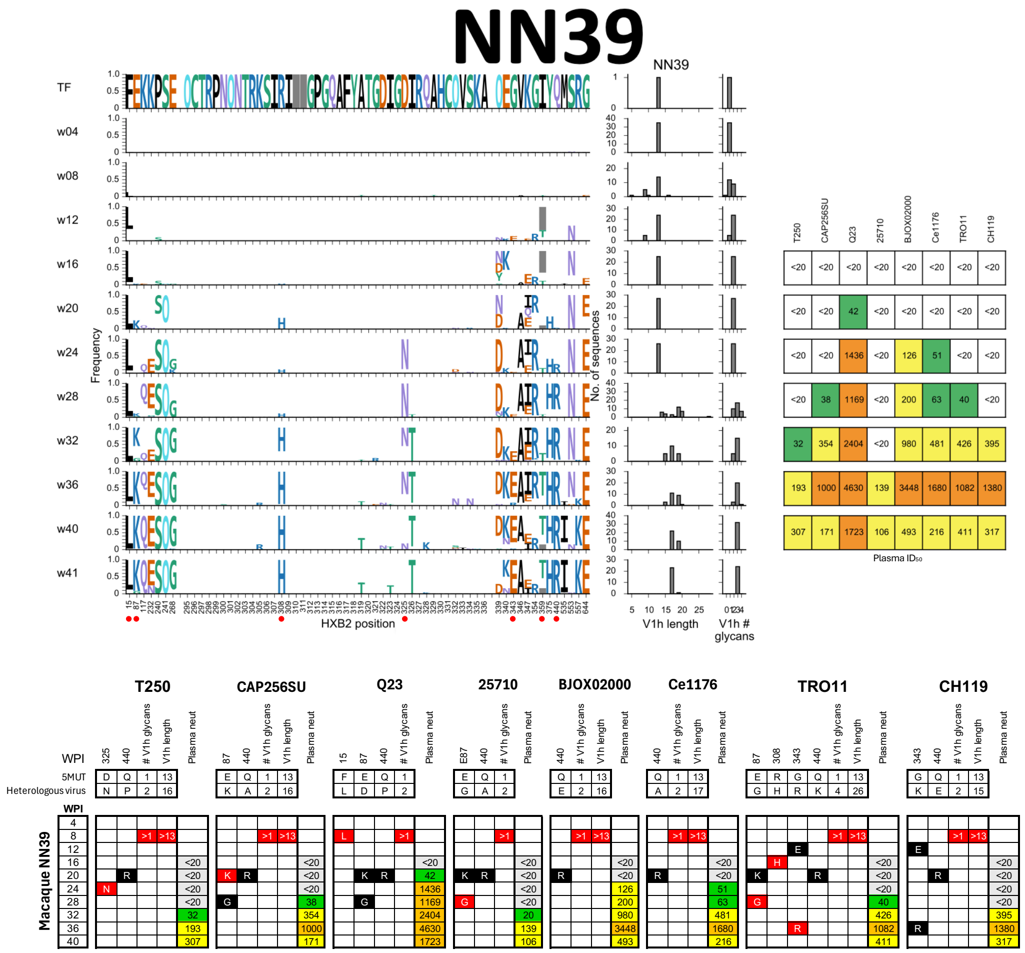

Fig. S22. Env evolution and development of plasma breadth in rhesus macaque NN39. See **Fig. 6A** and **Fig. 7B** for details regarding figure layout and **Methods** for a description of the analysis. **(A)** Longitudinal patterns of mutations in Env and development of neutralization breadth. **(B)** Integrated information from the three graphs in **(A)**. 17/17 amino acid changes, 6/6 V1h elongations, and 8/8 V1h glycan additions relevant to a given pseudovirus were detected prior to or concurrent with neutralization breadth being observed in the plasma. To recognize 25710 and TRO11, emergence of the E87G variant may be required, rather than simply the loss of the 5MUT-encoded E87 residue. Similarly, V1 elongation started at week 8 but was rare until week 28, which coincides with the timepoint at which neutralization potency increased substantially against heterologous viruses with longer V1h loops. Plasma neutralization of Q23 was detected earlier at week 20, perhaps in part because it has a short V1h region much like 5MUT.

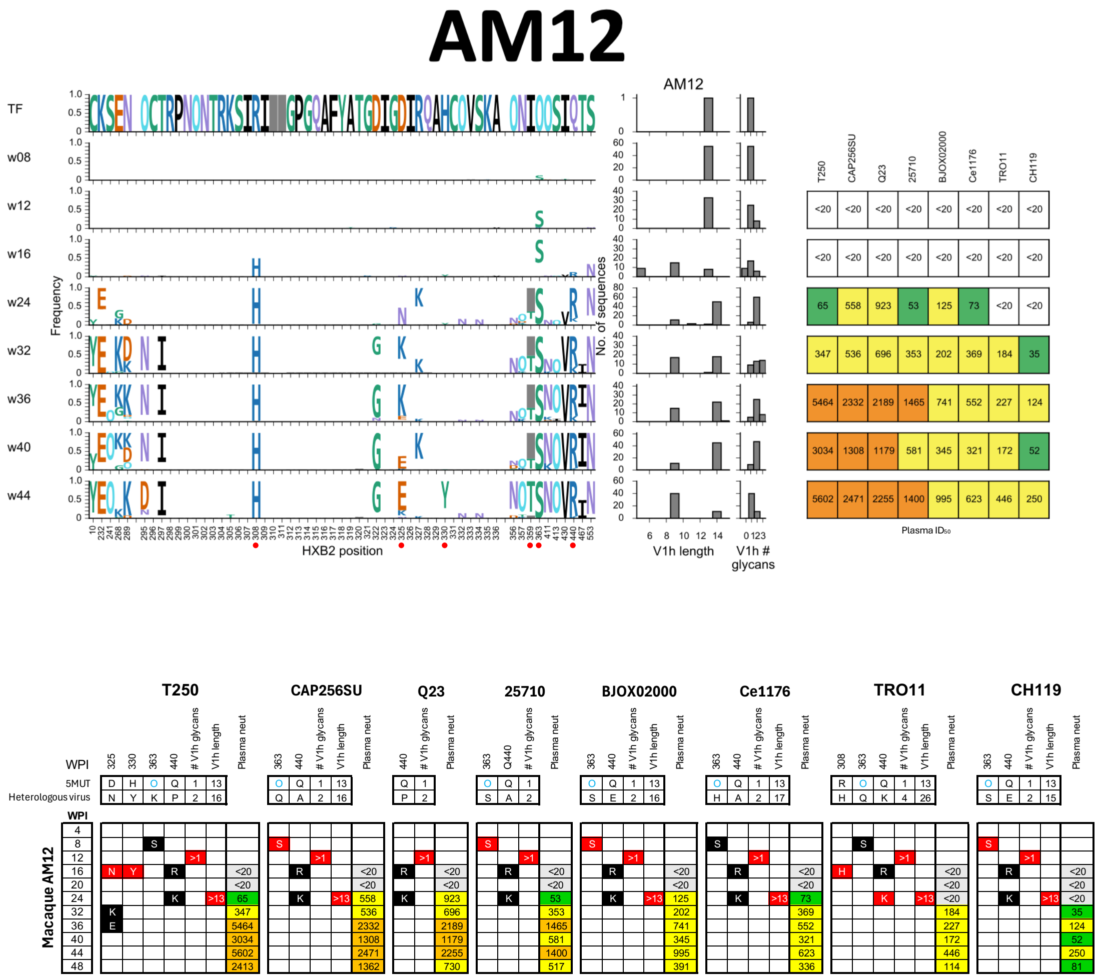

Fig. S23. Env evolution and development of plasma breadth in rhesus macaque AM12. See **Fig. 6A** and **Fig. 7B** for details regarding figure layout and **Methods** for a description of the analysis. **(A)** Longitudinal patterns of mutations in Env and development of neutralization breadth. **(B)** Integrated information from the three graphs in **(A)**. 18/18 amino acid changes, 6/6 V1h elongations, and 8/8 V1h glycan additions relevant to a given pseudovirus were detected prior to or concurrent with neutralization breadth being observed in the plasma.

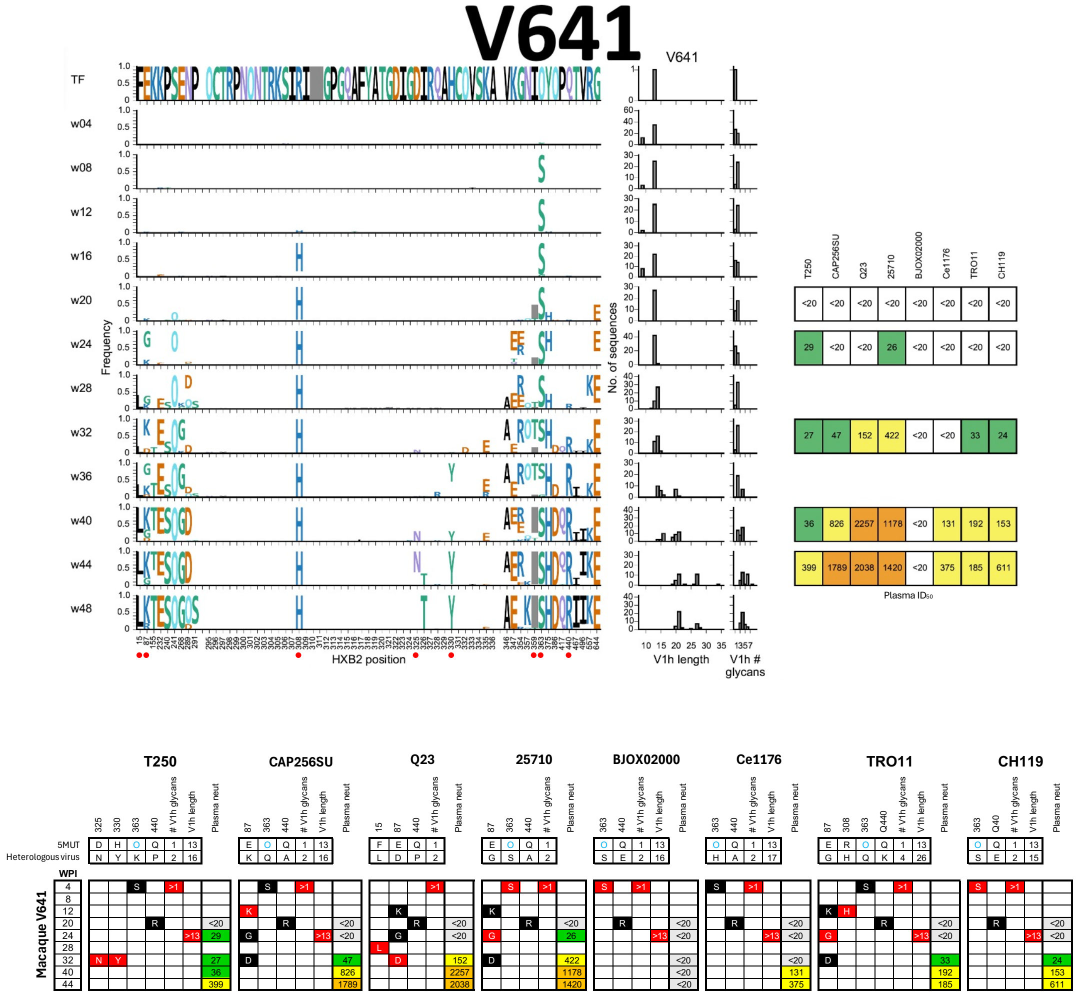

Fig. S24. Env evolution and development of plasma breadth in rhesus macaque V641. See **Fig. 6A** and **Fig. 7B** for details regarding figure layout and **Methods** for a description of the analysis. **(A)** Longitudinal patterns of mutations in Env and development of neutralization breadth. **(B)** Integrated information from the three graphs in **(A)**. 19/21 amino acid changes, 5/5 V1h elongations, and 7/7 V1h glycan additions relevant to a given pseudovirus were detected prior to or concurrent with neutralization breadth being observed in the plasma. The two exceptions were D325N and H330Y, which were first detected at week 32 despite T250 being weakly neutralized by the plasma at week 24. This potency increased by over 10-fold following subsequent emergence of the D325N and H330Y variants. For this animal, the specific residue at position 87 may be important for recognizing certain viruses, rather than simply the loss of the 5MUT-encoded E87 residue. Indeed, E87K/D/G variants were sampled in the quasispecies.

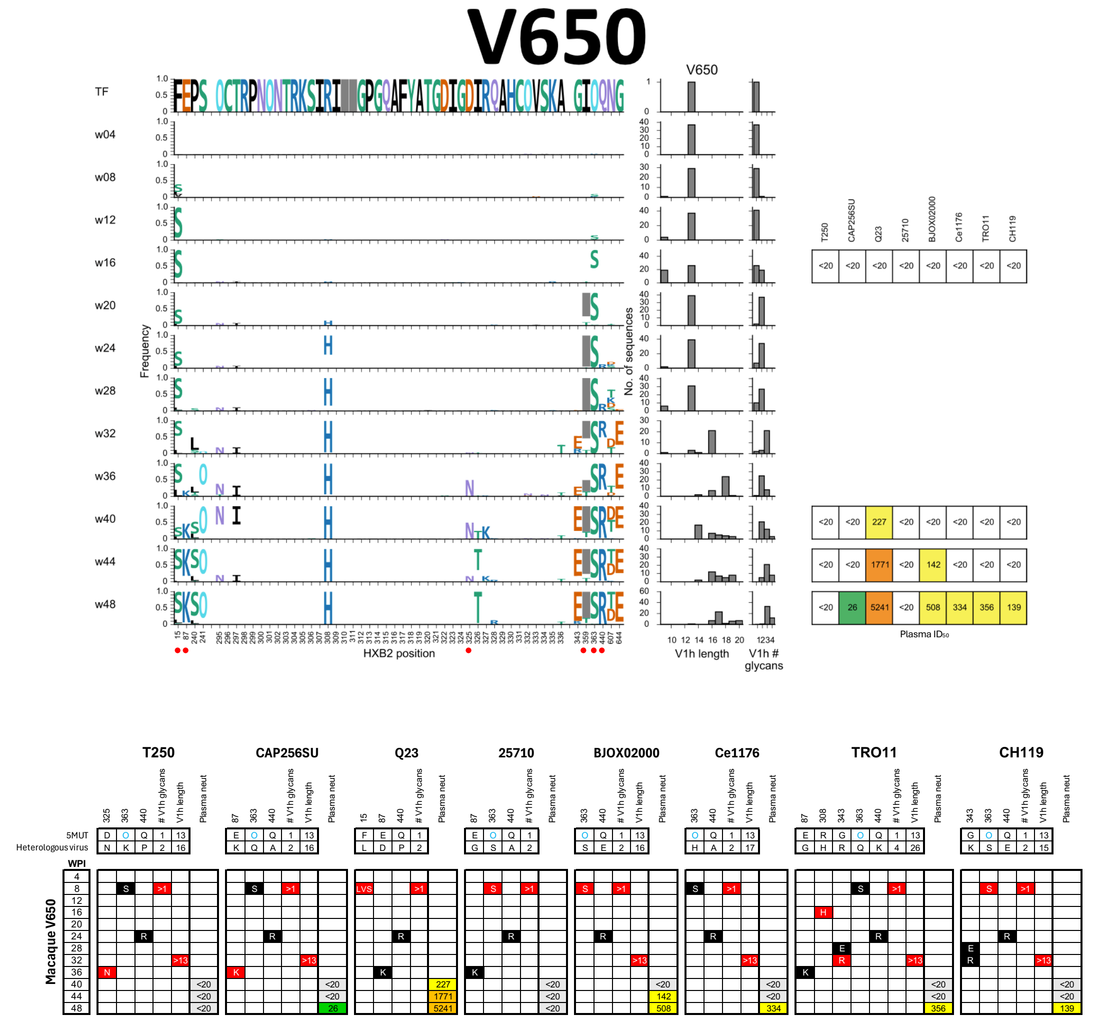

Fig. S25. Env evolution and development of plasma breadth in rhesus macaque V650. See **Fig. 6A** and **Fig. 7B** for details regarding figure layout and **Methods** for a description of the analysis. **(A)** Longitudinal patterns of mutations in Env and development of neutralization breadth. **(B)** Integrated information from the three graphs in **(A)**. 18/18 amino acid changes, 5/5 V1h elongations, and 6/6 V1h glycan additions relevant to a given pseudovirus were detected prior to or concurrent with neutralization breadth being observed in the plasma. O363S is very rare at week 8 but becomes common by week 20. V1h-elongated variants are common by week 32, prior to recognition of heterologous viruses with longer V1h lengths.

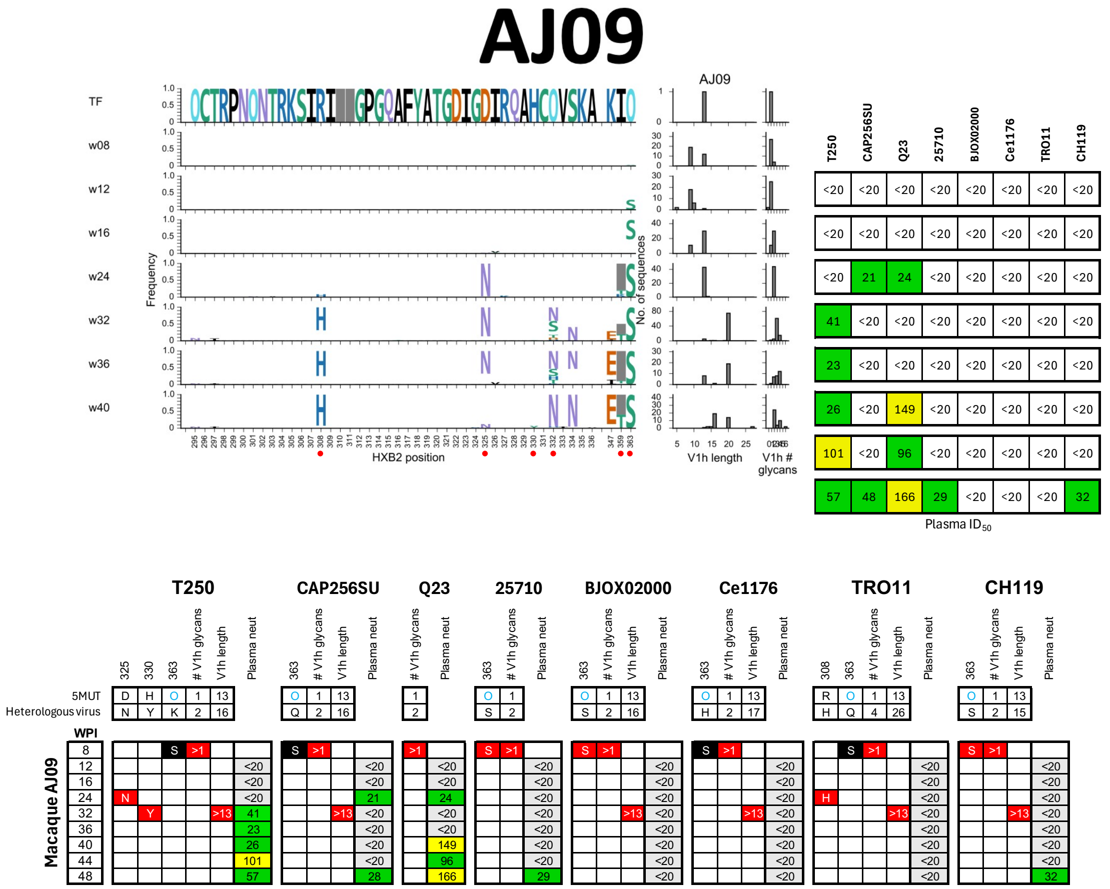

Fig. S26. Env evolution and development of plasma breadth in rhesus macaque AJ09. See **Fig. 6A** and **Fig. 7B** for details regarding figure layout and **Methods** for a description of the analysis. **(A)** Longitudinal patterns of mutations in Env and development of neutralization breadth. **(B)** Integrated information from the three graphs in **(A)**. 6/6 amino acid changes, 2/3 V1h elongations, and 5/5 V1h glycan additions relevant to a given pseudovirus were detected prior to or concurrent with neutralization breadth being observed in the plasma. CAP256SU, which has a long V1h region, was weakly neutralized prior to detection of V1h-elongated variants in the quasispecies. The rapid near-complete loss of the critical N332 glycan (O332) at week 32 may drive near-complete escape of the maturing bNAb lineage and contribute to the limited breadth and potency observed in the plasma.

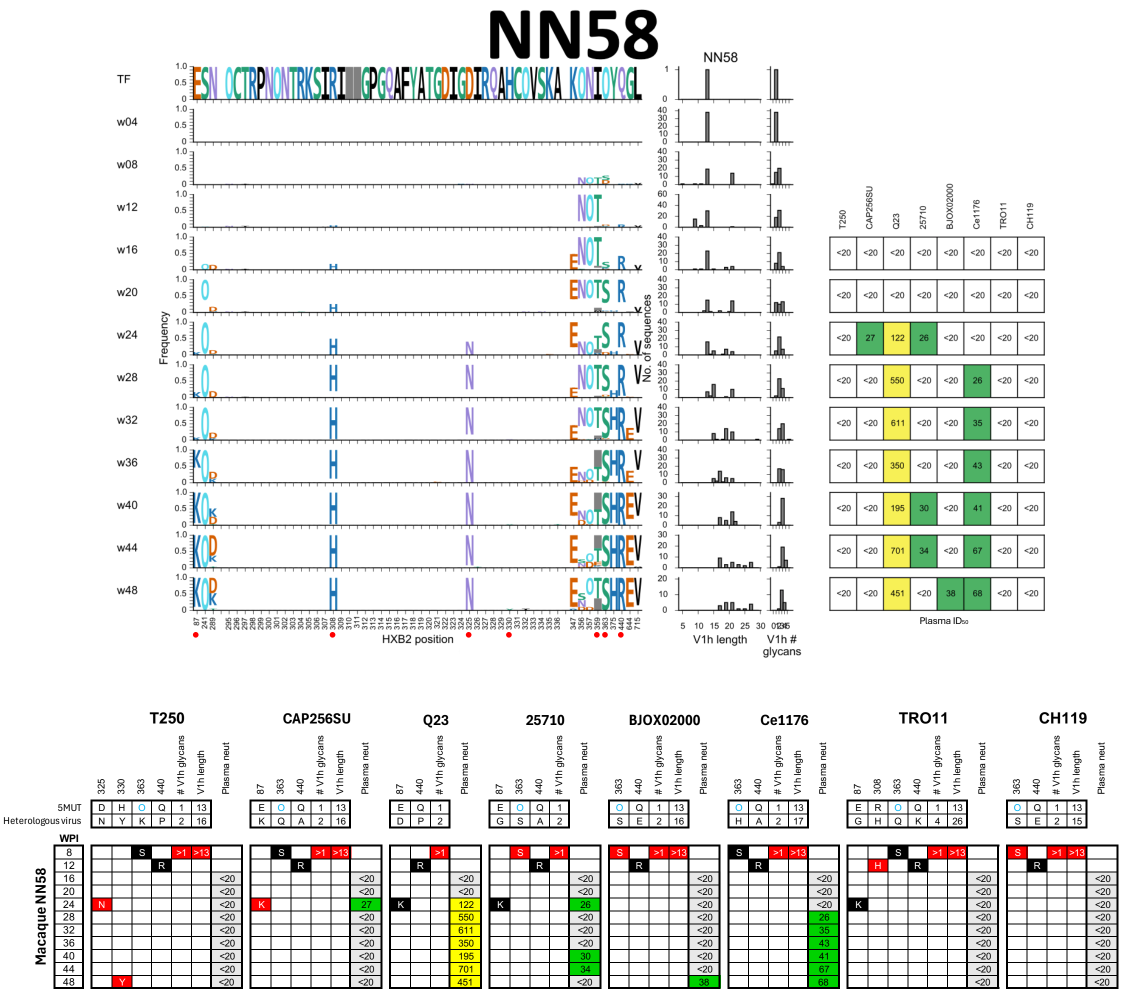

Fig. S27. Env evolution and development of plasma breadth in rhesus macaque NN58. See **Fig. 6A** and **Fig. 7B** for details regarding figure layout and **Methods** for a description of the analysis. **(A)** Longitudinal patterns of mutations in Env and development of neutralization breadth. **(B)** Integrated information from the three graphs in **(A)**. 12/12 amino acid changes, 3/3 V1h elongations, and 5/5 V1h glycan additions relevant to a given pseudovirus were detected prior to or concurrent with neutralization breadth being observed in the plasma.

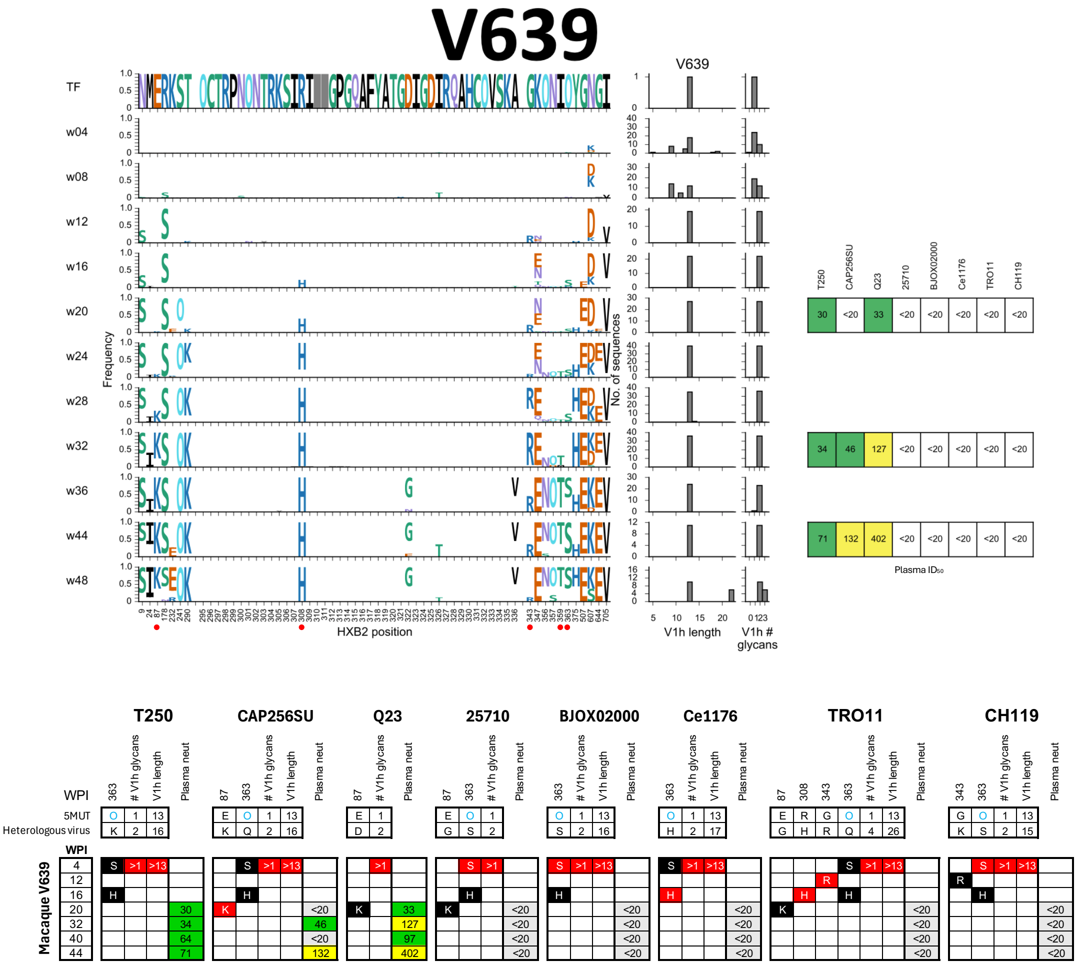

Fig. S28. Env evolution and development of plasma breadth in rhesus macaque V639. See **Fig. 6A** and **Fig. 7B** for details regarding figure layout and **Methods** for a description of the analysis. **(A)** Longitudinal patterns of mutations in Env and development of neutralization breadth. **(B)** Integrated information from the three graphs in **(A)**. 4/4 amino acid changes, 2/2 V1h elongations, and 3/3 V1h glycan additions relevant to a given pseudovirus were detected prior to or concurrent with neutralization breadth being observed in the plasma.

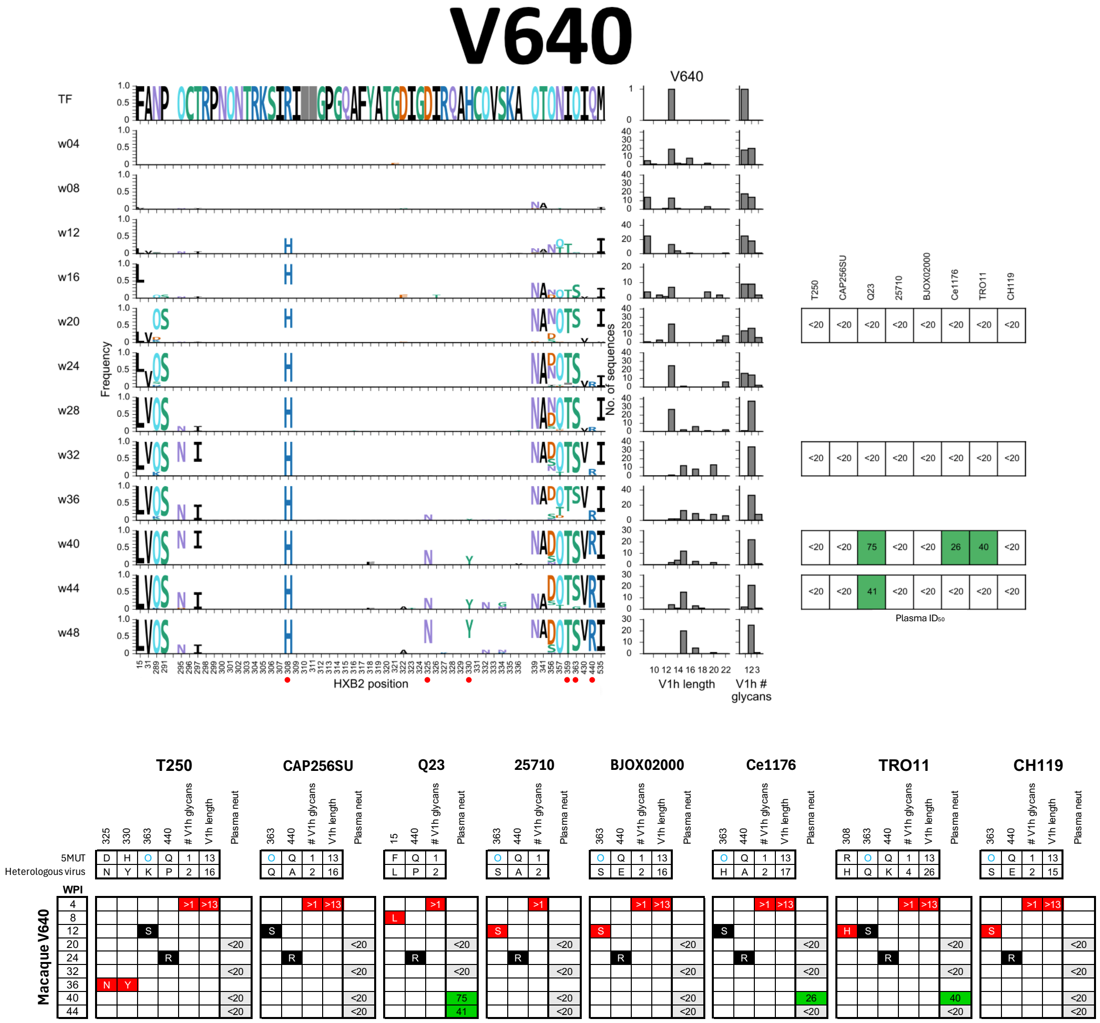

Fig. S29. Env evolution and development of plasma breadth in rhesus macaque V640. See **Fig. 6A** and **Fig. 7B** for details regarding figure layout and **Methods** for a description of the analysis. **(A)** Longitudinal patterns of mutations in Env and development of neutralization breadth. **(B)** Integrated information from the three graphs in **(A)**. 7/7 amino acid changes, 2/2 V1h elongations, and 3/3 V1h glycan additions relevant to a given pseudovirus were detected prior to or concurrent with neutralization breadth being observed in the plasma.

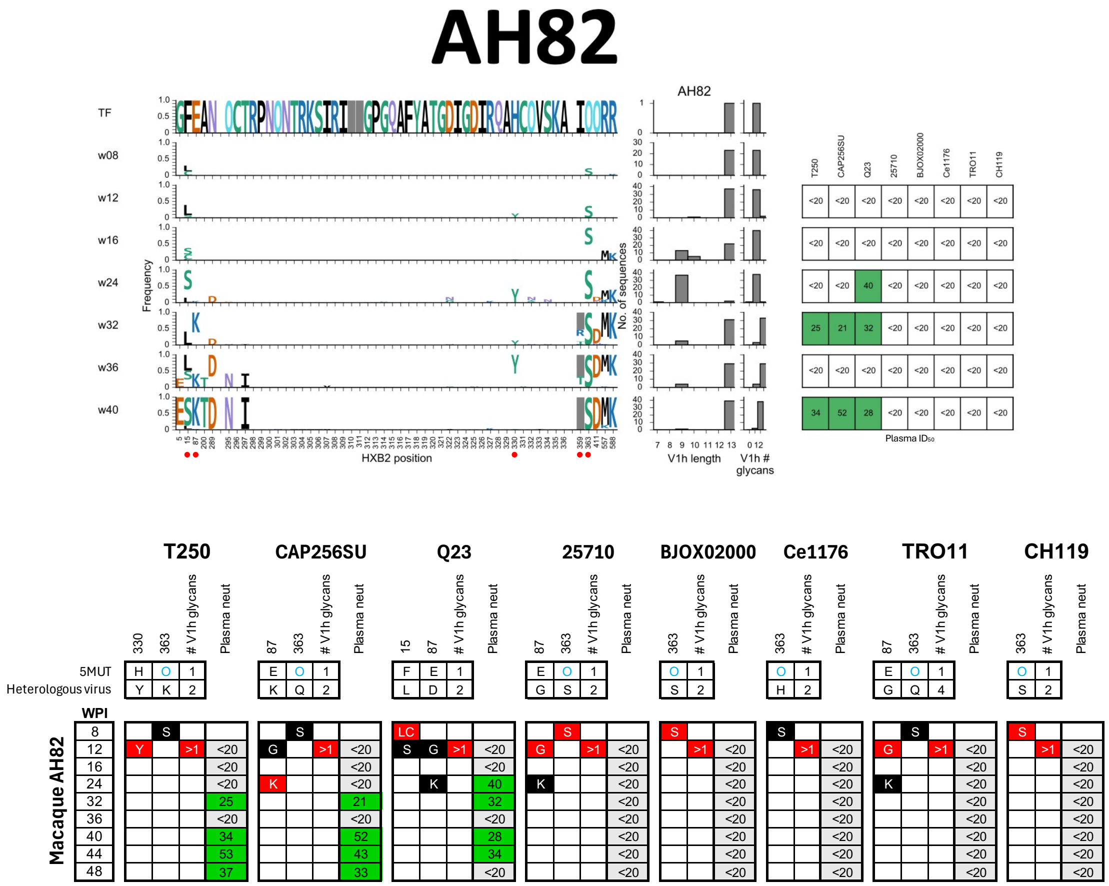

Fig. S30. Env evolution and development of plasma breadth in rhesus macaque AH82. See **Fig. 6A** and **Fig. 7B** for details regarding figure layout and **Methods** for a description of the analysis. **(A)** Longitudinal patterns of mutations in Env and development of neutralization breadth. **(B)** Integrated information from the three graphs in **(A)**. 6/6 amino acid changes and 3/3 V1h glycan additions relevant to a given pseudovirus were detected prior to or concurrent with neutralization breadth being observed in the plasma. O363S was very rare at week 8 but became common by week 24. Addition of a second V1h glycan was first observed at week 12, but this variant was very rare until week 32, coincident with detection of plasma neutralization of T250 and CAP256SU, both of which have two V1h glycans. V1h elongation was not observed within the first 48 weeks of infection in this animal and thus was not included in this analysis. V1h length could be contributing to the limited breadth and potency observed in this animal, although other factors may also play a role. Notably, 25710 has a short V1h loop but is not recognized by the plasma.

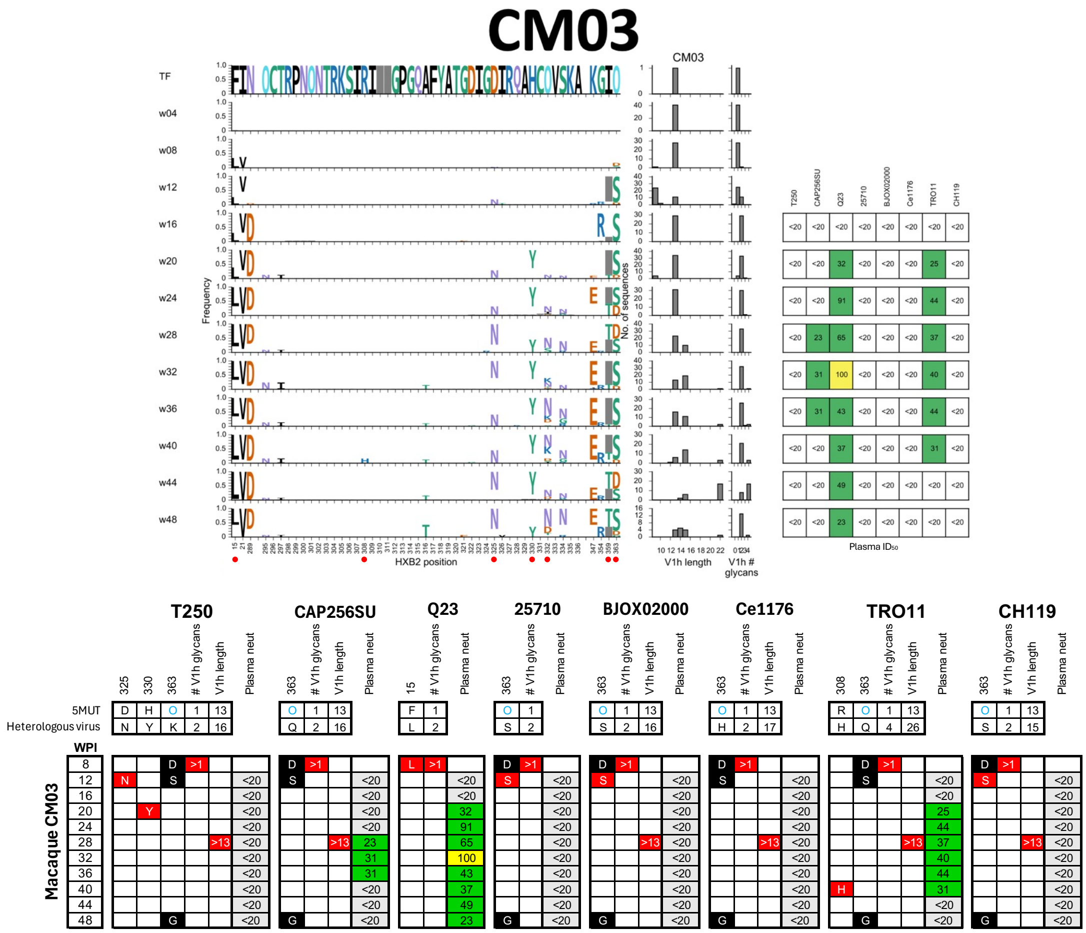

Fig. S31. Env evolution and development of plasma breadth in rhesus macaque CM03. See **Fig. 6A** and **Fig. 7B** for details regarding figure layout and **Methods** for a description of the analysis. **(A)** Longitudinal patterns of mutations in Env and development of neutralization breadth. **(B)** Integrated information from the three graphs in **(A)**. 3/4 amino acid changes, 1/2 V1h elongations, and 3/3 V1h glycan additions relevant to a given pseudovirus were detected prior to or concurrent with neutralization breadth being observed in the plasma. TRO11 was neutralized at week 20 even though the R308H variant was not observed until week 40 and elongated V1 variants were not observed until week 28. While R308H seemed to be a resistance mutation for most other bNAb lineages, neither AH82 nor CM03 showed much selection involving this residue during the first year of infection, suggesting that this site might not be relevant to the bNAb lineages in these two animals. V1-elongated variants were detected concurrently with plasma neutralization of CAP256SU.

Table S1. Rhesus macaque demographic information.

Group	RM ID	Sex	Age	CD8 depletion
1	V630	Male	5	anti-CD8β (25 mg/kg)
	V631	Male	5	
	V632	Male	5	
	V633	Male	5	
	V634	Male	5	
	V635	Male	5	
	V636	Male	5	
	V637	Male	5	
2	V638	Male	5	
	V639	Male	5	
	V640	Male	5	
	V641	Male	5	
	V642	Male	5	
	V643	Male	5	
	V644	Male	5	
	V645	Male	5	
3	V650	Male	5	
	V651	Male	5	
	V652	Male	5	
	V653	Male	5	
	AG99	Female	21	None
	AH82	Female	20	
	AJ09	Female	20	anti-CD8α (25 mg/kg)
	AM12	Female	21	
	CK36	Female	8	anti-CD8β (25 mg/kg)
	CM03	Female	8	
	NN39	Male	3	
	NN58	Male	3	

Table S2. Cryo-EM data collection and processing parameters.

Data collection parameters

Microscope	Talos Arctica	Titan Krios
Voltage (kV)	200	300
Camera	Gatan K3	Gatan K3
Magnification	45000x	105,000x
Frames per movie	40	40
Recording mode	Counting	Counting
Dose rate (e^{-}/pixel/s)	21.234	20.453
Total electron dose (e^{-}/Å^2)	45	60
Defocus range (µm)	-0.8 to -1	-0.8 to -1
Pixel size (Å) (super-resolution)	0.435	0.416
FSC threshold	0.143	0.143

EMDB Maps and Data Processing Parameters

**Antibody**	**Antigen**	**No. of Movies**	**No. of extracted particles**	**Symmetry imposed**	**Map resolution (Å)**	**PDB**	**EMDB**
AJ09-21	5MUT-3fill DS	3483	186,421	C3	3.2	9YHO	EMD-72969
AJ09-83	5MUT-3fill DS	5560	281,473	C3	3.0	9YHQ	EMD-72970
AJ09-110	5MUT-3fill DS	4499	144,442	C3	3.1	9YHR	EMD-72971
AM12-340	5MUT-3fill DS	1065	36,140	C3	3.9	9YHS	EMD-72972
AM12-347	5MUT-3fill DS	2197	115,762	C3	3.6	9YHT	EMD-72973
AM12-351	5MUT-3fill DS	1182	87,733	C3	3.5	9YIB	EMD-72985
AM12-352	5MUT-3fill DS	2291	183,947	C3	3.7	9YID	EMD-72986
V645-158	5MUT-3fill DS	1822	150,394	C3	3.8	9YIE	EMD-72987
V634-136	5MUT-3fill DS	3912	115,334	C3	3.4	9YIF	EMD-72988
V634-136-UCA	del4-3fill DS	4288	235,715	C1	3.8	9YIG	EMD-72989
NN39-25	5MUT-3fill DS	2435	342,870	C3	4.0	9YIH	EMD-72990
NN39-171	5MUT-3fill DS	4889	634,261	C3	3.8	9YII	EMD-72991
N/A	5MUT-3fill DS	1492	548,931	C3	3.8	9YIJ	EMD-72992
N/A	del4-3fill DS	7551	302,556	C3	2.9	9YIK	EMD-72993
N/A	del8-3fill DS	2303	272,897	C3	3.5	9YIL	EMD-72994

Table S3. Human V3-glycan bNAb UCA sequences.

mAb ID	Heavy chain sequence	Light chain sequence	Notes
DH270-UCA3	QVQLVQSGAEVKKPGASVKVSCKASGYTFTGYYMHWVRQAPGQGLEWMGWINPNSGGTNYAQKFQGRVTMTRDTSISTAYMELSRLRSDDTAVYYCARGGWISLYYDSSGYPNFDYWGQGTLVTVSS	QSALTQPASVSGSPGQSITISCTGTSSDVGSYNLVSWYQQHPGKAPKLMIYEVSKRPSGVSNRFSGSKSGNTASLTISGLQAEDEADYYCCSYAGSSTVIFGGGTKLTVL	Reported in ref (19)
BF520-UCA_revised	QVQLVQSGAEVKKPGASVKVSCKASGYTFTGYYMHWVRQAPGQGLEWMGWINPNSGGTNYAQKFQGRVTMTRDTSISTAYMELSRLRSDDTAVYYCARGPFPNYYGSGSYWGGLDYWGQGTLVTVSS	EIVMTQSPATLSVSPGERATLSCRASQSVSSNLAWYQQKPGQAPRLLIYGASTRATGIPARFSGSGSGTEFTLTISSLQSEDFAVYYCQQYNNWPSPFTFGPGTKVDIK	Re-inferred using available data
PCDN76-UCA_revised	QVQLQQWGAGLLKPSETLSLTCAVYGGSFSGYYWSWIRQPPGKGLEWIGEINHSGSTNYNPSLKSRVTISVDTSKNQFSLKLSSVTAADTAVYYCARGGRKVYYDFWSGYVNNWFDPWGQGTLVTVSS	EIVLTQSPGTLSLSPGERATLSCRASQSVSSSYLAWYQQKPGQAPRLLIYGASSRATGIPDRFSGSGSGTDFTLTISRLEPEDFAVYYCQQYGSSPTFGQGTKVEIK	Re-inferred using available data
